# Investigation into the foundations of the track-event theory of cell survival and the radiation action model based on nanodosimetry

**DOI:** 10.1007/s00411-021-00936-4

**Published:** 2021-08-24

**Authors:** Sonwabile Arthur Ngcezu, Hans Rabus

**Affiliations:** 1 University of the Witwatersrand, Johannesburg, 2000 South Africa; 2 Physikalisch-Technische Bundesanstalt (PTB), 10587 Berlin, Germany

**Keywords:** Nanodosimetry, Track structure, Track-event theory, Radiation action model

## Abstract

This work aims at elaborating the basic assumptions behind the “track-event theory” (TET) and its derivate “radiation action model based on nanodosimetry” (RAMN) by clearly distinguishing between effects of tracks at the cellular level and the induction of lesions in subcellular targets. It is demonstrated that the model assumptions of Poisson distribution and statistical independence of the frequency of single and clustered DNA lesions are dispensable for multi-event distributions because they follow from the Poisson distribution of the number of tracks affecting the considered target volume. It is also shown that making these assumptions for the single-event distributions of the number of lethal and sublethal lesions within a cell would lead to an essentially exponential dose dependence of survival for practically relevant values of the absorbed dose. Furthermore, it is elucidated that the model equation used for consideration of repair within the TET is based on the assumption that DNA lesions induced by different tracks are repaired independently. Consequently, the model equation is presumably inconsistent with the model assumptions and requires an additional model parameter. Furthermore, the methodology for deriving model parameters from nanodosimetric properties of particle track structure is critically assessed. Based on data from proton track simulations it is shown that the assumption of statistically independent targets leads to the prediction of negligible frequency of clustered DNA damage. An approach is outlined how track structure could be considered in determining the model parameters, and the implications for TET and RAMN are discussed.

**Supplementary Information:**

The online version contains supplementary material available at 10.1007/s00411-021-00936-4.

## Introduction

The so-called track-event theory (TET) proposed by Besserer and Schneider is a model for predicting cell survival based on the induction of DNA double-strand breaks (DSBs) by charged particle tracks (Besserer and Schneider 2015a, b). The induction of pairs of DSBs within a considered target volume by a particle track is called an “event”. (This is in contrast to microdosimetric terminology where “track” and “event” both refer to the statistically correlated occurrence of energy transfer points (Booz et al. 1983; Rossi and Zaider 1996; Lindborg and Waker 2017)). A low-dose approximation of the fundamental model equation was shown to be equivalent to the commonly used linear-quadratic model and to have a dose dependence that matches the experimentally observed exponential dose dependence at higher doses (Besserer and Schneider 2015a). In later work, the parameters of the model have been related to nanodosimetry (Schneider et al. 2016, 2017, 2019), and recently the TET has been developed into a radiation action model based on nanodosimetry (RAMN) that tried to resolve the shortcomings of the original TET model (Schneider et al. 2020).

In the first version of the TET (Besserer and Schneider 2015a), the basic biophysical model assumption was that a cell will be inactivated if at least two sublethal lesions in the form of DSBs are induced by direct radiation interaction with the DNA. If the two or more sublethal lesions are produced by a single track, this is called a one-track event (OTE). If a track produces exactly one sublethal lesion, then it requires at least two tracks interacting in the cell for its inactivation. This is called a two-track event (TTE). The mathematical formulation of the model further involved the assumption that OTEs and TTEs are “statistically independent events in the terminology of nanodosimetry” (Besserer and Schneider 2015a).

This statement seems paradoxical given that for a particular track and a specific target volume, an OTE and a TTE are disjoint alternatives and, hence, statistically dependent. This contradiction arises from the fact that the terms OTE and TTE were used in two different meanings (Besserer and Schneider 2015a, b; Schneider et al. 2019). Namely, on the one hand, the effect of a particular track on a cell in the sense stated above, and, on the other hand, for the (multi-event) result of the irradiation on the cell. Their mathematical formulation was based on the first meaning of the terms.

In the second version of the TET (Besserer and Schneider 2015b), the model assumption was relaxed by including the possibility of DSB repair, such that cell inactivation occurs only if there are unrepaired sublethal lesions. Repair was assumed to be of “second order”, meaning that DNA repair changes the cell survival rate only for cells with exactly two sublethal lesions. As this introduced an additional model parameter, attempts were made in further work to reduce the number of adjustable model parameters by deriving the ratio of the two model parameters (related to OTEs and TTEs) from chromatin geometry and nanodosimetric properties of ion tracks (Schneider et al. 2016, 2017).

To further reduce the number of model parameters, a first attempt was made in Schneider et al. (2019) to explicitly relate the TET model parameters and nanodosimetric parameters of track structure. This relation was derived by considering OTEs and TTEs in microscopic sites (named “lethal interaction” volumes) within which DSBs are induced in “basic interaction volumes” (BIVs). A BIV is assumed to be a sphere of 2 nm diameter that contains a DNA segment of five to ten base pairs. The size of the (spherical) sites was found to be dependent on radiation type and ranged from 5 nm diameter for carbon ions up to 35 nm for photons.

With the development of the RAMN, some methodological problems with the aforementioned first attempt to relate the TET parameters with nanodosimetry have been overcome. The radiobiological interpretation and the terminology were changed such that now clustered lesions (CLs) and single lesions (SLs) of the DNA are considered (Schneider et al. 2020). The mean frequencies of occurrences of CLs and SLs are linked to the particle fluence, while the (conditional) probability of their induction is related to nanodosimetric parameters of track structure.

This article was motivated by the following concerns of the authors regarding assumptions and methodology used in the TET and RAMN:

1. The observation of inconsistent use of terminology. Apart from already mentioned points like the terms OTE and TTE in the TET model description (Besserer and Schneider 2015a), this also applies to the RAMN model parameter σ. This parameter was initially introduced as an “intersection-cross-section” relating the fluence and frequency of lesions, whereas it was later stated that “σ contains all cell-specific parameters which affect cell sterilization, as e. g. phase in cell cycle, radioresistance, repopulation and repair capability” (Schneider et al. 2020).
2. The assumption of statistical independence of lethal and sublethal (or clustered and single) lesions that seems counterintuitive given that these should be alternative outcomes of radiation interaction (Besserer and Schneider 2015a, b).
3. The apparent contradiction between the concept of particle tracks as statistically correlated interactions and the assumption of statistical independence for single-event radiation effects in different sites (Schneider et al. 2020).
4. The appearance of a term in the repair model that is quadratic in the repair probability and cubic in dose (Besserer and Schneider 2015b).
5. A derivation of model parameters from nanodosimetry that considers only the case of tracks traversing the considered sites (Schneider et al. 2019, 2020). The last point has already been mentioned as one of the limitations of the RAMN in the work of Schneider et al. (2020).

This paper is intended as a critical analysis of the foundations of the TET and RAMN in terms of mathematical consistency of theory and model assumptions as well as with respect to compliance with nanodosimetric results. It is organized as follows. First, the basic TET and RAMN model formula is derived from considerations on the interaction of tracks and biological cells. Furthermore, some conceptional issues are highlighted that arise when linking the cellular-scale picture with subcellular radiation effects. Second, the inclusion of repair in the TET and RAMN is discussed. Third, the approach of Schneider et al. (2019, 2020) to link the TET and RAMN model parameters to nanodosimetric parameters of track structure is discussed with a particular focus on the range of relevant impact parameters. Finally, an outline is given how track structure could be considered in a revised TET/RAMN.

## Theoretical foundations of TET and RAMN

In this Section, the fundamental model equations of the TET and RAMN are derived from an abstract perspective, with a clear distinction between the initial radiation effects at the cellular and subcellular levels and between single-event and multi-event distributions. It should be noted that this derivation is not completely aligned with the formulation of the TET by Schneider et al. (2016, 2017, 2019, 2020), but believed by the authors to be more consistent.

### Derivation of the fundamental model equation

A track (or event in the terminology of microdosimetry) is the set of statistically correlated loci of interactions of a primary particle and all its secondary electrons in a volume of matter. When a (single) track interacts with a biological cell, the radiation-induced damage can be classified into the three categories “lethal”, “sublethal” and “nonlethal”. A lethal event leads to cell inactivation. As this is the result of the interaction of a single track, this was called a one-track event (OTE) in the initial formulation of the TET (Besserer and Schneider 2015a). A sublethal event is not lethal on its own, but when two such events occur (i.e., two tracks interact with the cell), their combination leads to cell inactivation. This was called a two-track event (TTE) in Besserer and Schneider (2015a).

In the case of a nonlethal event by a track, the cell will only be inactivated if one of the following (not disjoint) cases occur: (1) a second track interacts with the cell and produces a lethal event; (2) at least two other tracks interact with the cell and produce sublethal events.

The (single event) probabilities of the occurrence of a nonlethal, sublethal, or lethal event will be denoted in this paper by *p*_0_, *p*_1_, and *p*_2+_, respectively. The quantities *p*_1_ and *p*_2+_ are given by

1 $$p_{1} = \frac{1}{{n_{t} }}{\iint\limits_{A} {p_{c,1} \left( {\boldsymbol{r}} \right)\Phi \left( {{\boldsymbol{r}}{|}D} \right) d^{2} {\boldsymbol{r}}} }$$

2 $$p_{2 + } = \frac{1}{{n_{t} }}{\iint\limits_{A} {p_{c,2 + } \left( {\boldsymbol{r}} \right)\Phi \left( {{\boldsymbol{r}}{|}D} \right) d^{2} {\boldsymbol{r}}} }$$

and *p*_0_ = 1 – *p*_1_ – *p*_2+_. *p*_1_ and *p*_2+_. are the fluence averages of the conditional probabilities, *p*_*c,*1_(***r***) and *p*_*c,*2+_(***r***), that a particle trajectory produces a sublethal or a lethal event, respectively, if the primary particle trajectory passes the point given by the position vector ***r***. *Φ*(***r***|*D*) is the dose-dependent area probability density (fluence) for a track passing this point.

The integrals in Eqs. 1 and 2 extend over an area *A* that is defined by the condition that tracks passing the beam cross section within this area have a nonzero probability of producing lethal or sublethal events in the considered cell.

To avoid the notation becoming too cumbersome, we ignore in Eqs. 1 and 2 that *p*_*c,*1_(***r***) and *p*_*c,*2+_(***r***) also depend on the energy of the ionizing particle producing the track. We also do not consider explicitly that there is a dependence on the direction of motion. (In fact, the probabilities will mainly depend on the impact parameter of the track with respect to the target volume). Furthermore, it is worth noting that Eqs. 1 and 2 work best for heavy charged particles. In the case of indirectly ionizing particles such as photons, one would have to replace the area integral by an integral over a volume in which photon interactions producing secondary electrons contribute to the induction of lesions in the considered cell.

If sublethal and lethal events are assumed to be related to the formation of DNA double-strand breaks (DSBs) and DSB clusters in subcellular target volumes that are caused by ionization clusters in the particle track (Schneider et al. 2016, 2017, 2019, 2020), the probabilities of the occurrence of these effects may be defined in an analogous way as for the cellular events. In this case, the diameter of the area A may be between several hundreds of nm up to more than a µm larger than the diameter of the considered target volume (Braunroth et al. 2020). This will be further investigated in Section “Nanodosimetry in TET and RAMN”.

The probabilities *p*_1_ and *p*_2+_ may be assumed to be almost independent on the absorbed dose *D*, whereas the dose dependence is included in the average number of tracks *n*_*t*_ passing the area *A* (Eq. 3).

3 $$n_{t} \left( D \right) = {\iint\limits_{A} {\Phi \left( {{\boldsymbol{r}}{|}D} \right) d^{2} {\boldsymbol{r}}} }$$

It should be noted that *n*_*t*_ is generally not an integer number; it is the expectation of the probability distribution *P*_*t*_(*n*) of the number *n* of tracks passing area *A* that can produce lethal or sublethal events in the considered cell. For a certain number *n* of tracks passg *A*, the conditional probability *P*_c_(*n*_1_, *n*_2+_|*n*) for simultaneous inction of *n*_1_ sublethal events and *n*_2+_ lethal events is given by a multinomial distribution (Eq. 4).

4 $$P_{c} \left( {n_{1} ,n_{2 + } {|}n} \right) = \frac{n!}{{n_{0} !n_{1} !n_{2 + } ! }} p_{0}^{{n_{0} }} p_{1}^{{n_{1} }} p_{2 + }^{{n_{2 + } }}$$

where $$p_{0} = 1 - p_{1} - p_{2 + }$$. and $$n_{0} = { }n - n_{1} - n_{2 + }$$.

The (multi-event) probability *P*(*n*_1_, *n*_2+_) for *n*_1_ sublethal events and *n*_2+_ lethal events to be produced is then given by:

5 $$P\left( {n_{1} ,n_{2 + } } \right) = \mathop \sum \limits_{n} P_{c} \left( {n_{1} ,n_{2 + } {|}n} \right)P_{t} \left( n \right)$$

If *P*_*t*_(*n*) is a Poisson distribution (with *n*_*t*_ as distribution parameter), *P*(*n*_1_, *n*_2+_) is obtained as

6 $$P\left( {n_{1} ,n_{2 + } } \right) = \frac{{\left( {n_{t} p_{1} } \right)^{{n_{1} }} \left( {n_{t} p_{2 + } } \right)^{{n_{2 + } }} }}{{n_{1} !n_{2 + } ! }}e^{{ - n_{t} \left( {p_{1} + p_{2 + } } \right)}}$$

so that the combined (multi-event) probability of *n*_1_ sublethal events and *n*_2+_ lethal events can be written as the product of the marginal distributions that are thus statistically independent and Poisson distributions. In analogy to the single-event case, cell survival occurs if *n*_1_ ≤ 1 and *n*_2+_  = 0, i.e.,

7 $$S = \left( {1 + n_{t} p_{1} } \right)e^{{ - n_{t} \left( {p_{1} + p_{2 + } } \right)}} .$$

Defining the parameters *p* and *q* as

8 $$p = \frac{{n_{t} \left( D \right) \times p_{2 + } }}{D }  \quad q = \frac{{n_{t} \left( D \right) \times p_{1} }}{D}$$

transforms Eq. 7 into

9 $$S = \left( {1 + qD} \right)e^{{ - \left( {p + q} \right)D}} .$$

Equation 9 has the functional form of the basic TET model formula (Besserer and Schneider 2015a). It should be noted, however, that the parameters p and q in Eq. 9 are the expected mean numbers of lethal and sublethal events per dose, not the number of subcellular DNA lesions.

The derivation of Eq. 9 did not require presuming the (multi-event) distributions of lethal events and sublethal events to be statistically independent and to be Poisson distributed as was done in previous work (Besserer and Schneider 2015a, b; Schneider et al. 2016, 2017, 2019, 2020). Both properties follow from the assumption of the Poisson distribution of the number of primary tracks interacting with the cell. Therefore, it seems that these two model assumptions are dispensable, at least when considering events at the cellular level.

### Comparison with the original TET and the RAMN

The original formulation of the TET (Besserer and Schneider 2015a) suffered from a somewhat unclear terminology. Examples are the confusing use of the term “event” for radiation effects in subcellular targets or the use of the term “TTE” for a track inducing a single sublethal lesion as well as for the occurrence of two tracks inducing sublethal lesions that form a lethal lesion. Furthermore, a TTE in the first sense was identified with a DSB and an OTE with the occurrence of “two lethal DSBs on the same or different chromosomes” (Besserer and Schneider 2015a). Thus, it was unclear whether, for example, three DSBs produced by a single track would be considered as the simultaneous occurrence of an OTE and a TTE or whether this would also count as an OTE.

The mathematical formulation of the model in Besserer and Schneider (2015a) suggests that the case of more than two sublethal lesions was implicitly subsumed when talking about two sublethal lesions. On the other hand, the illustration of the basic interactions considered in the model shown in Fig. 1 of Besserer and Schneider (2015a) suggests that the possibility of more than one track affecting the target volume is considered. At the same time, cases such as a track inducing exactly one or more than two sublethal lesions do not seem to be included.

**Fig. 1:**
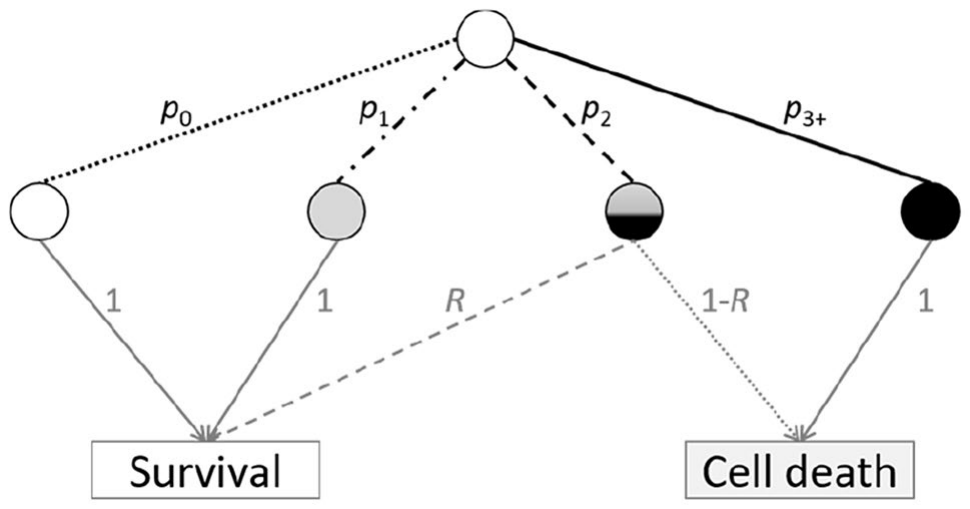
Illustration of the fate of a cell interacting with a single track. The upper open circle symbolizes the cell prior to the radiation interaction. The interaction with the track may be a nonlethal event (dotted line), a sublethal event (dot-dashed line), a potentially lethal event (dashed line), or a definitely lethal event (solid line). In the first case, the cell remains in an essentially unaltered state (open circle) and survives. The second case leads to a cell with a sublethal damage (gray circle) that is repaired with 100% probability (solid gray line). A cell with potentially lethal damage (circle filled half with gray and half with black) has a probability of surviving if the radiation damage is repaired (dashed gray line) and otherwise dies (dotted gray line). A cell with damage from a definitely lethal event dies at 100% probability

The conceptional and terminology problems of the original TET seem to have been overcome with the RAMN. In the RAMN, the fundamental model equation relates the survival of a cell to the average frequency of occurrence of single or clustered DNA lesions (Schneider et al. 2020). The latter is related to the particle fluence and single-event probabilities of the induction of clustered DSBs within subcellular targets. These subcellular targets were called “lethal interaction volumes” in preliminary attempts to derive the ratio of the TET model parameters *p* and *q* (Schneider et al. 2016, 2017) or the absolute parameter values (Schneider et al. 2019) from nanodosimetric parameters of track structure.

Within the RAMN, these (spherical) volumes are called cluster volumes (Schneider et al. 2020). These cluster volumes (CVs) contain an integer number of basic interaction volumes (BIVs). The BIVs have a diameter of 2.5 nm such as to represent a DNA segment of ten base pairs. It is assumed that a DNA lesion in the form of a DSB is induced if at least two ionizations occur within the BIV.

The formalism used in Subsection “Derivation of the fundamental model equation” can also be applied for determining the multi-event frequency distribution of single-track interactions that induce clustered or single DNA lesions in a single nanometric volume. For the case of a (single) subcellular target this approach has also been used in Schneider et al. (2017) and implicitly also in Schneider et al. (2020).

However, there is a (potentially large) number of such subcellular targets. For example, the diameter of the spherical cluster volume best fitting experimental relative biological effectiveness (RBE) data reported in Schneider et al. (2020) for soft X-ray photons was 7.5 nm. Thus, such a volume covers only a small fraction of the volume of the cell nucleus on the order of 2 × 10^–9^. Of course, one has to consider that DNA accounts for only a small fraction of the mass content in the nucleus and that, in addition, chromatin organization may play a role such that certain regions of the chromosome may be more prone to radiation damage (Schneider et al. 2016). However, even if there were only as few as 50 such sites per chromosome, the total number of CVs in a cell nucleus would be on the order of 10^3^.

Therefore, the question arises how the occurrence of DNA lesions in this large number of subcellular targets relates to the induction of lethal and sublethal events at the level of a cell. In the first publication of the TET, “two lethal DSBs on the same or different chromosomes” was the definition of an OTE, i.e., a lethal event at the cellular level (Besserer and Schneider 2015a). In the RAMN, this was replaced with the occurrence of a cluster of DNA lesions (CL) within a nanometric CV. The procedure used in Schneider et al. (2020) for determining the probability of this happening suggests that only a single CV is considered.

In the RAMN it is explicitly assumed that different CVs have the same probabilities of receiving a CL or a SL and that these probabilities are statistically independent (i.e., the probability of obtaining for example a CL in the second CV does not depend on whether there is a CL in the first CV or not).

If different CVs are assumed to be statistically independent, then the convolution of the Poisson distributions of the (multi-event) frequencies of CLs and SLs in all CVs leads to statistically independent Poisson distributions of the number of CLs and SLs per cell. The assumption that “a cell will survive irradiation if no CL [and] at most one SL occurs” then leads to the model Eq. 1 in Schneider et al. (2020). This has the same form as our Eq. 7 but slightly modified as follows:

10 $$S = \left( {1 + N n_{t} p_{SL} } \right)e^{{ - N n_{t} \left( {p_{SL} + p_{CL} } \right)}}$$

where the parameters *p*_*SL*_ and *p*_*CL*_ are the probabilities of the induction of an SL and CL, respectively, in a CV when a single track interacts with the cell. *N* is the number of CVs in the cell and *n*_*t*_ is the dose-dependent number of tracks interacting with the cell.

If the meaning of the parameter σ used by Schneider et al. (2020) is that of a geometrical cross section, Eq. 10 is the same as their Eq. 1.^1^

By adapting the definition of the model parameters in Eq. 8, Eq. 10 transforms again into the fundamental model equation (Eq. 9). The problem is then that the values obtained by Besserer and Schneider (2015a) for the model parameters by fitting to measured survival curves does not corroborate the identification of a sublethal lesion with a single DSB and a lethal lesion with a cluster of DSBs. The values for parameter *q* shown in Table 1 of Besserer and Schneider (2015a) suggest that around one DSB is induced per Gy of absorbed dose, whereas evidence in radiobiological literature indicates that there are generally on the order of several tens per Gy (Ward 1990).

A potential solution to this dilemma may be to consider only severe lesions in the form of complex DSBs. However, such a distinction of DSBs with respect to their complexity has not been considered in the RAMN (Schneider et al. 2020). A second option could be that only a subset of all possible CVs is relevant for radiation-induced cell killing (Schneider et al. 2016). Then, one could hypothesize that a cell survives irradiation if all critical CVs receive at most one SL (and no CL). However, then a cell will survive with a probability *S*

11 $$S = \left( {1 + n_{t} p_{SL} } \right)^{N} e^{{ - Nn_{t} \left( {p_{SL} + p_{CL} } \right)}}$$

where all parameters have the same meaning as in Eq. 10*.* For a large value of *N*, the first factor on the right-hand side of Eq. 11 can be approximated by Eq. 12.

12 $$\left( {1 + n_{t} p_{SL} } \right)^{N} \approx e^{{N\left( {n_{t} p_{SL} - \left( {n_{t} p_{SL} } \right)^{2} /2} \right)}}$$

Similar to Besserer and Schneider (2015a), a second-order Taylor expansion of the logarithm is used here. With this, the survival probability becomes

13 $$S = e^{{ - pD - \left( {qD} \right)^{2} /\left( {2N} \right)}}$$

where the notation of Eq. 9 was re-used with the numerators in Eq. 8 replaced by *N n*_*t*_* p*_*CL*_ and *N n*_*t*_* p*_*SL*_, respectively. If SLs are identified with single DSBs then the quadratic term is negligible for all practically relevant values of dose. The reason is that the average number of DSBs per Gray in a cell is on the order of a few tens (Ward 1990). If *N* is the number of possible CVs, i.e., on the order of 5 × 10^8^, and if 40 DSBs are produced per Gy, then the quadratic term would be unity for a dose on the order of 500 Gy.

If *N* is the number of critical CVs as considered in Schneider et al. (2016), the probability that in a cell a DSB is induced in such a CV is reduced by a factor equal to the ratio of *N* and the number of such possible CVs. Hence, the quadratic term would be smaller by the same factor, as the numerator scales quadratically with this factor. Thus, the quadratic term would be significant only for even higher doses than 500 Gy. Therefore, in the practically relevant dose range up to 80 Gy, the survival curve would be approximately a pure exponential function as for radiation qualities of high linear energy transfer (Goodhead et al. 1993).

Therefore, it seems that the assumption of statistical independence of the probabilities of the induction of SLs and CLs in different CVs does not lead to a model function compatible with radiobiological evidence. Furthermore, it should be noted that for single event distributions, the assumption of statistical independence of CLs and SLs in different targets contradicts the definition of a track as a set of statistically correlated energy transfer points. This will be further investigated in Section “Nanodosimetry in TET and RAMN”.

## Repair

DNA damage repair has not been explicitly addressed in the previous Section. Similar to the original TET in Besserer and Schneider (2015a), however, the notion of sublethal events implicates that the associated damage is repaired. Repair was explicitly introduced in the TET in a second paper by Besserer and Schneider (2015b). The model assumptions with respect to repair were that,

- if exactly one DSB is induced by the irradiation of the cell, this DSB is always repaired,
- if exactly two DNA lesions are induced either by one OTE or two TTEs, they are both repaired with a probability *R*.

In the respective model equation derived as Eq. 7 in Besserer and Schneider (2015b), the factor in front of the exponential in Eq. 9 is replaced by a third-order polynomial in the absorbed dose.

Within the framework of (multiple) tracks interacting with a cell that was adopted in Subsection “Derivation of the fundamental model equation”, the above model assumptions would translate into assuming that radiation-induced damage is,

- always repaired if only one of the tracks interacting with the cell produces a sublethal event while all others are nonlethal events,
- repaired with a probability *R* if one track produces a lethal event and all others are nonlethal events or if two tracks are sublethal events and all other tracks are nonlethal events.

A cell survives if the radiation-induced damage is repaired. Using the probabilities *P* from Eq. 6, the probability *S* for survival is thus given by

14 $$S = P\left( {0,0} \right) + P\left( {1,0} \right) + R\left[ {P\left( {0,1} \right) + P\left( {2,0} \right)} \right]$$

Using Eqs. 6 and 8 this transforms into

15 $$S = \left( {1 + qD + R\left[ {pD + \frac{{\left( {qD} \right)^{2} }}{2}} \right]} \right)e^{{ - \left( {p + q} \right)D}} .$$

Equation 15 differs from the model equations used in the TET (Besserer and Schneider 2015b; Schneider et al. 2017, 2019) by the absence of mixed terms (containing *p* × *q*) and the absence of a term that is quadratic in the repair probability and cubic in dose.

### Critical observations on the TET model with repair

The reason why the approach of Besserer and Schneider (2015b) leads to the additional terms that are not appearing in Eq. 15 is that they seem to have implicitly assumed that the frequency distributions of unrepaired DSBs produced by OTEs and TTEs would also be statistically independent if the frequency distributions of OTEs and TTEs are statistically independent.

This assumption is not plausible, however, as the probability of repair should depend on the total number of DSBs produced in the cell and not how they are produced, as long as they are produced by tracks arriving with a time delay much smaller than the time needed for DSB repair. The latter is on the order of tens of minutes (Metzger and Iliakis 1991), so that for therapeutic beams, the DSBs produced by different tracks can be assumed to occur simultaneously.

Therefore, the outcomes of the irradiation with the same number of DSBs in the cell should be treated in the same way. From Eqs. 4 and 5 in Besserer and Schneider (2015b), the mixed term (containing the product of* p* and *q*) corresponds to the case of survival after two tracks interacted with the target volume; one track produces one DSB which is repaired with probability 1 and the other track two DSBs that are both repaired with probability *R*. The term quadratic in *R* would correspond to three tracks, of which one produces a pair of DSBs that are both repaired with probability *R* while the other two tracks each produce a single DSB and the two DSBs coming from these two tracks are also repaired with a probability *R*.

From the point of view of DNA damage repair, there are two equivalent situations to the first case (mixed terms), namely one track that produces three DSBs or three tracks that each produce one. Similarly, the quadratic term involves four DSBs which would also be obtained by (a) one track producing four DSBs, (b) one track producing three DSBs and a second track producing one DSB, (c) two tracks producing two DSBs or (d) four tracks each producing one DSB. Hence, all these cases would have to be considered as well. However, this would require the respective probabilities to be used as further parameters of the model.

### Consistent DSB-based repair model

To avoid a “Ptolemaic” model with too many parameters, the pragmatic approach taken by Besserer and Schneider (2015b) to assume that up to two DSBs can be repaired and to use only one model parameter for the repair of exactly two DSBs seems advisable. However, the correct functional form of the model curve for such an assumption is different from Eq. 7 in Besserer and Schneider (2015b) and from Eq. 15 above.

The reason for this is that there is implicitly another model assumption involved regarding the relation between the conditions for the lethality of events (i.e., tracks interacting with a cell) and the number of DSBs produced by such tracks. In the work of Besserer and Schneider (2015b), the fate of a cell in which a single track produces more than two DNA lesions has not been explicitly addressed. From their Fig. 1 one may infer that if a track induces four or more DSBs, the cell is killed.^2^ However, if a cell is killed when a track induces four or more DSBs, this implies that one has to consider four categories of events in the repair model (see Fig. 1): nonlethal, sublethal, potentially lethal (i.e. lethal if not repaired), and definitely lethal events.^3^

If the induction of potentially and definitely lethal events occurs with average probabilities *p*_2_ and *p*_3+_, respectively, the conditional probability *P*_c_(*n*_1_, *n*_2_, *n*_3+_|*n*) for simultaneous occurrence of *n*_1_, *n*_2_, and *n*_3+_ tracks inducing sublethal, potentially lethal, and definitely lethal events in the considered cell is given by:

16 $$P_{c} \left( {n_{1} ,n_{2} ,n_{3 + } {|}n} \right) = \frac{{n! p_{0}^{{n_{0} }} p_{1}^{{n_{1} }} p_{2}^{{n_{2} }} p_{3 + }^{{n_{3 + } }} }}{{n_{0} !n_{1} !n_{2} !n_{3 + } ! }}$$

Weighting with the Poisson distribution of the number of tracks leads to the probability distribution *P*’(*n*_1_, *n*_2_, *n*_3+_) as given in Eq. 17.

17 $$P^{\prime} \left( n_{1} ,n_{2} ,n_{3 + } \right) = \frac{{\left( {n_{t} p_{1} } \right)^{{n_{1} }} \left( {n_{t} p_{2} } \right)^{{n_{2} }} \left( {n_{t} p_{3 + } } \right)^{{n_{3 + } }} }}{{n_{1} !n_{2} ! n_{3 + } ! }}e^{{ - n_{t} \left( {p_{1} + p_{2} + p_{3 + } } \right)}} .$$

If potentially lethal events are repaired with probability *R*, a cell survives with probability *S* given by

18 $$S = P^{\prime}\left( {0,0,0} \right) + P^{\prime}\left( {1,0,0} \right) + R\left[ {P^{\prime}\left( {0,1,0} \right) + P^{\prime}\left( {2,0,0} \right)} \right]$$

Using Eq. 8 this transforms into

19 $$S = \left( {1 + qD + R\left[ {p^{\prime}D + \frac{{\left( {qD} \right)^{2} }}{2}} \right]} \right)e^{{ - \left( {p + q} \right)D}}$$

where *p’* is a fourth model parameter which is related to the probability that a track produces a potentially lethal event:

20 $$p^{\prime} = \frac{{n_{t} \left( D \right) \times p_{2} }}{D} .$$

**Fig. 2:**
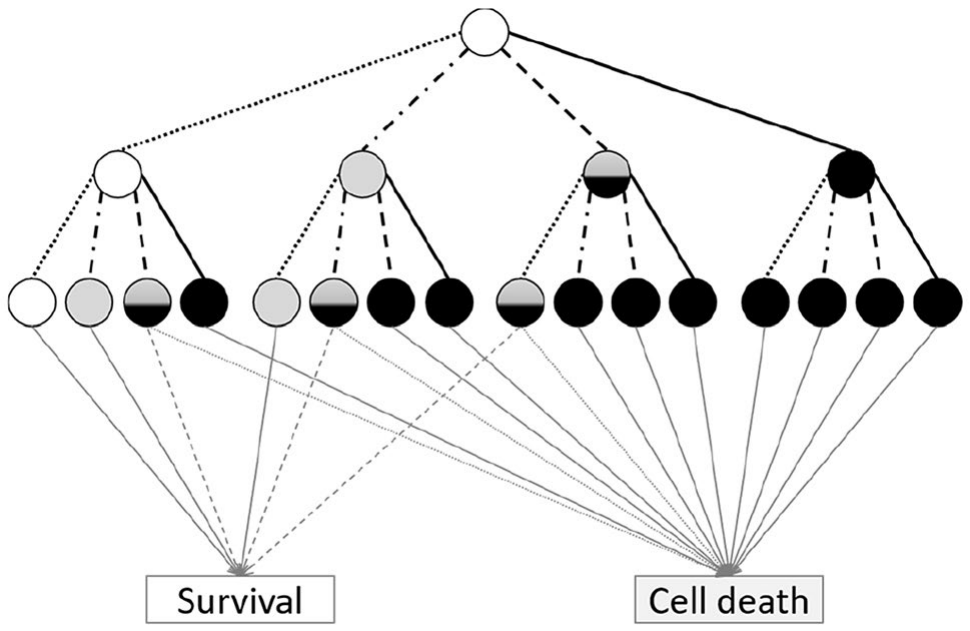
Illustration of the outcome when two tracks interact with a cell. The meanings of the symbols and lines are the same as in Fig. 1

The respective cell fate for the case of exactly two tracks is schematically illustrated in Fig. 2. The third row of circles shows the possible results of the interactions of the two tracks in the cell before repair. The possible results are a cell with nonlethal (open circle), sublethal (gray circles), potentially lethal (half-gray and half-black circles) and definitely lethal (black circles) damage. The solid gray lines indicate 100% repair probability, the dashed gray lines indicate repair with probability *R,* and the dotted lines repair failure with probability (1 − *R*).

Figure 2 can also be seen as an illustration for more than two tracks interacting with the cell if the second row of symbols is interpreted as the cell damage produced by all previous tracks where equivalent cases have been combined.

Furthermore, Figs. 1 and 2 can also be used as illustrations of the repair model that derives from the model assumptions made by Besserer and Schneider (2015b) if one distinguishes between tracks producing exactly two DSBs and those that produce three or more DSBs and assumes that the latter case is a definitely lethal event. Alternatively, one may assume that a definitely lethal event requires four or more DSBs induced by a track. Then the probability *p*_2_ would refer to two or three DSBs produced and *p*_3+_  to four or more DSBs. In both cases, however, the correct model equation is Eq. 19 and not Eq. 7 given by Besserer and Schneider (2015b).

Only if, in contrast to the illustration in Fig. 1 of Besserer and Schneider (2015b), one excludes that a single track can induce a definitely lethal event are the parameters *p*’ and *p* identical and the model has only three parameters. Furthermore, the term quadratic in *R* in Eq. 7 of Besserer and Schneider (2015b) would only appear if damage from different tracks was repaired independently. As repair occurs at a much longer timescale than the production of the damage by the different tracks, there will not be quadratic terms in *R*. In summary, the considerations in this Subsection mean that the model equations for the second version of the TET used in Besserer and Schneider (2015b) and Schneider et al. (2017, 2019) are incompatible with the model assumptions.

### Repair model used in the RAMN

The treatment of repair in the RAMN appears a bit confusing at first glance. The double definition of the model parameter *σ* suggests that the number of CLs and SLs appearing in model Eq. 1 of Schneider et al. (2020) are the number of lesions after repair. This is further suggested by the use of a “persistence parameter” appearing in the expression for the number of SLs that is determined in the appendix of that paper as the ratio of the frequencies of unrepaired SLs and CLs. On the other hand, in the investigation of the dose-rate dependence of cell survival, a repair factor *R* was introduced that affects the probability of SL formation (Eq. 11 in Schneider et al. (2020).

In any case, the fundamental model equation of the RAMN appears to be based on the implicit assumptions that,

- a) a cell survives if there is no unrepaired CL and at most one unrepaired SL,
- b) SLs and CLs are repaired independently with constant probabilities *R*_1_ and *R*_2+_, respectively.

The ratio of the two complementary probabilities, (1 − *R*_1_) and (1 − *R*_2+_), is the “persistence parameter” in the terminology used by Sceider et al. (2020). The wording of assumption (a) above differs from Schneider et al. (2020) in that the condition is not referring to the occurring CLs and SLs, but to the persistent CLs and SLs after repair.

If assumption (b) applies and if *P*(*n*_1_, *n*_2+_) is the (multi-event) probability of the induction of *n*_1_ SLs and *n*_2+_ CLs, then the distribution P^*^(*k*_1_, *k*_2+_) of the numbers *k*_1_ and *k*_2+_ of unrepaired SLs and CLs, respectively, is given by

21 $$P^{*} \left( {k_{1} ,k_{2 + } } \right) = \left( {1 - R_{1} } \right)^{{k_{1} }} \left( {1 - R_{2 + } } \right)^{{k_{2 + } }} \times \mathop \sum \limits_{{n_{1} = k_{1} }}^{\infty } \mathop \sum \limits_{{n_{2} = k_{2} }}^{\infty } \left( {\begin{array}{*{20}c} {n_{1} } \\ {k_{1} } \\ \end{array} } \right)R_{1}^{{n_{1} - k_{1} }} \left( {\begin{array}{*{20}c} {n_{2} } \\ {k_{2} } \\ \end{array} } \right)R_{2 + }^{{n_{2 + } - k_{2 + } }} P\left( {n_{1} ,n_{2 + } } \right).$$

From Eq. 21, it is evident that if the distributions of induced SLs and CLs are statistically independent, i.e., *P*(*n*_1_, *n*_2+_) = *P*_1_(*n*_1_) *P*_2+_(*n*_2+_), then the same is also true for the distributions of persistent SLs and CLs, whether the marginal distributions *P*_1_(*n*_1_) and *P*_2+_(*n*_2+_) are Poisson distributed or not. If they are Poisson distributed, this is also the case for the distributions of *k*_1_ and *k*_2+_.

However, it is important to note that the statistical independence and Poisson distributions for lesions in cells or subcellular targets found in Subsection “Derivation of the fundamental model equation” does not warrant that the distributions of CLs and SLs in a cell also have these properties. The reason is that there is more than one target volume involved and that the statistical independence between different target volumes cannot be inferred from the statistical independence of the tracks interacting with a cell. To assess the relation between distributions of CLs and SLs and those of tracks requires the single event distributions of CLs and SLs to be considered, which brings nanodosimetry into play (cf. Section “Nanodosimetry in TET and RAMN”).

### An alternative repair model

It is plausible that the repair capacity of a cell is limited so that for a large number of DSBs the average probability of an individual DSB to be repaired will decrease. However, it seems rather implausible that this should already be the case for three (or four) DSBs in a cell. In radiobiological assays, often a large number of DSB repair foci are observed (MacPhail et al. 2003; Ponomarev and Cucinotta 2006; Ponomarev et al. 2008; Martin et al. 2013). Hence, it might have been more appropriate to rather assume in the model a constant probability of the repair of an individual DSB. Deriving a respective model equation becomes very intricate, however, as an analytical treatment of this case would require knowledge of all probabilities *p*_k_ for induction of *k* DSBs by a single track.

As this would make the model rather complex, an alternative simple repair model would be to assume that repair with probability *R* occurs whenever there is more than one DSB. Then the probability of cell survival *S*’ would be given by Eq. 22.

22 $$S^{\prime} = \left( {1 + qD} \right)e^{{ - \left( {p + q} \right)D}} + R\left[ {1 - \left( {1 + qD} \right)e^{{ - \left( {p + q} \right)D}} } \right]$$

The trivial reason is that the first term of the sum is the probability that at maximum one DSB is produced so that the term in the square brackets is the probability of more than one DSB. If the other two model parameters can be determined from nanodosimetry, this model Eq. 22 has only one free parameter.

## Nanodosimetry in TET and RAMN

In further work, Schneider et al. elaborated an approach to derive the ratio of model parameters *p* and *q* (Schneider et al. 2016, 2017) or the absolute parameter values (Schneider et al. 2019, 2020) from nanodosimetric parameters of track structure. To determine the absolute values of the parameters, they added the following model assumptions:

- Existence of subcellular target volumes of identical size within which the induction of two or more (unrepaired) DSBs leads to cell death.
- Such a target volume contains a number of “basic interaction volumes” (BIVs) in which a DSB is produced with a probability equal to the nanodosimetric parameter *F*_2_, i.e., the probability of two or more ionizations within that BIV.

The BIVs are assumed to be spheres enclosing a short strand of DNA of five to ten base pairs (Schneider et al. 2019). The sphere diameter was assumed to be 2.0 nm (Schneider et al. 2019) or 2.5 nm (Schneider et al. 2020). The nanometer-sized spherical volumes from assumption (a) were named “lethal interaction volume” in Schneider et al. (2019) and “cluster volume” (CV) in Schneider et al. (2020). The size of the CV was assumed to depend on radiation quality (Schneider et al. 2019).

Based on the two aforementioned assumptions, the probabilities of OTEs and TTEs (within the TET) and of CLs and SLs (in the RAMN) were then derived by binomial statistics. These probabilities were finally used to obtain an expression for RBE (Schneider et al. 2019, 2020).

### Issues with the TET’s and RAMN’s link to nanodosimetry

The preliminary attempt to link the track-event theory with nanodosimetry presented in Schneider et al. (2019) had its deficiencies that have been healed in the RAMN where a similar approach as presented in Subsection “Derivation of the fundamental model equation” was used in which the probabilities of the occurrence of CLs and SLs are given by multiplications of three factors. One is the fluence *ϕ,* while another one is the respective conditional probabilities, *P*_*CL*_ and *P*_*SL*_, for the induction of these lesions in a CV (Schneider et al. 2020). The third factor is the model parameter *σ*, which is defined ambiguously, but appears to be meant as the product of the geometrical cross section of a BIV and the probability of a CL not being repaired. Thus, Eqs. 2 and 3 of Schneider et al. (2020) could be rewritten as

23 $$\overline{CL} = \left( {1 - R_{2 + } } \right) \times \phi \times \sigma \times P_{CL}$$

24 $$\overline{SL} = \left( {1 - R_{1} } \right) \times \phi \times \sigma \times P_{SL} .$$

Equations 23 and 24 are expressions of a form that would also be obtained by inserting Eq. 6 in Eq. 21 and then calculating the mean numbers of persistent SLs and CLs. The difference would be that the number of contributing tracks would relate to a cross-sectional area that is potentially much larger than the cross section of a BIV (cf. Subsection “Probability of inducing an IC in a BIV by proton tracks”). Even if only tracks passing the target region mattered, *σ* would be the cross section of the CV and not of the BIV.

A second issue with the approach used by Schneider et al. (2019, 2020) to derive the model parameters from nanodosimetry is the assumed one-to-one correspondence between DSBs and the formation of ionization clusters.^4^ While this has also been hypothesized in other work (Grosswendt et al. 2005, 2006), comparisons with dedicated radiobiological experiments in work by Garty et al. showed the relation between ionization clusters and DSBs to require the use of a (one-parameter) combinatorial model (Garty et al. 2006, 2010). This was later demonstrated to imply the one-to-one correspondence between the probability of two or more ionizations to apply only approximately and only for low-LET radiation (Nettelbeck and Rabus 2011; Rabus and Nettelbeck 2011). Conte et al. (2017, 2018) and Selva et al. (2019) demonstrated that a link between nanodosimetry and cell survival can be based on cumulative probabilities of ionization clusters, if in addition to *F*_2_ also the probability of clusters with three or more ionizations, *F*_3_, is included in the model.

However, even if the assumption holds that a DSB in a BIV occurs with the same probability *F*_2_ as an ionization cluster is formed by a passing track in this BIV, a further issue arises: the derivation of the parameters *P*_*CL*_ and *P*_*SL*_ in Schneider et al. (2019, 2020) ignores the fact that *F*_2_, *P*_*CL*_ and *P*_*SL*_ are all conditional probabilities. They all relate to the occurrence of the respective radiation effect if a track interacts with the considered target.

If the cross section of the CV and of the BIV is taken as the area used in Eq. 3, the respective mean number of tracks, *n*_*t*_, interacting with the CV or BIV is very small compared to unity and can be interpreted as the probability of a track interacting with the target. The probability of the formation of a DSB in any BIV within the CV is then *n*_*t*_ × *F*_2_. The total probability of an SL and a CL is then given by the right-hand sides of Eqs. 4 and 5 in Schneider et al. (2020) but with *F*_2_ replaced by *n*_*t*_ × *F*_2_. The conditional probabilities are then obtained by dividing with *n*_*t*_, so that the correct expressions for *P*_*CL*_ and *P*_*SL*_ are as follows:

25 $$P_{SL} = F_{2} \times n \times \left( {1 - n_{t} F_{2} } \right)^{n - 1}$$

26 $$P_{CL} = \frac{{1 - \left( {1 - n_{t} F_{2} } \right)^{n} }}{{n_{t} }} - F_{2} \times n \times \left( {1 - n_{t} F_{2} } \right)^{n - 1}$$

where *n* is the number of BIVs traversed by a track intersecting the CV. As *n*_*t*_ is small compared to unity, one can use an expansion of the binomials and discard terms quadratic in *n*_*t*_:

27 $$P_{SL} \approx F_{2} \times n$$

28 $$P_{CL} \approx n_{t} \times n \times \left( {n - 1} \right) \times F_{2}^{2} .$$

Therefore, the magnitude of *P*_*CL*_ derived in this way depends on both the number of BIVs per CV (or per mean chord length through the CV) and the cross-sectional area considered in the determination of *n*_*t*_. This leads to a further potential issue which is related to the determination of the nanodosimetric parameter *F*_2_ from track structure simulations, where the illustrations in Fig. 1 of Schneider et al. (2019) and Fig. 1 of Schneider et al. (2020) suggest that only a central passage of the primary particle through a BIV is considered. This conjecture is corroborated by the number of BIVs in a CV used in the binomial, namely the ratio of the mean chord length in the CV and the BIV diameter.

In the work of Schneider et al. (2020), the simulations were performed for secondary electrons from photon irradiation taking into account the spectral fluence of the electrons. The electron fluence can be expected to be isotropic, so that normal incidence to the BIV surface can be assumed. For determining the probability of CLs, however, it would be better to perform the simulations with the electrons impinging on the surface of a sphere (of diameter equal to a CV) and to score ionizations in all BIVs within this sphere, not only those aligned along the initial direction of motion.

If heavy charged particles (protons, ions) are considered, as was the case in Schneider et al. (2019), one has to take into account that a significant proportion of ionization clusters are produced at radial distances of several tens to several hundreds of nm from the primary particle trajectory (Braunroth et al. 2020; Rabus et al. 2020). For determining the fluence-averaged probabilities of CLs and SLs in a CV, a better assumption would thus be that all BIVs in a CV have the same probability of receiving an ionization cluster. The importance of heavy-charged particle tracks with large impact parameters is demonstrated in the following Subsections.

### Probability of inducing an IC in a BIV by proton tracks

In this Subsection, results are presented for single-event and multi-event averages of the nanodosimetric parameter *F*_2_ for induction of an ionization cluster (IC) in a BIV by passing proton tracks. The methodology used is described in detail in Supplementary Material 1.

In brief, it is assumed that the probabilities of IC formation in different sites are statistically independent and that the dependence of the probability of the formation of an IC in a site, *F*_*2*_(*r*), on the impact parameter *r* of the primary particle trajectory with respect to the center of the site is known.^5^ Spherical sites are considered that are located within a spherical region of interest (ROI) with radius *R*_*L*_. The primary particle trajectory is assumed to pass the ROI within an annulus (see Supplementary Fig. 1) whose inner and outer radii are successive integer multiples of *R*_*L*_.

**Fig. 3:**
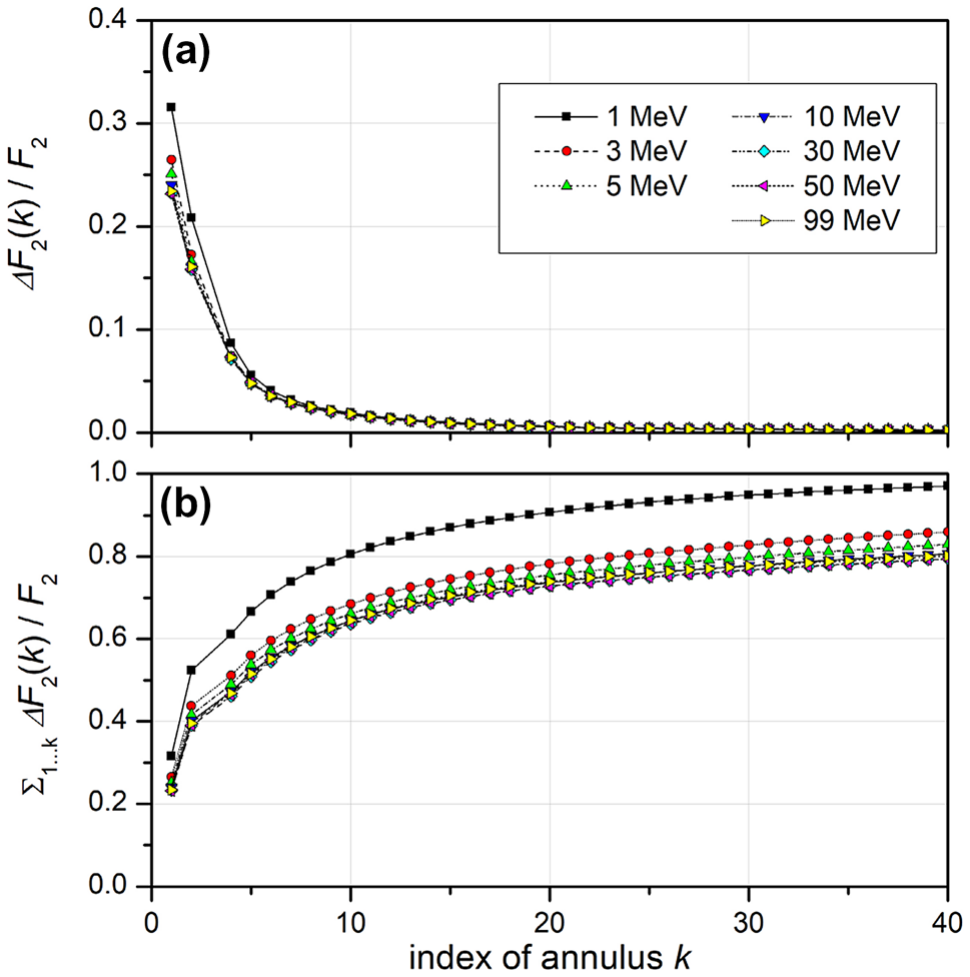
**a** Relative contribution to the total probability *F*_2_ of obtaining an ionization cluster in a BIV of 3 nm from a proton of the energies given in the legend that passes through the *k-*th annulus around the BIV or through the BIV (*k* = 1). **b** Relative contribution from protons passing the first *k* annuli around a BIV to the total probability of obtaining an ionization cluster. (*BIV* basic interaction volume; *F*_2_ probability for induction of an ionization cluster. For details see text).

For determining the probability of induction of an IC in a BIV by proton tracks, the ROI was chosen identical to the site and the site diameter was chosen as 3 nm to have a volume identical to the cylindrical targets used in the analysis of simulated proton tracks by Braunroth et al. (2020). The results for the contributions of the different annuli to the total probability *F*_2_ are shown in Fig. 3a for a number of proton energies, and Fig. 3b shows the respective cumulative contributions. While protons traversing the BIV have the highest contribution to the total probability of inducing an IC in the BIV, about 70–75% of the probability *F*_2_ is due to protons passing the BIV for the considered BIV size of 3 nm diameter. It is to be expected that for smaller BIVs this contribution is even higher.

With the exception of the lowest considered energy of 1 MeV, the contributions of the different annuli are almost independent of energy, and convergence of the cumulative distribution is relatively slow. For an energy of 3 MeV or higher the relative cumulative contribution is seen in Fig. 3b to be below 80% up to the maximum annulus index of 40, which corresponds to an outer radius 60 nm in this case.

Thus, determining the value of *F*_2_ from simulations where the primary particle traverses the BIV is problematic in two respects. One is that considering only traversing tracks leads to a significant underestimation of the actual value that would be obtained in a real broad-beam irradiation. The other is that the values obtained from such simulations are only conditional probabilities and need to be corrected for the probability of such a primary particle traversal to occur.

For a fluence value estimated from the ratio of an absorbed dose of 2 Gy and the mass stopping power of protons,^6^ the total probability of the formation of an IC in a particular BIV is between 1.5 × 10^–6^ and 1.4 × 10^–5^ (depending on proton energy). These values suggest that the probability of simultaneous occurrence of several BIVs within a CV should be negligibly small.

### Frequency of BIVs inside a CV receiving an IC by protons

**Fig. 4:**
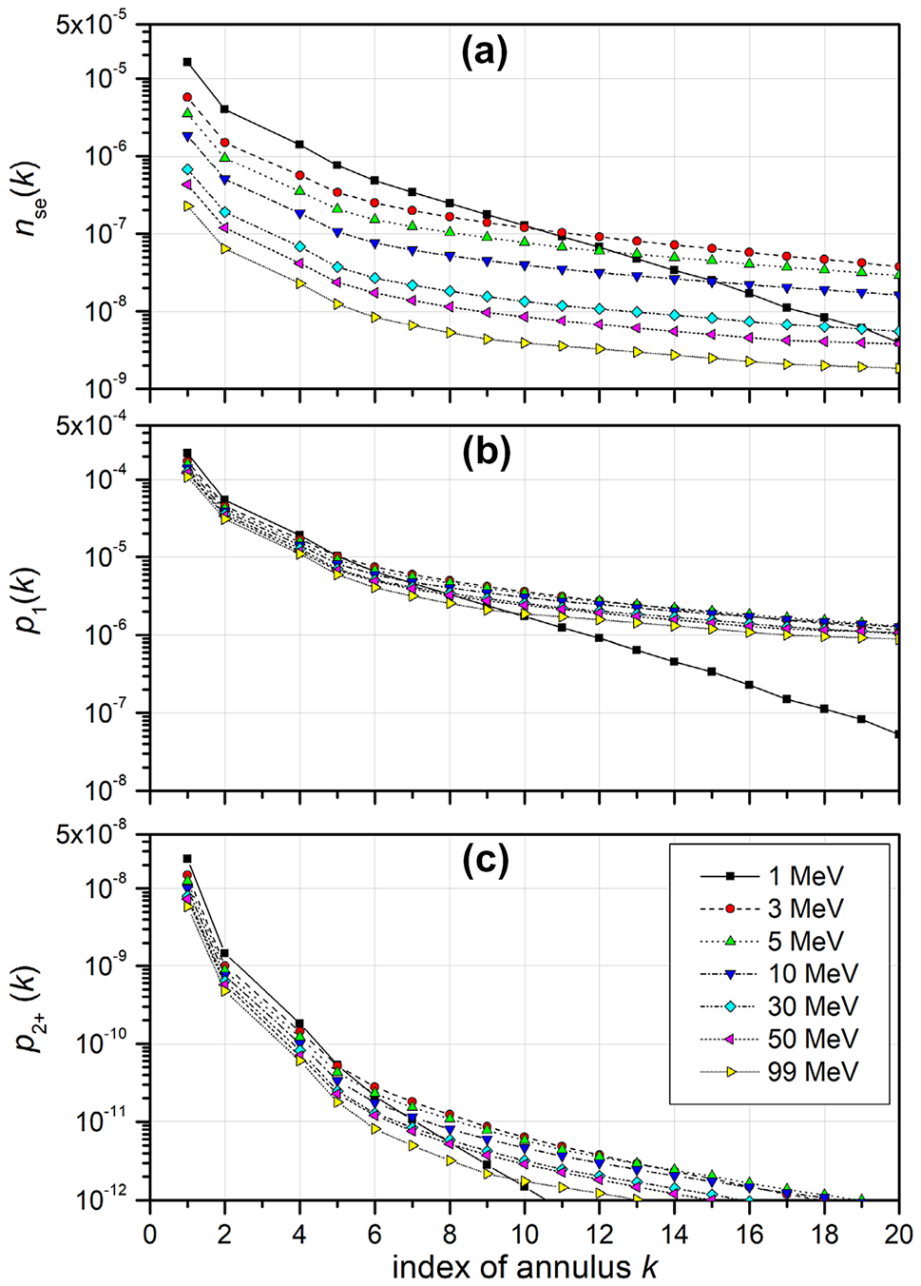
**a** Mean number of BIVs of 3.0 nm diameter inside a CV of 18 nm diameter that receive an ionization cluster when a proton of the energies given in the legend passes through the *k-*th annulus around the CV or through the CV (*k* = 1). The data correspond to a single event, i.e., a fluence of one proton per cross section of a cell nucleus (assumed to have 6 µm diameter). **b** Probabilities that exactly one site in the CV receives an ionization cluster when a proton passes through the *k-*th annulus for an absorbed dose of 2 Gy. **c** Corresponding probabilities of two or more sites in the CV receiving an ionization cluster. cluster. (*BIV* basic interaction volume; *CV* cluster volume; for details see text).

To determine the mean number of sites within a CV that receive an IC from protons passing an annulus around the CV, a ROI diameter of 18.0 nm was chosen that contains the same number of BIVs as the CVs reported by Schneider et al. (2019) for protons. The results are shown in Fig. 4a as a function of the annulus index *k* (ratio of outer radius and *R*_*L*_). The values shown apply to a single event, i.e., one proton passing the cross section of a spherical cell nucleus of 6 µm diameter. It can be seen that the expected number of BIVs with ICs produced by a single event in the considered CV decrease with increasing proton energy and also with increasing annulus index. For the 1 MeV data, the decrease with the annulus index is much more pronounced. This can be explained by the smaller energy transfer to the secondary electrons. It should be noted that the maximum annulus index shown corresponds to a maximum impact parameter of the proton track of 180 nm.

Figure 4b shows the respective multi-event values of the probability *p*_1_ that exactly one site within the CV receives an IC for a proton fluence corresponding to an absorbed dose of 2 Gy, i.e., for a typical treatment fraction in radiation therapy. The maximal values are in the 10^–4^ range so that they approximate well the mean number of sites with ICs for Poisson and binomial distributions. The probability *p*_2+_ of two or more sites in the CV receiving an IC, i.e., that a CL is produced, is shown in Fig. 4c. These probabilities are on the order of 10^–8^ or lower and have been calculated assuming Poisson statistics, but using a binomial distribution would give practically the same values.

The dependence on proton energy is less pronounced in Fig. 4b and c as compared to Fig. 4a, because the fluence corresponding to a value of dose increases with increasing proton energy (at least for energies above the Bragg peak energy of around 80 keV). The relative dependence on the annulus index is naturally the same as in Fig. 4a for the probability *p*_1_, whereas a much stronger decrease with increasing annulus index is observed for probability *p*_2+_. This is expected as ICs formed in different BIVs are assumed to be statistically independent, so that the probability of two or more ICs should be approximately equal to the square of the probability of a single IC if the latter probability is small, as is seen in Fig. 4b.

The pronounced decrease with the annulus index seen in all panels of Fig. 4 implies that the cumulative probabilities converge fast with increasing annulus index (see Supplementary Fig. S4). Therefore, it seems that despite the large proportion of ICs formed at large radial distances seen in Supplementary Fig. S3c, the probability of the formation of two or more ICs in BIVs within a CV (of the sizes used in the present analysis) is mostly determined by proton tracks passing through the CV with a small minor additional contribution from the first real annulus (with outer radius of twice the CV radius). These two regions of impact parameters also account for more than about 80% of the probability of a single IC within the CV. This suggests that, depending on the accuracy aspired, it may be sufficient to consider tracks with impact parameters up to a few times the CV radius when determining the numbers of single and multiple ICs in a CV (SLs and CLs).

It is important to note, however, that there is several orders of magnitude difference between the values of *p*_1_ and *p*_2+_ seen in Fig. 4b and c. This is at variance with the results obtained in the approach of Schneider et al. (2019, 2020) and it also does not seem to be compatible with the values reported earlier for the TET model parameters (Besserer and Schneider 2015a). This indicates that the assumption of statistical independence of the probabilities of IC formation in different targets is not only conceptionally at variance with the definition of tracks and in contradiction to recent experimental evidence for correlated IC formation in adjacent sites (Pietrzak et al. 2018; Hilgers and Rabus 2020), but also leads to a severe underestimation of the probabilities of clusters of ICs (i.e., CLs).

## Outline of a tentative approach to consider track structure in the TET and RAMN

The small absolute values of the probabilities found in Section “Nanodosimetry in TET and RAMN” are due to the fact that fluence averaging has been performed for a single site, where the geometrical relation with the track is generally not known. On the other hand, a track traversing a cell will also traverse or closely pass by some of the sites in the cell nucleus. These close encounters correspond to a locally high value of fluence which, in turn, results in much higher probabilities of the induction of single or multiple ICs within the affected CVs.

Capturing this stochastic process requires a paradigm shift for nanodosimetry that was first proposed by Selva et al. (2018). The further elaboration of these ideas by Braunroth et al. (2020), Rabus et al. (2020), and Rabus (2020) that was used in Section “Nanodosimetry in TET and RAMN” essentially considered amorphous tracks. This Section gives an outline how this paradigm shift for nanodosimetry could be used for the purposes of the TET and RAMN.

### Nanodosimetry of track structure at the micrometer level

For this purpose, the track structure simulation data from Rabus et al. (2020) were analyzed using a development of the methods used by Braunroth et al. (2020) for scoring ICs in the penumbra. In this new approach, a full segmentation of three-dimensional space was performed using the Wigner–Seitz cells of a face-centered cubic Bravais lattice for scoring. A face-centered cubic lattice has a coordination number of 12; its Wigner–Seitz cell is a rhombic dodecahedron which may be considered a reasonable approximation of a sphere.

The scoring approach was used twice. In the first pass, the number of ionizations in the Wigner–Seitz cells were scored. The Bravais lattice constant was chosen such that the volume of the Wigner–Seitz cells was the same as of a sphere of either 2.0 nm, 2.5 nm, or 3.0 nm diameter. The first two dimensions correspond to the BIV sizes assumed in the publications of Schneider et al. (2019, 2020). The third one is the sphere diameter used in Subsection “Probability of inducing an IC in a BIV by proton tracks”, i.e., of the same volume as the cylindrical targets used by Rabus et al. (2020) and Braunroth et al. (2020).

When an IC was found within a Wigner–Seitz cell, the center of gravity of the ionization points in that cell was taken as the position of the IC. In the second pass, the number of ICs was scored within larger cells which had the same volume as spheres of either 12 nm, 7.5 nm, or 18 nm diameter. The first two values correspond to the CV diameters used by Schneider et al. (2019, 2020). The last value is the one used in Subsection “Frequency of BIVs inside a CV receiving an IC by protons”.

The outcome of this scoring was the relative positions with respect to the proton trajectory of CVs in which either a single or multiple ICs were found. In the next step, ROIs in the form of large spheres were placed at different radial distances from the primary particle trajectory and the numbers of CVs with single and multiple ICs inside the ROIs were scored.

The positions of the ROIs with respect to the primary particle trajectories were the centers of cylinder shell sectors around the primary particle trajectory similar to those used by Braunroth et al. (2020). Thus, a segmentation of the ROI’s cross section is obtained that allows the integrals in Eqs. 1 and 2 to be calculated by deterministic sampling. To also account for contributions from primary particle trajectories passing the ROI without intersection, radial distances up to five times the radius of the ROI cross section were included.

Single-event distributions of CVs with single and multiple ICs were determined for spherical ROIs of 500 nm diameter. The restriction in ROI size was imposed by the fact that the simulated proton tracks covered a path length of only 650 nm (Braunroth et al. 2020; Rabus et al. 2020) (The first 100 nm and the distal 50 nm of the track were not used in the analysis).

**Fig. 5:**
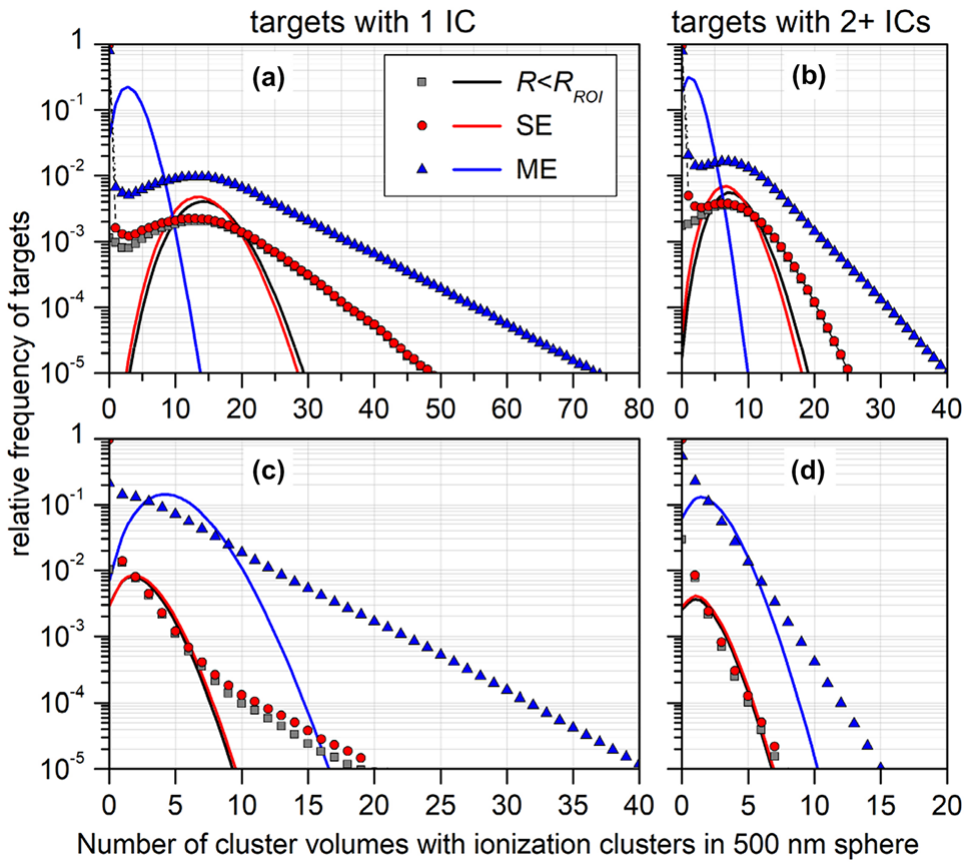
Single-event (SE) and multi-event (ME) distributions of the number of cluster volumes inside a spherical region of interest (ROI) with radius *R*_*ROI*_ = 250 nm that receive a single ionization cluster (IC) (left column) or two or more ionization clusters (right column) from proton tracks. In **a** and **b**, the proton energy is 3 MeV and in **c** and **d** 50 MeV. The spherical cluster volume has 12 nm diameter and the spherical sites 2 nm. The squares indicate the contribution to the SE distribution from tracks intersecting the ROI. The solid lines represent Poisson distributions of the same mean value as the corresponding data marked by symbols when the data point at 0 is omitted. For details see text

Multi-event distributions were obtained by calculating the weighted sum of *n*-fold convolutions of the single-event distributions using the probability of *n* tracks interacting with the ROI as weights. This probability was calculated from Poisson statistics using a primary particle fluence corresponding to a dose of 2 Gy. Results are shown in Fig. 5 as well as in Supplementary Figs. S5–S8. In Fig. 5, results are shown for a BIV of 2 nm and a CV of 12 nm diameter. The top and bottom panels correspond to proton energies of 3 MeV and 50 MeV, respectively. The panels on the left-hand and right-hand sides show the frequencies of cluster volumes with exactly one and more than one IC, respectively. The red circles correspond to the single-event distributions and the blue triangles to the multi-event distributions. The gray squares show the contribution to the single-event frequency coming from proton tracks traversing the ROI. The solid lines represent Poisson distributions with a distribution parameter equal to the mean number of targets obtained for the corresponding data set.

Figure 5a and c show that the frequency distribution of CVs with a single IC has a shape that does not resemble the Poisson distributions obtained using the mean values as Poisson parameter (solid lines). In contrast, the single-event distribution of CVs with more than one IC has some similarity with the respective Poisson distribution, but for the multi-event distributions a non-Poisson shape is observed again. These findings are corroborated by Supplementary Figs. S5 and S6, which show comparisons of the results obtained for 3 MeV and 50 MeV protons, respectively, with the three choices of BIV and CV dimensions. As can further be seen in Supplementary Figs. S7and S8, also for single tracks with a defined impact parameter, the distributions of CVs with exactly one IC are not well described by Poisson distributions. For single tracks traversing the ROI, a Poisson distribution is an approximation for the distribution of CVs with multiple ICs, but with a tail at the right-hand side of the peak that seems to become more pronounced with increasing impact parameter.

**Fig. 6:**
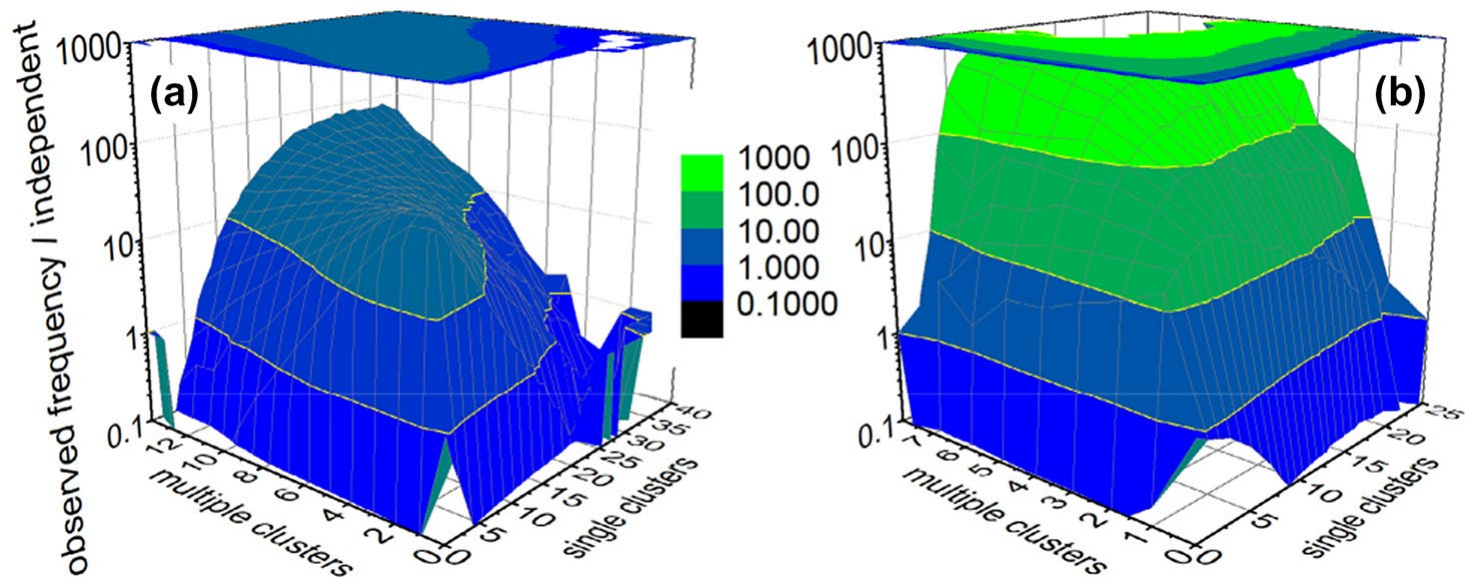
Ratio of the observed frequencies for pairs of numbers of CVs with single and with multiple ICs to the expected frequency for the case that the two marginal distributions are statistically independent. The data have been obtained in a spherical region of interest (ROI) with radius *R*_*ROI*_ = 250 nm and single events of the protons of energy 3 MeV (left) and 50 MeV (right)

To further investigate whether the distributions of CVs with single or multiple ICs are statistically independent, the bivariate distributions of the frequencies of CVs with one and more than one IC have also been sampled. Results for the cases of 3 MeV and 50 MeV proton energy are shown in Fig. 6. The *z*-axis is the ratio of the frequency for simultaneous occurrence of a certain number of CVs with one (*x-*axis) and with more than one IC (*y-*axis) to the product of the marginal probabilities of observing the respective number of CVs, i.e., the data shown in Fig. 5. Statistical independence of the induction of CVs with exactly one or with two or more ICs would be confirmed if this ratio plotted on the *z*-axis has values around unity. However, this is not observed in Fig. 6.

In contrast, values between 10 and 100 are found for most elements of the bivariate distribution for the case of 3 MeV protons. In the case of 50 MeV protons, the values are even an order of magnitude higher, which is presumably due to the fact that the decrease of both marginal frequencies is much faster than for the 3 MeV data. This is to some extent expected as secondary electrons produce ICs at their track ends that may be more important for the sparser ionizing 50 MeV protons.

With respect to the formation of ionization clusters in spherical sites within a ROI, the message of Fig. 5 and Supplementary Figs. S5–S8 is that the respective frequency distributions are not Poisson distributed. And Fig. 6 shows that the frequency distributions of the spherical sites with exactly one or with two or more ICs are not statistically independent. This is essentially reflecting the statistical correlation of the energy transfer points that is at the basis of the definition of events in microdosimetry.

### Track structure at the micrometer level and DSBs

Closer inspection of Fig. 5 and Supplementary Figs. S5 and S6 reveals that the mean number of targets receiving single or multiple ICs is far too high for a 500 nm diameter ROI as compared to the expected number (which is on the order of 30–40) of DSBs in a cell nucleus of ten times larger diameter and, hence, thousand times larger volume. The reason is that not all CV-sized spherical volumes in a cell nucleus contain DNA and thus can be considered to be a target of radiation effects.

The effect of the spatial filtering induced by the sparsity of potential targets has been estimated in this work by assuming that the potential targets have a uniform spatial density within the cell nucleus. If this assumption holds, each CV containing ICs has the same probability *p*_*d*_ for being a “true” target in which ICs lead to DSBs. The conditional probability *P*(*k*_1_, *k*_2+_|*n*_1_, *n*_2+_) that *n*_1_ CVs with one IC and *n*_2+_ CVs with more than one IC result in *k*_1_ CVs with one DSB and *k*_2+_ CVs with two or more DSBs is then given by the product of two binomial probabilities:

29 $$P\left( {k_{1} ,k_{2 + } {|}n_{1} ,n_{2 + } } \right) = B\left( {k_{1} {|}n_{1} ,p_{d} } \right)B\left( {k_{2 + } {|}n_{2 + } ,p_{d} } \right)$$

where

30 $$B\left( {k{|}n,p} \right) = \left( {\begin{array}{*{20}c} n \\ k \\ \end{array} } \right)p^{k} \left( {1 - p} \right)^{n - k} .$$

Inferring the resulting distribution of the number of CVs with single and multiple DSBs from the data obtained for the 500 nm ROIs in Subsection 0 was then done by first determining the distributions of CVs with ICs within a cell nucleus by repeated convolution of the data shown in Figs. 5 and 6. However, this implied the assumption that the ROIs are statistically independent, which may introduce a bias in the results and make them unsuitable for assessing the statistical independence of CVs with single and multiple DSBs.

Therefore, this part of the investigation has been based on simulation data obtained in the frame of the BioQuaRT project (Palmans, et al. 2015). The number of tracks was comparatively small compared to the 50,000 used by (Braunroth et al. 2020): only 50 for 3 MeV protons and 250 for 50 MeV. However, the tracks covered a path of 10 µm (Alexander et al. 2015). Hence, despite the low statistical power, it was possible to study the frequency distribution of CVs with ICs and DSBs for ROIs in the size of a cell nucleus. Here, a ROI diameter of 6 µm has been used and a beam diameter of 9.9 µm. The scoring has been done similar to Subsection “Nanodosimetry of track structure at the micrometer level”. The resulting frequency distributions of CVs with single and multiple ICs are shown in Supplementary Fig. S9 for the BIV and CV dimensions used in Schneider et al. (2019). Similar to what can be seen in Fig. 5, these distributions are also evidently not Poisson distributions.

**Fig. 7:**
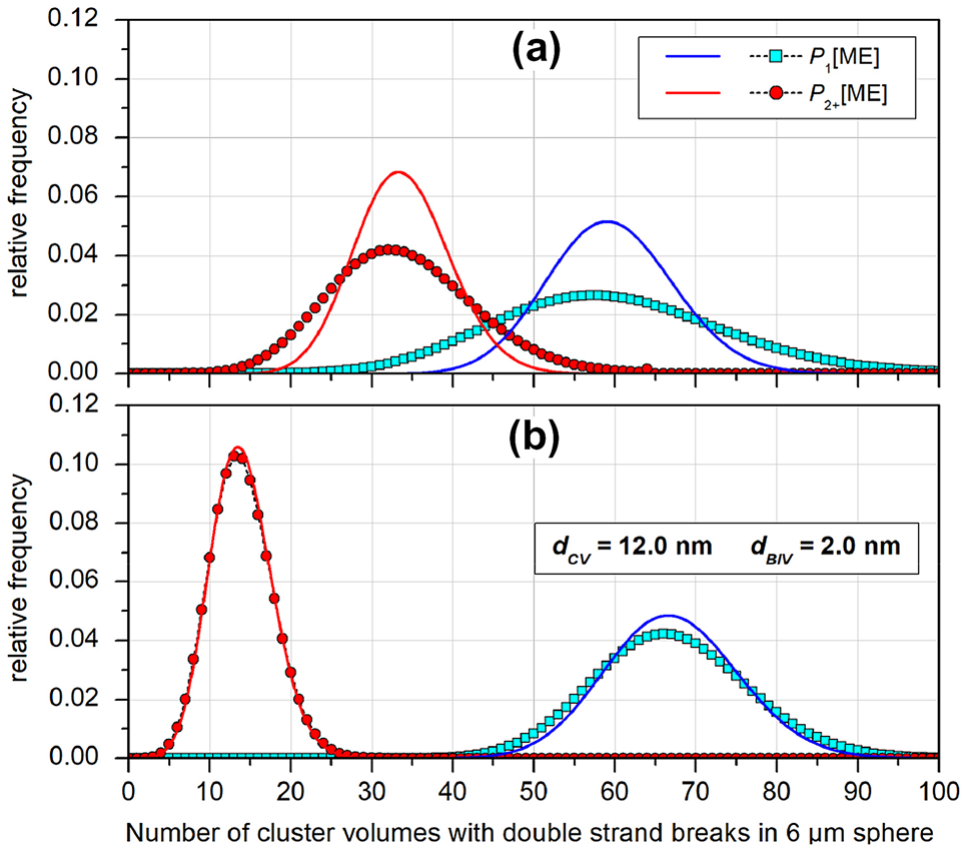
Multi-event (ME) distributions of cluster volumes inside a spherical region of interest (ROI) with radius *R*_*ROI*_ = 6 µm that receive a single DSB (squares) or two or more DSBs (circles) from protons of **a** 3 MeV and **b** 50 MeV energy. The data apply to a particle fluence corresponding to an absorbed dose of 2 Gy and a constant probability of 0.01 for an ionization cluster (IC) to be converted to a DSB. The solid lines are Poisson distributions with the same expectation as the data shown by symbols. (*BIV* basic interaction volume; *CV* cluster volume. For details see text).

Figure 7 shows the distributions of CVs with single (squares) or multiple DSBs (circles) obtained with a value of 0.01 for probability *p*_*d*_. The solid lines indicate Poisson distributions with the same mean value as the data marked by symbols. Contrary to what can be seen in Fig. 5, the distribution of CVs with multiple DSBs for the case of 50 MeV protons in Fig. 7b is seen to be relatively well fitted by a Poisson distribution, whereas the other distributions are overdispersed compared to the related Poisson distributions. This overdispersion is more pronounced for the 3 MeV data and may be related to this radiation quality being more densely ionizing than 50 MeV protons.

**Fig. 8:**
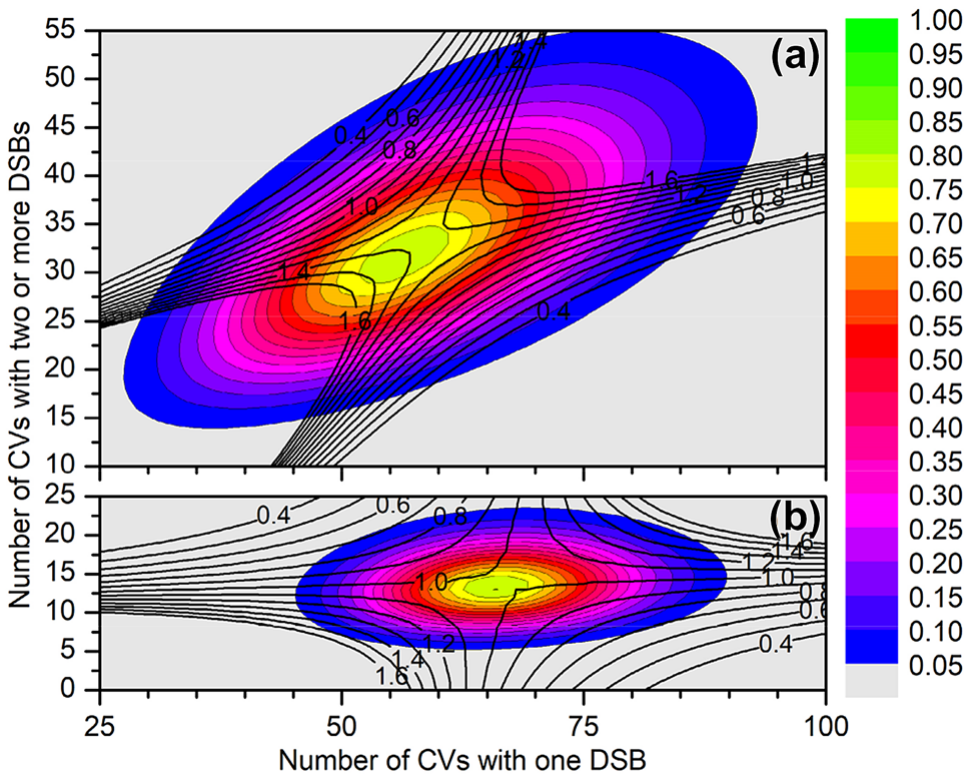
Bivariate frequency distributions of simultaneous occurrence of a number of cluster volumes (CVs) with one DSB (shown on the *x*-axis) and a number of CVs with two or more DSBs (*y*-axis) from protons of **a** 3 MeV and **b** 50 MeV energy. The data apply to a particle fluence corresponding to an absorbed dose of 2 Gy and a constant probability of 0.01 for an ionization cluster (IC) to be converted to a DSB. The colored areas indicate the distribution in increments of 5% of the maximum value. The thick contour lines refer to the ratio of the bivariate distribution to the product of the marginals distributions. For details see text

The bivariate distributions of CVs with single and multiple DSBs for the two proton energies are shown in Fig. 8, overlaid by a contour plot of the ratio between bivariate frequency and the product of the marginal frequencies. The bivariate distribution for 3 MeV protons in Fig. 8a is tilted with respect to the coordinate axes, which suggests that there is a correlation between the occurrence of CVs with single and multiple DSBs. This suggestion is further corroborated by the observation that near the maximum of the distribution, the ratio of the bivariate frequency to the product of the marginal frequencies is between 1.2 and 1.3 and that values of this ratio as high as 6 are found for bivariate frequencies within the top 95% of observed values (see Supplementary Fig. S10a).

In contrast, the bivariate distribution shown in Fig. 8b is aligned with the coordinate axes and in this case the ratio of bivariate frequency to the product of the marginal frequencies is close to unity near the maximum of the distribution and between 0.6 and 1.4 for bivariate frequencies higher than 5% of the maximum (see Fig. 8b). Thus, for this case the distributions of CVs with single and multiple DSBs seem to be statistically independent. Furthermore, the marginal distribution of CVs with multiple DSBs is well described by a Poisson distribution and the distribution of CVs with single DSBs is at least well approximated.

**Fig. 9:**
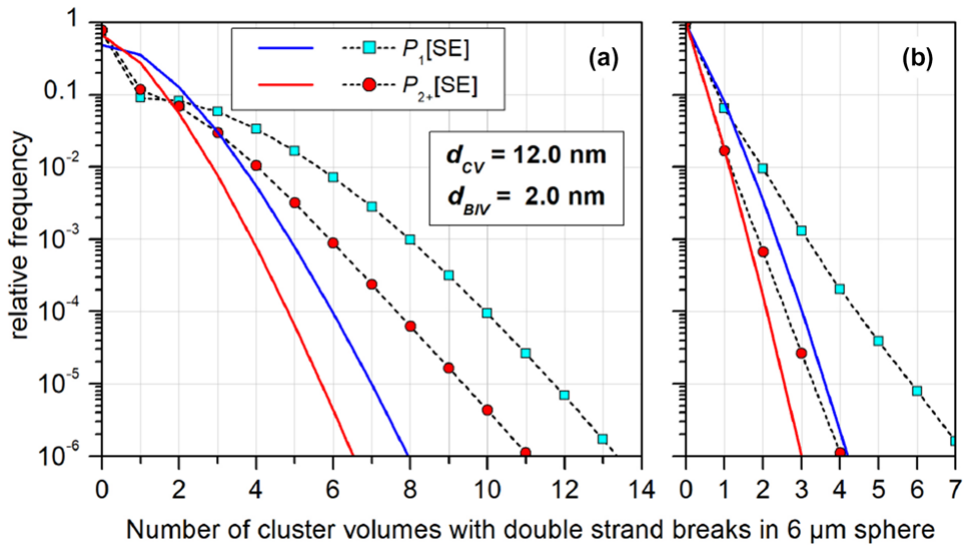
Single-event frequency distributions of cluster volumes (CVs) with one DSB (squares) and CVs with two or more DSBs (circles) from protons of **a** 3 MeV and **b** 50 MeV energy. The data apply to a particle fluence corresponding to an absorbed dose of 2 Gy and a constant probability of 0.01 for an ionization cluster (IC) to be converted to a DSB. The solid lines are Poisson distributions of the same average as the data represented by symbols. For details see text

These observations seem surprising, given the large discrepancy between the distributions of CVs with single and multiple ICs and the respective Poisson distributions of the same mean value (see Supplementary Fig. S9). And they are not explained by the fact that a very small value has been used for the probability *p*_*d*_, so that the binomials appearing in Eq. 29 can be well approximated by Poisson distributions (Schneider et al. 2017). In contrast, the single event distributions of CVs with one and multiple DSBs also show significant discrepancies from the respective Poisson distributions of the same mean value (cf. Fig. 9). However, the deviations from the Poisson distributions are more pronounced for the more densely ionizing 3 MeV protons.

For the 50 MeV protons the average number of tracks corresponding to a dose of 2 Gy and the considered beam diameter of 9.9 µm is about 800. If the bivariate single-event distribution is convoluted 800 times with itself, the result converges to a curve resembling a Gaussian. As can be seen in Supplementary Figs. S11 and S12, however, the overdispersion with respect to the respective Poisson distribution seems to be independent of dose. This suggests that the assumption of statistical independence may be justified for sparsely ionizing radiation, and that, in this case, the frequency distributions of CVs containing single and multiple DSBs may be assumed to have a Poisson-like shape.

## Discussion

The results shown in Fig. 7 are the predicted number of CVs in which single or multiple DSBs are produced. Therefore, the next methodological step along the lines of the TET/RAMN would be to include the repair of DSBs to derive the respective distributions of unrepaired single DSBs and DSB clusters. In principle, this can be done in the same way as in Subsection “Track structure at the micrometer level and DSBs”. Following the approach of Schneider et al. (2020), one has to consider different repair probabilities of SLs and CLs. This would then be equivalent to using two different compound probabilities of the production and non-repair of single and clustered DSBs.

To separate physical and biological radiation effects, as proposed by the BioQuaRT project (Palmans, et al. 2015), it would be more consistent to maintain separate parameters for the spatial density of target volumes and for the repair of single and multiple DSBs. Similar to the approach of Schneider et al. (2019), a large number of cell irradiation experiments could be analyzed with a model that considers two cell-line-specific parameters for repair and three cell-line-independent parameters: the parameter *p*_*d*_ for the target density and the physical parameters used for scoring ICs and clusters of ICs, namely the diameters of the BIVs and CVs, *d*_*BIV*_ and *d*_*CV*_. In the work of Schneider et al. (2019, 2020), the value of *d*_*BIV*_ was always set by a model assumption, but it would be more convincing if the parameter value (or its likelihood distribution) could be inferred from radiobiological data rather than arbitrarily chosen in the range of possible values compatible with existing evidence.

The essential model assumption would be that *d*_*BIV*_ is independent of the biological system and the radiation quality since it is related to the properties of the DNA molecule. It is very likely that also *d*_*CV*_ could be assumed to be independent of both radiation quality and cell type. (The latter would be accounted for by the repair parameters).

The elaboration of a revised RAMN based on a comprehensive characterization of particle track structure is a major endeavor and, hence, beyond the scope of this work, which focusses for the rest of the article on a few methodological aspects.

### Connection between ICs and DSBs

The approach presented in Subsection “Track structure at the micrometer level and DSBs” has similarities with the combinatorial model of Garty et al. (2006, 2010), where the parameter used in the binomial was the conditional probability of an ionization to result in a DNA (single) strand break. In a second step, they considered a random distribution of the strand breaks over the DNA double helix to derive the probability of the formation of a DSB. The analysis in Subsection “Track structure at the micrometer level and DSBs” was, however, based on identifying a BIV with an IC with a DSB. As discussed in Subsection “Issues with the TETs and RAMNs link to nanodosimetry”, this is at variance with evidence for IC complexity (number of ionizations) to play a role (Nettelbeck and Rabus 2011; Rabus and Nettelbeck 2011; Conte et al. 2017, 2018; Selva et al. 2019).

Following the line of arguments of Garty et al. (2006, 2010), a better approach would be to use the following hypothesis: an IC in a short segment of the DNA double helix (represented by the BIV) leads to a DSB if ionizations occur on both strands of the DNA. If only the number of ionizations in the BIV are known, it is straightforward to assume that each ionization has a probability of 0.5 to occur on one strand or the other. Then the conditional probability of the formation of a DSB in a site on the DNA where an IC occurs is given by Eq. 31.

31 $$P\left( {DSB{|}IC} \right) = \frac{1}{{F_{2} }}\mathop \sum \limits_{k = 2}^{\infty } \frac{1}{{2^{{\left( {k - 1} \right)}} }}F_{k} .$$

### Relevant length scales and model parameters

As has already been discussed by Schneider et al. (2020), a RAMN needs to consider interactions of distant (single) lesions as is done in some other approaches to connect microscopic radiation effects and cellular outcome, such as the BIANCA model (Ballarini et al. 2014). The relevance of radiation action on both the micrometric and nanometric scales has been the hypothesis underlying the generic multi-scale model of the BioQuaRT project (Palmans et al. 2015) and demonstrated later by radiobiological evidence (Friedrich et al. 2018).

The extension of the approach outlined in Section “Outline of a tentative approach to consider track structure in the TET and RAMN” toward also including frequency distributions in subcellular volumes, for example of CVs with single ICs, is straightforward. The disadvantage is that further model parameters are introduced. However, the evidence presented by Friedrich et al. (2018) and the success of the BIANCA model (Carante et al. 2018) make such a future extension of the RAMN probably a necessity. The approach presented in Section “Outline of a tentative approach to consider track structure in the TET and RAMN” can be easily extended to include clustering at different spatial scales. And the number of extra parameters coming into play can be handled by assuming them as independent of cell type and radiation quality and taking a big-data approach, as already done to some extent by Schneider et al. (2019).

### Limitations

The approach outlined in Section “Outline of a tentative approach to consider track structure in the TET and RAMN” overcomes two of the limitations of the RAMN discussed by Schneider et al. (2020), since it neither considers only straight segments of tracks in the BIV or CV nor ignores the extension of tracks. Like the RAMN, the present approach also relies on the CVs being homogeneously filled with DNA such that ionization clusters within the CV can be interpreted as DSBs. It does not consider the actual spatial arrangement of DNA in the cell nucleus that may have a role in the formation of DSBs (Kellerer and Rossi 1978; Schneider et al. 2016) and the contribution of radiation damage due to free radicals from water radiolysis.

Furthermore, the limitations discussed by Schneider et al. (2020) regarding the role of different biological endpoints and interference of pathways leading to them as well as interactions between complex and simple DNA lesions also apply. It should be noted, however, that the mean values of CVs with single and multiple DSBs (cf. Fig. 7) are compatible with the rule of thumb that 30 to 40 DSBs are induced per Gy, if an average DNA content in the cell nucleus on the order of 1% is assumed (Goodhead and Brenner 1983). (The multi-event distributions have been calculated for a dose of 2 Gy).

In addition, there are three potential limitations inherent to the scoring procedure used. First, in the calculation of the multi-event distributions, the possibility of several tracks interacting in the same CV has been ignored. However, this is justified, since the probability of this occurring has been shown in Subsection “Frequency of BIVs inside a CV receiving an IC by protons” to be negligibly small. Second, the spherical target volumes (for the formation of DSBs as well as DSB clusters) are approximated by polyhedrons of the same volume. As it has been shown by Grosswendt (2002) that the geometric shape of the target volume has only a minor influence on the IC distributions, this should not be a major issue.

The third limitation is that a regular array of such target volumes is used. This may potentially introduce bias toward a smaller probability of IC formation and toward smaller clusters of ICs within a CV. As the face-centered cubic Bravais lattice has octahedral symmetry, this shortcoming of the scoring geometry could be overcome to a major extent by considering different orientations of the track with respect to the lattice within its small fundamental domain and additionally considering different impact parameters.

However, it should be noted that the procedure outlined in Section “ Outline of a tentative approach to consider track structure in the TET and RAMN” is not reliant on the particular scoring method, which may be substituted in the future with more sophisticated techniques from database analysis (Francis et al. 2011; Bueno et al. 2015).

## Conclusions

The track event model was developed as an alternative model for the dose dependence of cell survival that takes into account the radiation quality by including properties of particle track structure in the form of nanodosimetric probabilities of ionization cluster formation. The radiation action model based on nanodosimetry of Schneider et al. (2020) has been a development that tried to overcome some of the deficiencies of the TET by rebuilding the link to radiobiology.

The original version of the TET produced a model equation (cf. Eq. 9) which offered the advantage of a functional shape that is equivalent to the linear-quadratic model in the dose range in which the latter describes the trend of experimental data well and is superior to it at higher doses where a pure exponential dose dependence is observed experimentally (Besserer and Schneider 2015a).

In this article, it has been shown that some of the assumptions in the original model are dispensable: the Poisson statistics of the frequency distributions of OTEs and TTEs or SLs and CLs and their statistical independence can be derived from an assumed Poisson distribution of the number of tracks contributing to the numbers of OTEs and TTEs or SLs and CLs formed in the considered target volume.

On the other hand, it has also been found that the formula used within the extension of the TET for repair (Besserer and Schneider 2015b) is not consistent with the underlying model assumptions. The correct model equation has been derived in this work and includes a further model parameter, namely the probability that repairable lesions are produced. This parameter can only be ignored if one assumes that there are no single-event lesions that are unrepairable. But even in this case, the model equation is different from the one used in the TET. Unfortunately, this fault in the mathematical model makes the comparison of the TET model with experimental data and the assessment of its performance with respect to the linear-quadratic model questionable. This deficiency of the repair model became obsolete when the TET was replaced with the RAMN.

It has further been demonstrated that the implicit assumption of independent subcellular targets leads to a survival model that is almost purely exponential for relevant dose ranges. This was further corroborated by an evaluation of the probabilities of single and multiple ICs from nanodosimetric simulations for protons, which showed that the probability of multiple ICs would be negligibly small for amorphous tracks and CV dimensions analogous to those used by Schneider et al. (2019, 2020). This evaluation further revealed that with proton tracks more than 50% of the probability of the formation of an IC in a spherical BIV of 3 nm diameter is due to tracks passing the BIV at impact parameters larger than ten times the BIV radius. For proton energies of 3 MeV and higher, more than 25% of the total IC probability comes from impact parameters larger than fifty times the BIV radius.

On the other hand, only tracks passing at impact parameters up to three times the CV radius contribute to the probability of the formation of several ICs within a spherical CV of 18 nm diameter. For single ICs in the CV, impact parameters up to about ten times the CV radius contribute. This suggests that for amorphous tracks and independent BIVs the central passage used in the simulations of Schneider et al. (2019, 2020) to determine model parameters from nanodosimetry should be replaced by simulations where impact parameters up to about 100 nm are considered.

The probabilities of CVs with multiple ICs were found to be negligibly small when IC formation in different targets was assumed to be statistically independent. This confirms that statistical correlations of interactions within particle tracks must be taken into account to obtain reasonably large probabilities of CLs. This has been shown in Section “Outline of a tentative approach to consider track structure in the TET and RAMN” where a paradigm shift was applied for nanodosimetry: instead of considering IC formation in defined targets, the spatial distribution of targets with ICs was used to obtain frequency distributions in micrometric volumes of CVs with single and multiple BIVs with an IC.

Assuming a constant probability that the ICs in the CVs occur within DNA, the frequency distributions of CVs with single and multiple DSBs can be obtained. Using this approach for proton tracks revealed large deviations of the frequency distributions for CVs with ICs from Poisson distributions and a strong correlation between the frequencies of CVs with single and multiple ICs. Despite this, the resulting distributions for CVs with single and multiple DSBs for sparsely ionizing radiation were found to be almost statistically independent and to have shapes that can be roughly approximated by Poisson distributions. For densely ionizing radiation, the frequency distributions of CVs with single and multiple DSBs were found to remain correlated and strongly departing from the Poisson shape. For both sparsely and densely ionizing protons, the deviation between actual distribution and the corresponding Poisson distribution was found to be invariant with dose.

In summary, the analysis presented here has revealed some inconsistencies and weaknesses of the TET and RAMN, but also determined that some of the precarious assumptions made in their development, such as the statistical independence of relevant targets, only seem to contradict the concept of particle tracks, but are at least approximately true for sparsely ionizing radiation. The decisive ingredient of a revised TET/RAMN appears to be a consistent description of the relation between tracks interacting with cells and radiation action in subcellular targets. This requires a paradigm shift from the single-target perspective of nanodosimetry to a track-oriented view. The first steps toward this goal have been outlined in Section “Outline of a tentative approach to consider track structure in the TET and RAMN” The results seem very promising and warrant further endeavor in this direction.

## Supplementary Information

Below is the link to the electronic supplementary material.

Supplementary file1 (PDF 4327 KB)

Supplementary file2 (PDF 356 KB)

## Abbreviations

BIV: Basic interaction volume
CL: Clustered lesions
CV: Cluster volume
DSB: Double strand break
OTE: One-track event
RAMN: Radiation action model based on nanodosimetry
ROI: Region of interest
SL: Single lesion
TET: Track-event theory
TTE: Two-track event

## Footnotes

1 The parameter is introduced by Schneider et al. (2020) as “intersection-cross-section”, and it is stated that its product with the particle fluence is “probability that a particle track intersects any BIV in the cell nucleus”, which suggests a geometrical interpretation. But a few lines below this quote, it is said that “σ contains all cell specific parameters which affect cell sterilization, as e. g. phase in cell cycle, radioresistance, repopulation and repair capability”, which suggests a completely different meaning.

2 The case of three DSBs produced cannot be inferred and may have been assumed to equal the simultaneous occurrence of a DSB pair that is repaired with probability R and a single DSB that is repaired with probability 1.

3 Assuming a repair probability implies that the considered events are no longer lethal but only potentially lethal. As there may be radiation-induced damage of different complexity, the possibility of definitely lethal events that cannot be repaired appears plausible.

4 In the terminology of nanodosimetry, the ionization cluster size is the number of ionizations in a considered target volume, which may also take the values zero or one. As only two or more ionizations constitute a cluster of ionizations, term ionization cluster is used here for this case only.

5 This impact parameter is equal to the magnitude of the position vector ***r ***in Eqs. 1–3 if the center of the target volume is chosen as the origin of the coordinate system and lies on a plane perpendicular to the primary particle trajectory that contains area *A*.

6 The resulting fluence values are between 4.8⋅10^–7^ nm^−2^ and 1.7·10^–5^ nm^−2^ for the proton energies considered.
