# Supplementary material for: Investigation into the foundations of the track-event theory of cell survival and the radiation action model based on nanodosimetry
Source: Radiat Environ Biophys. 2021 Aug 24;60(4):559–78. doi: 10.1007/s00411-021-00936-4 (PMC8551112; doi:10.1007/s00411-021-00936-4)
Supplement: Supplementary file 1 — Supplementary file1 (PDF 4327 KB) [file 411_2021_936_MOESM1_ESM.pdf]

Supplemental material to "Investigation into the foundations of the track-event theory of cell survival and its link to nanodosimetry" in Radiation and Environmental Biophysics

Sonwabile Arthur Ngcezu[1], Hans Rabus[2,*]

[1] *University of the Witwatersrand, Johannesburg, 2000, South Africa*
[2] *Physikalisch-Technische Bundesanstalt (PTB), 10587 Berlin, Germany*
* email: hans.rabus@ptb.de

Supplementary Figures for Section "Nanodosimetry and RAMN" and Methodology used for producing the data

In this Section, an approach is presented to determine single-event and multi-event averages of the nanodosimetric parameter F_2 for induction of an ionization cluster (IC) in a basic interaction volume (BIV) as well as for the number of BIVs in a cluster volume (CV) that receive an ionization cluster (IC). The approach assumes that the probabilities of IC formation in different sites are statistically independent, and it requires knowledge of the dependence of the probability of the formation of an IC in a site, $F_2(r)$, on the impact parameter r of the primary particle trajectory with respect to the center of the site.

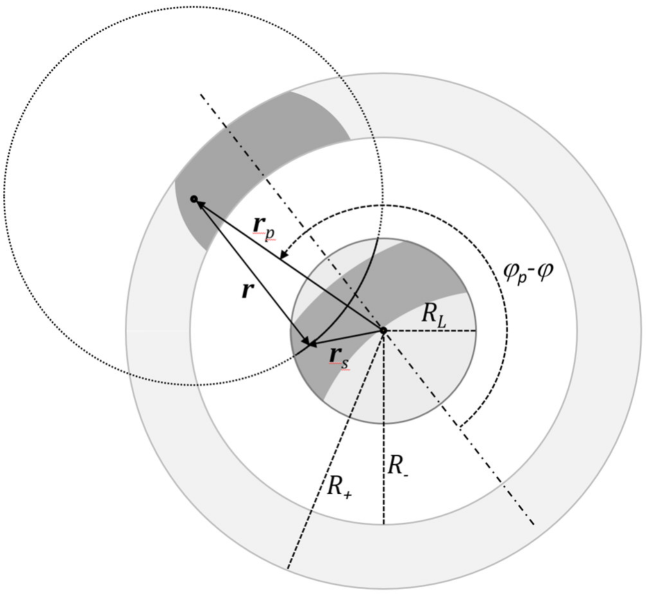

Fig. S1: The shaded circle represents a central cross section of the spherical CV (radius R_L). The small filled black circle (dot) indicates a primary particle trajectory (perpendicular to the drawing plane) that passes the CV at the point described by vector $\boldsymbol{r_p}$ within an annulus of inner radius R_- and outer radius R_+. The solid circle segment inside the CV cross section indicates the loci of sites within the CV that have a radial distance r from this primary particle trajectory. The dark-shaded part of the annulus represents all primary trajectory positions for which the endpoint of a radial vector $\boldsymbol{r}$ that is parallel to the dot-dashed line is within the CV cross-section. The loci of these endpoints cover the dark-shaded area within the CV cross section.

For this purpose, spherical sites are considered that are located within a spherical region of interest (ROI) with radius R_L. Furthermore, the primary particle trajectory is assumed to pass the ROI within an annulus of inner radius R_- and outer radius R_+ in a plane perpendicular to the trajectory that passes through the ROI center (see Fig. S1). The expected number of sites $\bar{n}$ within the spherical ROI that receive an IC when the primary particle passes the annulus is then given by Eq. (S1).

$$\bar{n} = \oint \int_0^\infty \frac{F_2(r)}{V_s} \iint_{A_p} L(r_p, r, \Delta\varphi_p)\Phi_p r_p dr_p d\varphi_p r dr d\varphi. \quad \text{(S1)}$$

In Eq. (S1), the first integral extends over the full planar angle, the second integral extends over all radial distances from the primary particle trajectory, and the double integral extends over the area of the annulus, A_p (see Fig. S1). r and φ are polar coordinates of a point in a plane perpendicular to the primary particle trajectory relative to the point of its intersection with this plane. r_p and φ_p are the polar coordinates of this intersection point with respect to the center of the CV. $F_2(r)$ is the complementary cumulative probability of an IC that depends on the radial distance r of a site from the primary particle trajectory. V_s is the volume of the site.

$L(r,r_p,\Delta\varphi_p)$ is the length of a chord through the CV that is parallel to the primary particle trajectory and passes the point described by vector $\boldsymbol{r_s}$. The length of this chord depends on the distance r_s of this point from the CV center, which is a function of r, r_p and the difference $\Delta\varphi_p$ of the azimuth angles φ_p and φ. L has nonzero values only if point $\boldsymbol{r_s}$ is within the cross section of the CV in the plane. (That is, for the points on the arc shown as a solid black line in Fig. S1.)

Finally, Φ_p is the fluence of primary particles given by

$$\Phi_p = \frac{n_t(D)}{A} \quad \text{(S2)}$$

where n_t and A are the mean number of primary particle tracks and the area A considered in Eqs. (1) and (2). (For a single event, n_t has to be replaced by unity.)

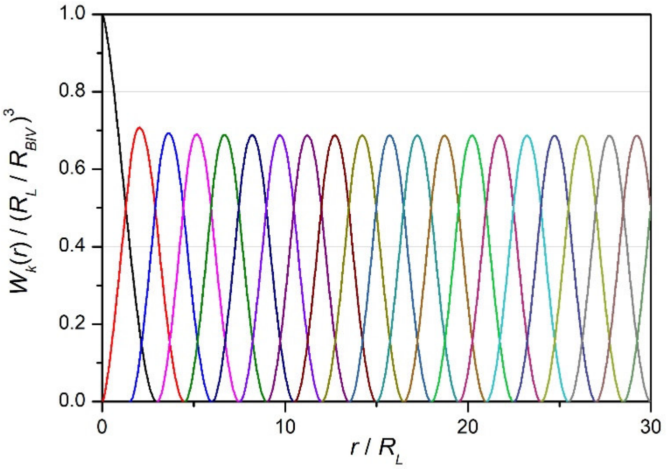

Fig. S2: Weighting function for the radial distribution of ionization clusters around a proton track used in Eq. (14) in the paper. R_{BIV} is the radius of the (spherical) basic interaction volume in which ionization clusters are scored. R_L is the radius of the spherical region of interest. If the region of interest is the BIV, then the outcome of Eq. (S3) is the probability of the formation of an ionization cluster in the BIV when proton tracks traverse the respective annulus around the BIV cross section. If the region of interest is a cluster volume (CV) containing several BIVs, then the result of Eq. (S3) is the expected number of BIVs within the LIV that receive an ionization cluster when a proton track passes the respective annulus.

For a fixed value of φ, only points within the dark-shaded part of the annulus are linked to nonzero chord lengths where the endpoints of the vector ***r*** cover the dark-shaded area within the CV cross section in Fig. S1. Therefore, it is convenient to rewrite Eq. (S1) as follows:

$$\bar{n} = \Phi_p \int_0^\infty F_2(r) \times W(r|R_L, R_-, R_+)\, 2\pi r dr \qquad \text{(S3)}$$

where $W(r|R_L, R_-, R_+)$ is a geometrical weighting function that is given by Eq. (S4).

$$W(r|R_L, R_-, R_+) = \frac{1}{V_S} \iint_{A_p} L(r_p, r, \Delta\varphi_p) r_p dr_p d\varphi_p. \qquad \text{(S4)}$$

In this work, the weighting functions were evaluated for the cases that the radii R_- and R_+ are successive integer multiples of the ROI radius R_L.[1] The respective weighting functions are denoted by the upper integer as subscript, i.e., $W_k(r)$, where $k = 1$ corresponds to a passage of the primary particle trajectory through the CV. $W_1(0)$ is the ratio of the volumes of the ROI and the BIV, i.e., unity if the ROI is identical to the BIV. If the ROI is the CV of Schneider et al. (2020), $W_1(0)$ is the number of BIVs per CV. (Whereas Schneider et al. (2019, 2020) used the ratio of mean chord length through the CV and BIV diameter for the number of BIV per CV that potentially receive an ionization cluster.)

The resulting weighting functions W_k are dimensionless and have domains and co-domains that scale with R_L and the third power of R_L/R_{BIV}, respectively, and are shown for the first 20 annuli in Fig. S2. Except for the case of ROI traversal, the weighting functions of all annuli have the same maxima and functional shape and are only shifted with respect to each other.

Proton tracks simulated in previous work (Rabus et al. 2020) are used as data for a showcase example. In the simulations, protons were started in water with start energies between 1 MeV and 99 MeV at the surface of a slab of water of 650 nm thickness. Positions and energy transfers were recorded for each interaction of the proton or its secondary electrons within this slab. Secondary electrons were tracked until their energy dropped below the ionization threshold of water (about 11 eV). To obtain reasonable statistics, 5×10^4 individual tracks were simulated for each proton energy.

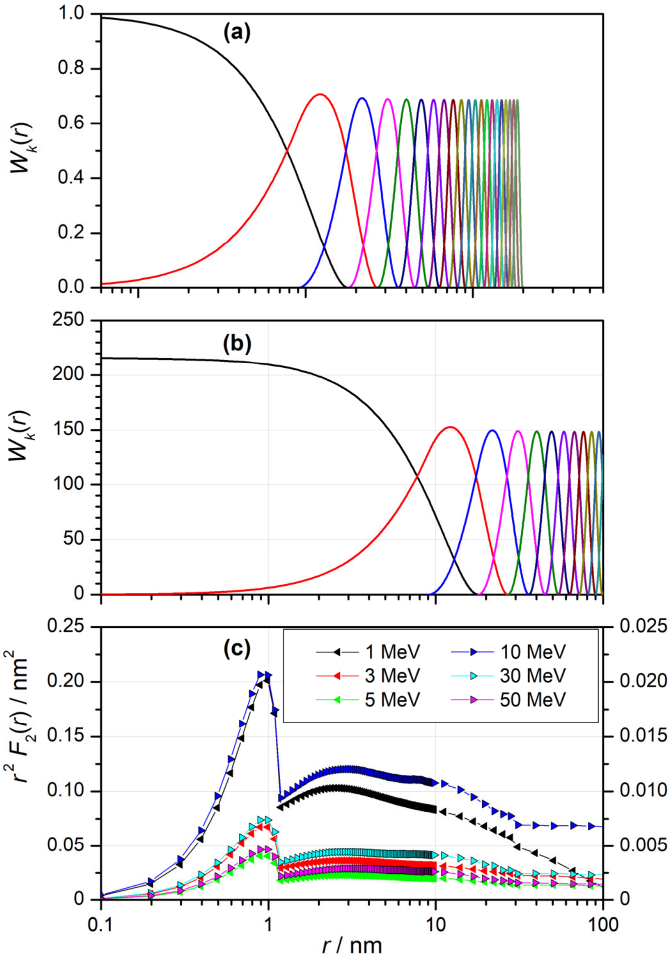

Fig. S3: (a) Weighting function for the radial distribution of ionization clusters considering the contribution of protons passing through one of the first 20 annuli around the cross section of a spherical BIV of 3 nm diameter. (b) Weighting function for the radial distribution of ionization clusters considering the contribution of protons passing through one of the first ten annuli around the cross section of a spherical CV of 18 nm. The weighting functions apply to the condition that the proton trajectory traverses the BIV or CV (black line) or through the *k*-th annulus (inner radius (*k*-1) times the BIV or CV radius, outer radius *k* times this radius). (c) Radial distribution of the probability of inducing a true ionization cluster in a cylindrical target of the same volume as the BIV located around trajectories of protons of different energies (Braunroth et al. 2020). (The right-hand side *y*-axis applies to the data points marked with right-pointing triangles.)

[1] The respective code is listed in Subsection "FORTRAN source code of program Radial_Weight" of Supplement 2.

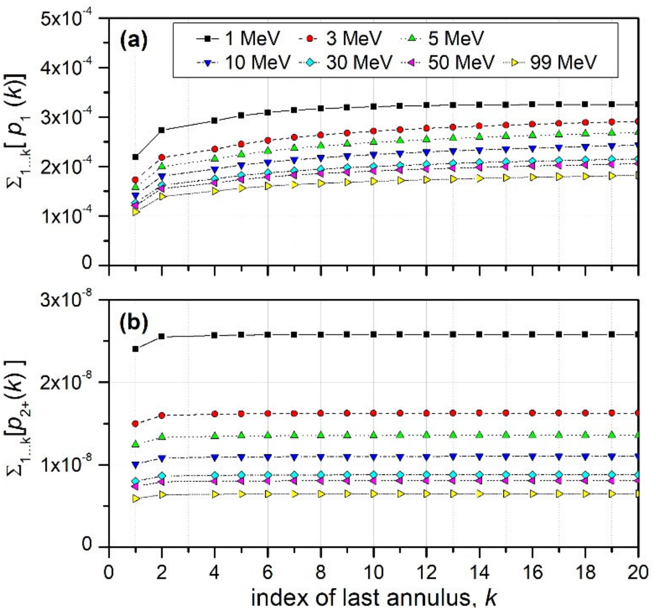

Fig. S4: Probabilities of (a) exactly one and (b) two or more sites in a CV receiving an ionization cluster when a proton passes through one of the first *k* annuli for an absorbed dose of 2 Gy. The data apply to spherical BIVs of 3.0 nm diameter inside a CV of 18 nm diameter.

The proton tracks were analyzed *a posterior* to determine the radial distributions of the frequency of nanometric targets with a certain ionization cluster size, i.e., number of ionizations (Braunroth et al. 2020). The targets were either cylindrical with a diameter of 2.3 nm and a height of 3.4 nm or cylinder shell sectors of Equal volume to the aforementioned cylinders. The cylinder axis was perpendicular to the shortest radial vector from a point on the proton trajectory to the center of the cylinder. Therefore, the frequency of targets with a certain number of ionizations Equals the probability of the formation of such an ionization cluster size when a proton track passes at an impact parameter Equal to the shortest radial distance of the target from the primary particle trajectory.

In the following, it is assumed that the probability of the formation of an ionization cluster in the aforementioned targets is the same as within a spherical target of the same volume (that has a diameter of about 3.0 nm). Ignoring potential changes in the values of F_2 is justified, as the purpose of the discussion given here is to demonstrate the order of magnitude of the effects to be considered.

The weighting functions applying to the case that the ROI is identical to the BIV are shown in Fig. S3(a) for a BIV of 3.0 nm diameter, i.e., of the same volume as the cylindrical targets used by Braunroth et al. (2020). Fig. S3(b) shows the respective weighting functions for the same BIV and a ROI of 18.0 nm diameter that contains the same number of BIVs as the "lethal interaction volumes" reported by Schneider et al. (2019) for protons. The black lines in Fig. S3(a) and Fig. S3(b) refer to a proton traversing the ROI, the red lines to a proton traversing the first annulus, and so forth.

Fig. S3(c) shows the results from Braunroth et al. (2020) for the radial distribution of targets receiving an IC in the tracks of protons of different energies. Triangles pointing left refer to the *y*-axis on the left-hand side and those pointing right to the right-hand side *y*-axis. Owing to the logarithmic *x*-axis, the values were multiplied by r^2 so that the integral under the plot curve is proportional to the contribution of the respective radial interval to the total radial integral. It should be noted that even though there is a pronounced peak for proton tracks passing the target cylinders, a significant proportion of sites with ICs lie at radial distances up to 100 nm and beyond (not shown).

The data presented Fig. S3(a) and Fig. S3(c) have been used to determine the relative contributions to the total probability of induction of an IC in a BIV from protons that pass the BIV within an annulus around the BIV cross section assuming a uniform fluence profile.[2] The respective results are presented in Fig. 3 of the paper.

Using the data shown in Fig. S3(b) and Fig. S3(c) allows the determination of the mean number of sites within a CV that receive an IC from protons passing an annulus around the CV cross section. The results are presented in Fig. 4 of the paper. The respective cumulative contributions of the first *k* annuli are shown in Fig. S4.

[2] The respective code is listed in Subsection "Excel VBA source code of routine convol" of Supplement 2.

Supplementary Figures for Section “Outline of a tentative approach to consider track structure in the TET and RAMN”

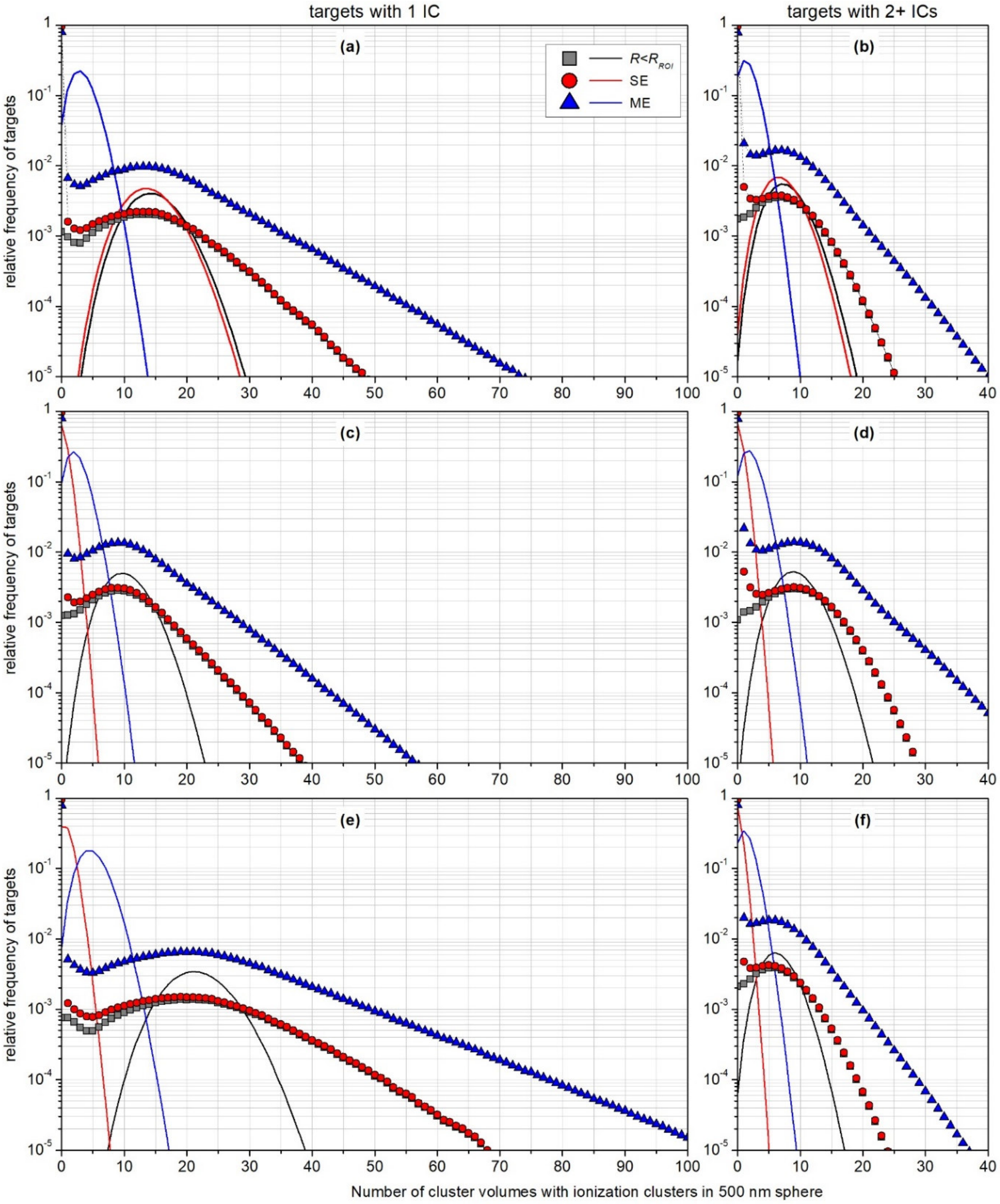

Fig. S5: Frequency distribution of the number of cluster volumes (targets) inside a spherical region of interest (ROI) with radius R_{ROI} = 250 nm that receive a single ionization cluster (IC) (left column), or two or more ionization clusters (right column) induced by protons of 3 MeV energy. Red circles: Single event (SE) distribution, i.e., for a proton track passing the region of interest with a maximum impact parameter of $5R_{ROI}$. Blue triangles: Multi-event (ME) distribution for a dose of 2 Gy. Gray squares: Contribution to the SE distribution coming from proton tracks intersecting the ROI. The ICs are scored in spherical targets of (a) and (b) 2 nm, (c) and (d) 3 nm, and (e) and (f) 2.5 nm diameter. The cluster volumes are spheres of targets of (a) and (b) 12 nm, (c) and (d) 18 nm, and (e) and (f) 7.5 nm diameter. The solid lines indicate Poisson distributions that have the same mean value as the respective corresponding distributions indicated by symbols.

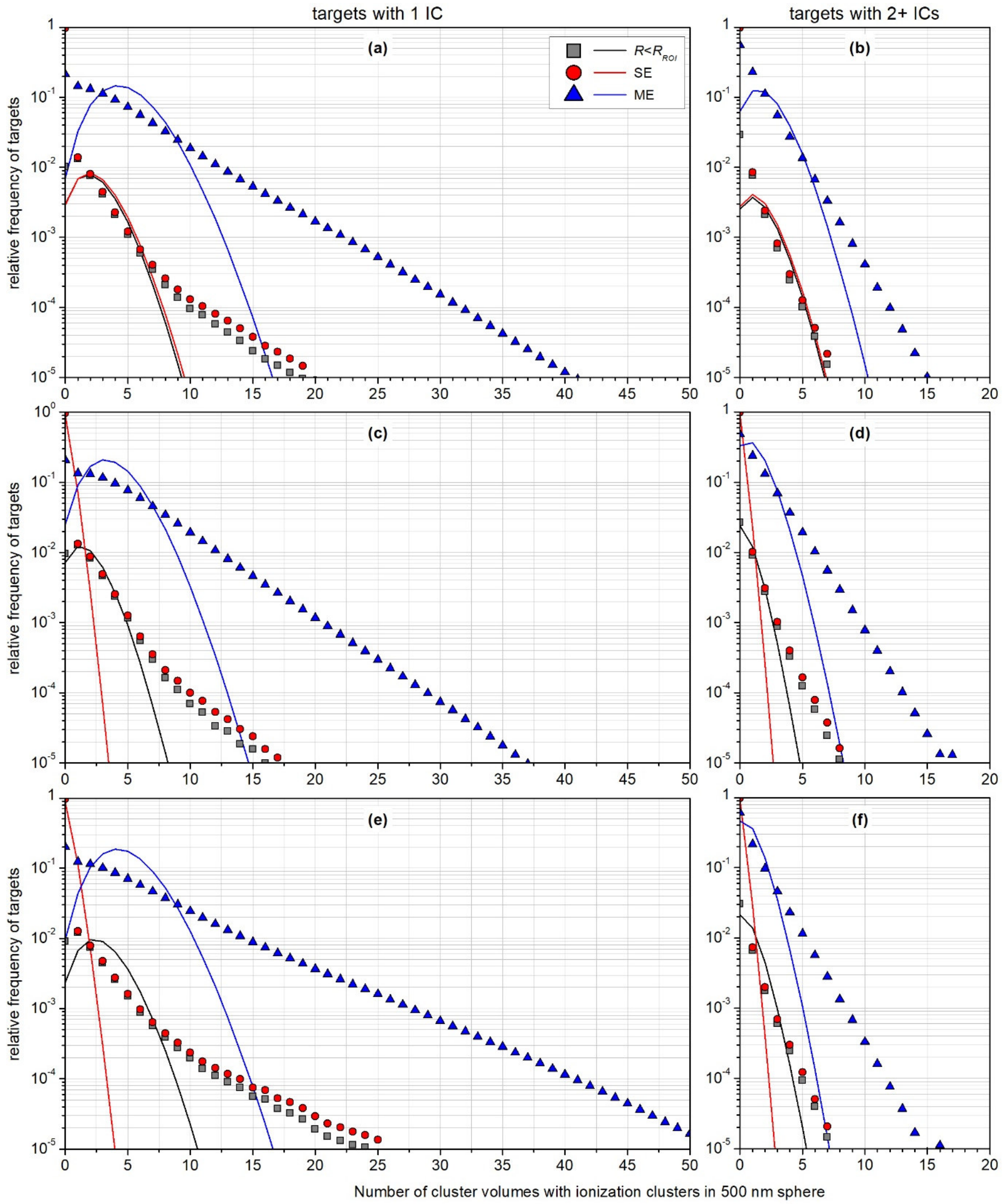

Fig. S6: Frequency distribution of the number of cluster volumes (targets) inside a spherical region of interest (ROI) with radius $R_{ROI} = 250$ nm that receive a single ionization cluster (IC) (left column), or two or more ionization clusters (right column) induced by protons of 50 MeV energy. Red circles: Single event (SE) distribution, i.e., for a proton track passing the region of interest with a maximum impact parameter of $5R_{ROI}$. Blue triangles: Multi-event (ME) distribution for a dose of 2 Gy. Gray squares: Contribution to the SE distribution coming from proton tracks intersecting the ROI. The ICs are scored in spherical targets of (a) and (b) 2 nm, (c) and (d) 3 nm, and (e) and (f) 2.5 nm diameter. The cluster volumes are spheres of targets of (a) and (b) 12 nm, (c) and (d) 18 nm, and (e) and (f) 7.5 nm diameter. The solid lines indicate Poisson distributions that have the same mean value as the respective corresponding distributions indicated by symbols.

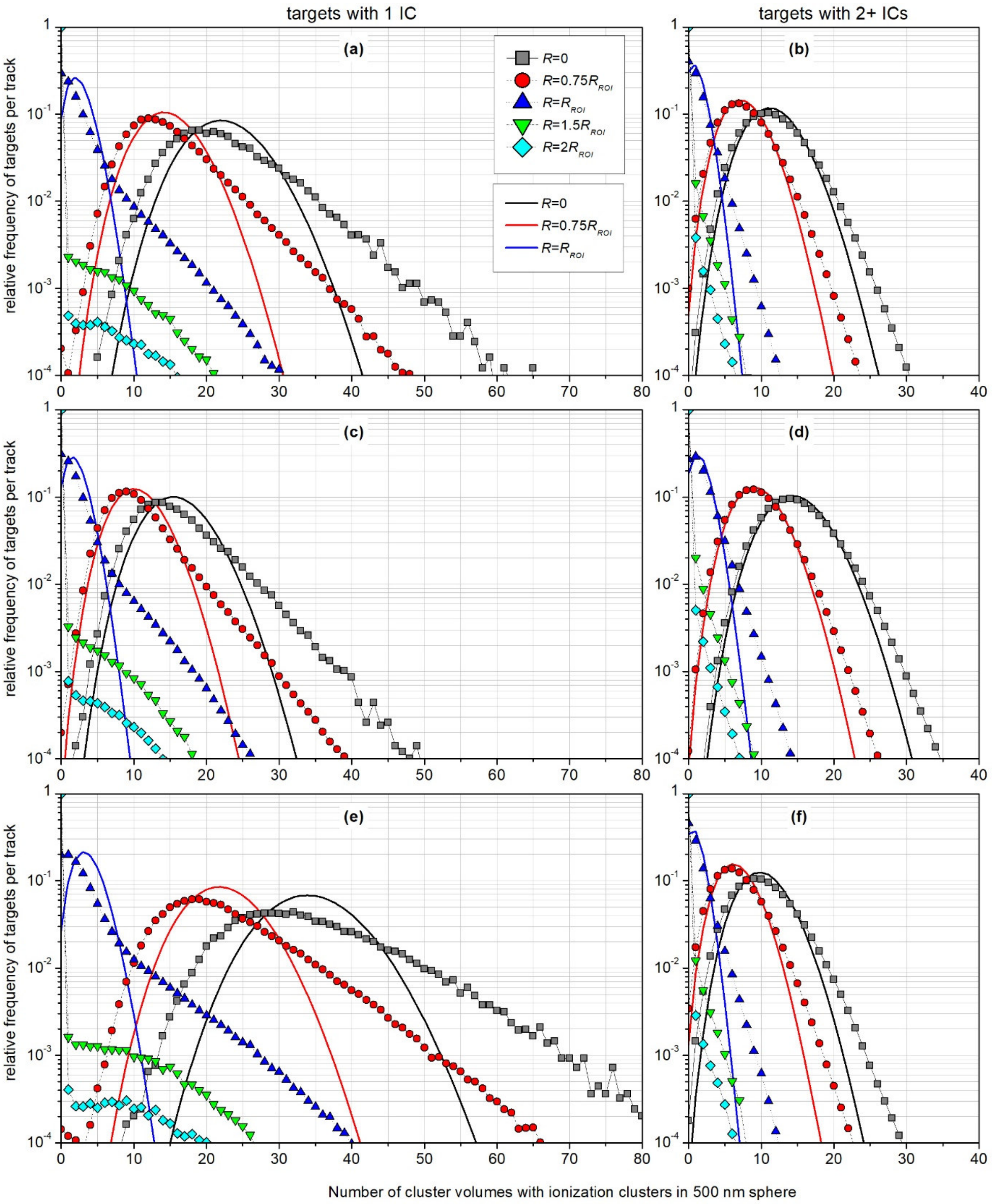

Fig. S7: The symbols indicate the frequency distributions of the number of cluster volumes (targets) inside a spherical region of interest (ROI) with radius R_{ROI} = 250 nm that receive a single ionization cluster (IC) (left column) or two or more ionization clusters (right column) when a proton track (3 MeV proton energy) passes the ROI with an impact parameter R as given in the legend. The ICs are scored in spherical targets of (a) and (b) 2 nm, (c) and (d) 3 nm, and (e) and (f) 2.5 nm diameter. The cluster volumes are spheres of targets of (a) and (b) 12 nm, (c) and (d) 18 nm, and (e) and (f) 7.5 nm diameter. The solid lines indicate Poisson distributions that have the same mean value as the respective corresponding distributions indicated by symbols.

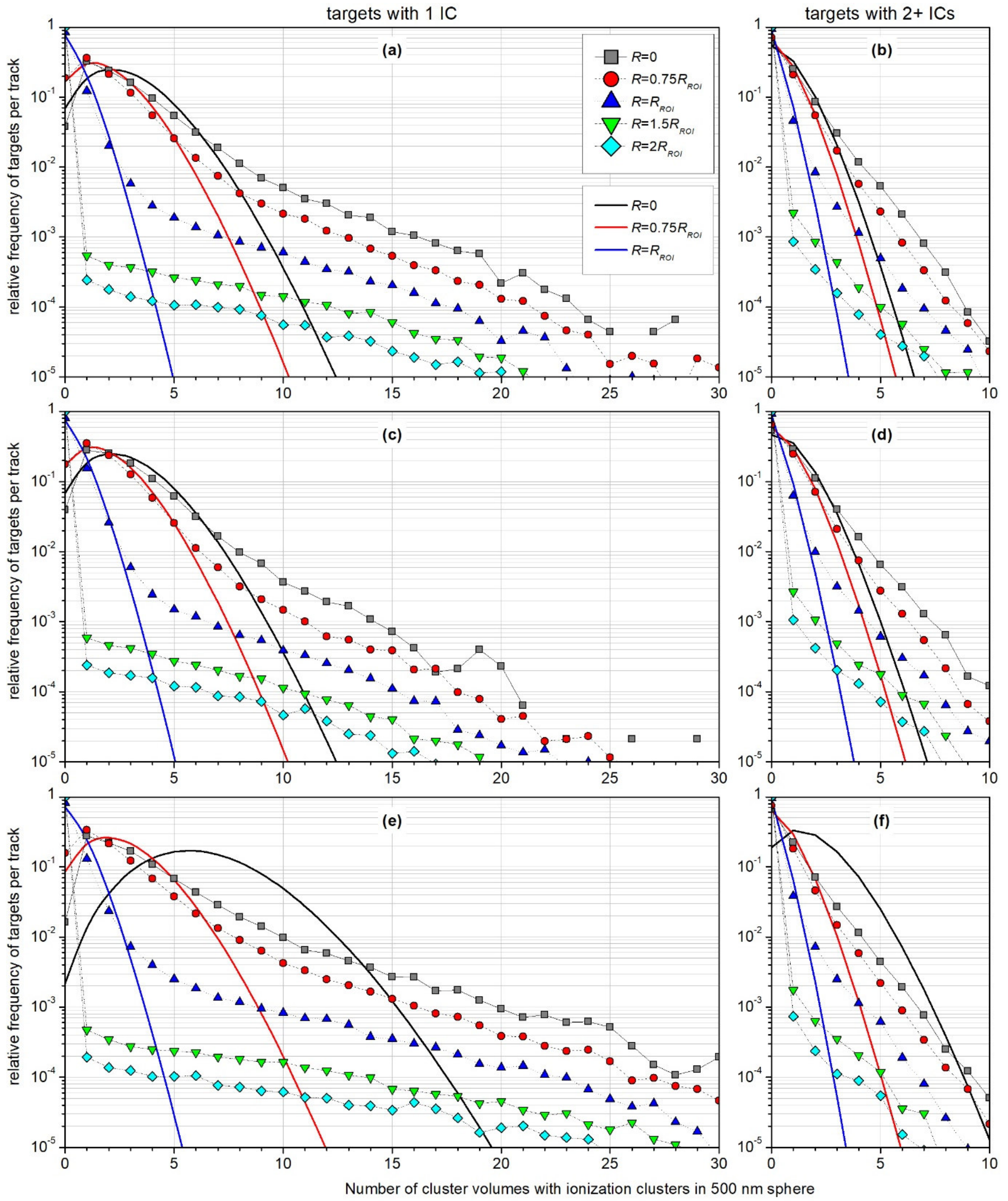

Fig. S8: The symbols indicate the frequency distributions of the number of cluster volumes (targets) inside a spherical region of interest (ROI) with radius R_{ROI} = 250 nm that receive a single ionization cluster (IC) (left column) or two or more ionization clusters (right column) when a proton track (50 MeV proton energy) passes the ROI with an impact parameter *R* as given in the legend. The ICs are scored in spherical targets of (a) and (b) 2 nm, (c) and (d) 3 nm, and (e) and (f) 2.5 nm diameter. The cluster volumes are spheres of targets of (a) and (b) 12 nm, (c) and (d) 18 nm, and (e) and (f) 7.5 nm diameter. The solid lines indicate Poisson distributions that have the same mean value as the respective corresponding distributions indicated by symbols.

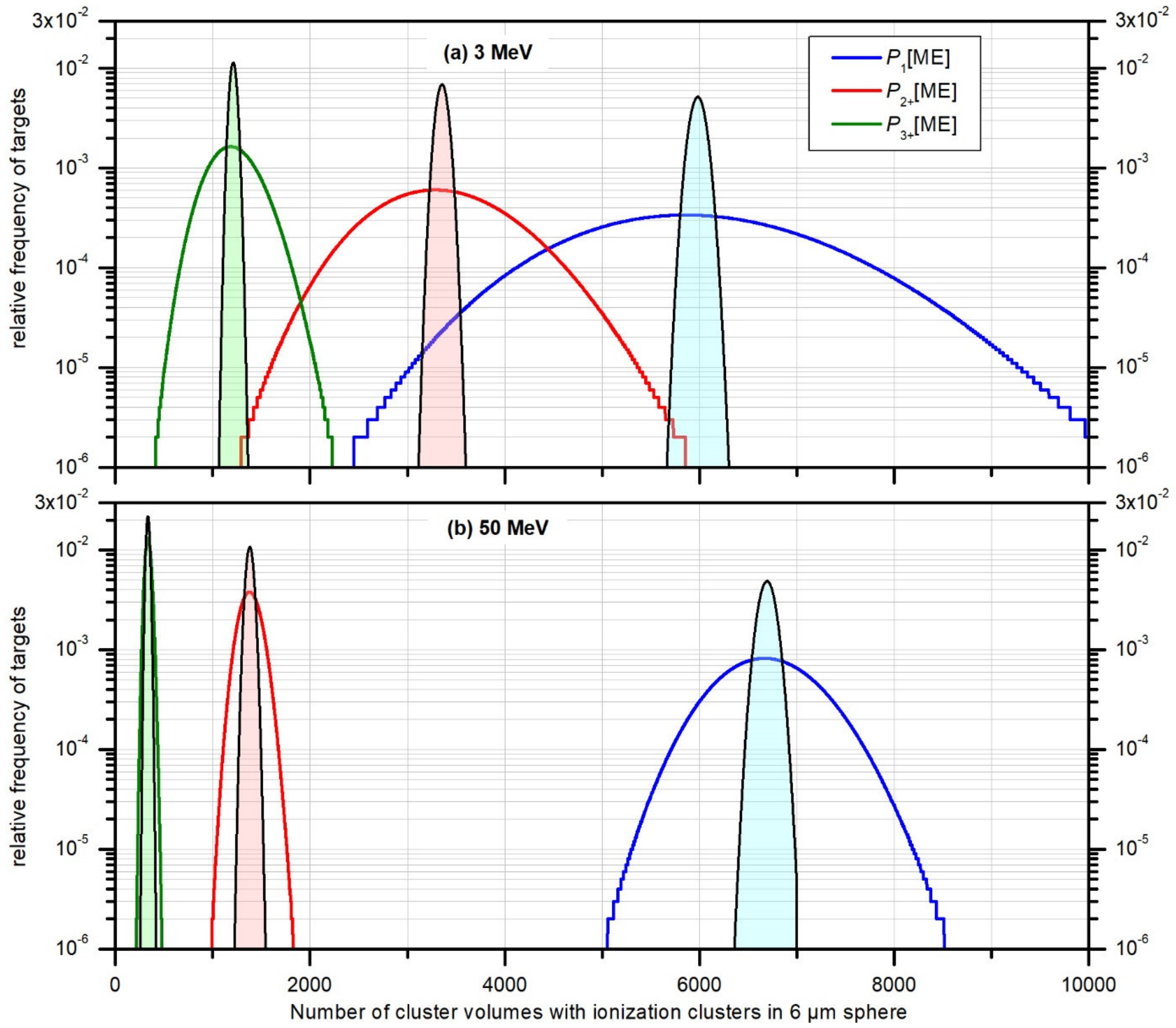

Fig. S9: Frequency distributions of the number of cluster volumes (targets) inside a spherical region of interest (ROI) with radius R_{ROI} = 6 µm that receive a single ionization cluster (blue line), two or more ionization clusters (red line), or three and more ionization clusters (green line) for irradiation with a proton beam of 9.9 µm diameter at a fluence corresponding to an absorbed dose of 2 Gy. The proton energy is (a) 3 MeV and (b) 50 MeV, the ionization clusters are scored in spherical targets of 2 nm diameter, and the considered cluster volumes are spheres of 12 nm diameter as taken from Schneider et al. (2019). The track data used for evaluation have been taken from the work of Alexander et al. (2015).

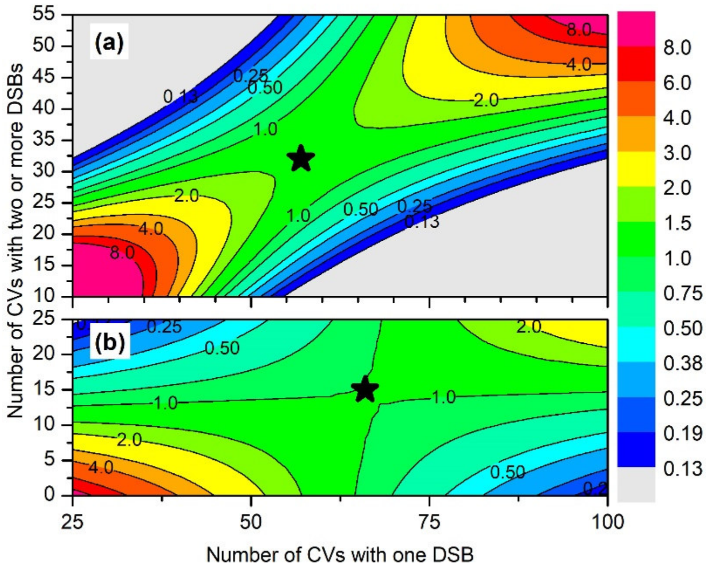

Fig. S10: Bivariate frequency of simultaneous occurrence of a number of cluster volumes (CVs) with one DSB (shown on the *x*-axis) and a number of CVs with two or more DSBs (*y*-axis) normalized to the product of the marginal frequencies. The asterisks mark the location of the modal values of the marginal distributions. The data apply to protons of (a) 3 MeV and (b) 50 MeV energy, an absorbed dose of 2 Gy, and a constant probability of 0.01 for an ionization cluster to be converted to a DSB. The basic interaction volume diameter was 2.0 nm and the cluster volume diameter 12.0 nm as in (Schneider et al. 2019). The track data used for evaluation have been taken from the work of Alexander et al. (2015).

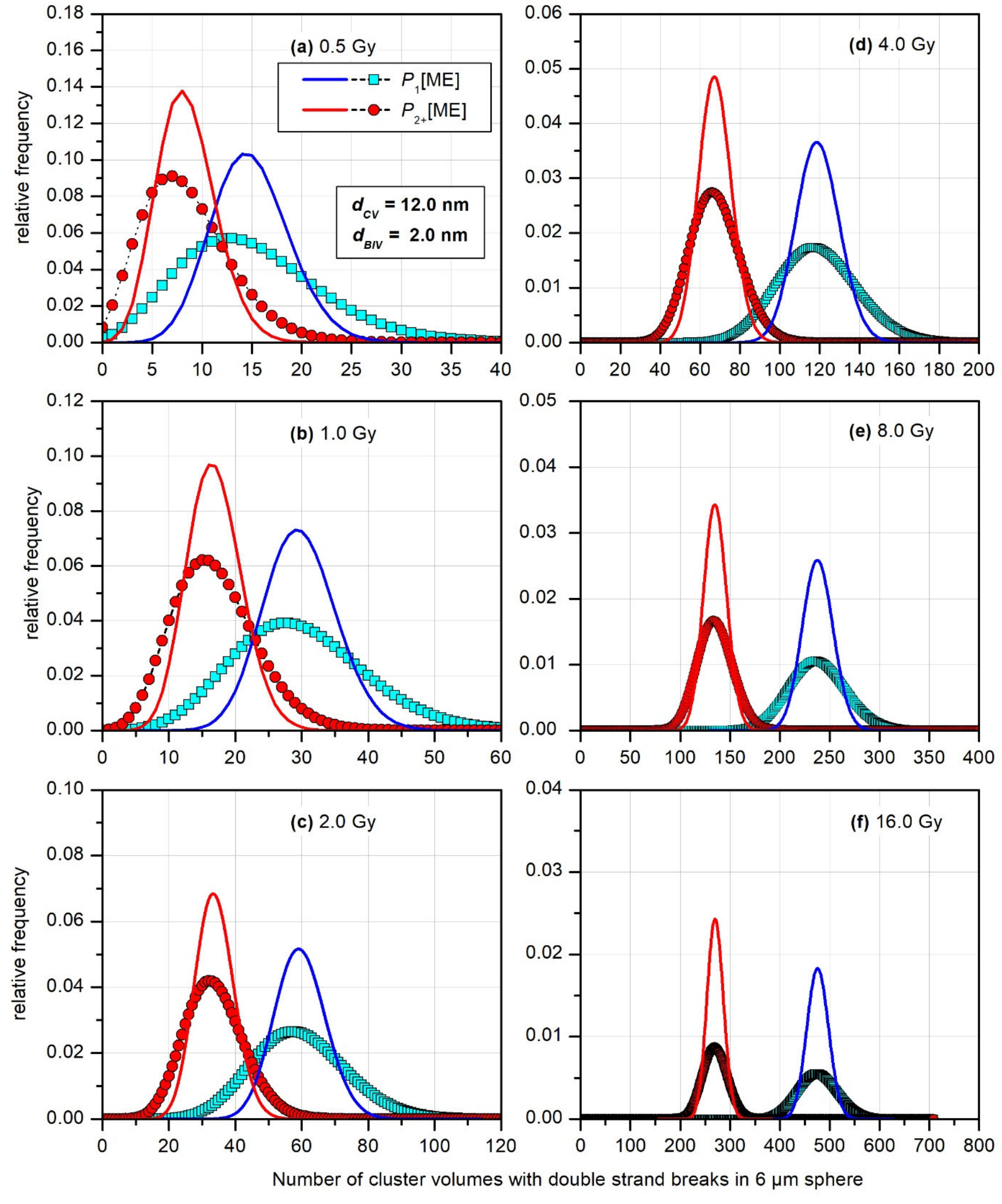

Fig. S11: Frequency distributions of the number of cluster volumes (CVs) inside a spherical region of interest (ROI) with radius R_{ROI} = 6 µm that receive a single ionization cluster (IC) (blue squares) or two or more ICs (red circles) for irradiation with a proton beam of 9.9 µm diameter and 3 MeV energy at different values of absorbed dose: (a) 0.5 Gy, (b) 1 Gy, (c) 2 Gy, (d) 4 Gy, (e) 8 Gy, and (f) 16 Gy. The blue and red solid lines are Poisson distributions of the same mean value as the corresponding data represented by symbols. The results correspond to ICs scored in spherical targets of 2 nm diameter, spherical CVs of 12 nm diameter as taken from (Schneider et al. 2019), and an assumed uniform probability of 0.1% that a CV is filled with DNA. The track data used for evaluation have been taken from the work of Alexander et al. (2015).

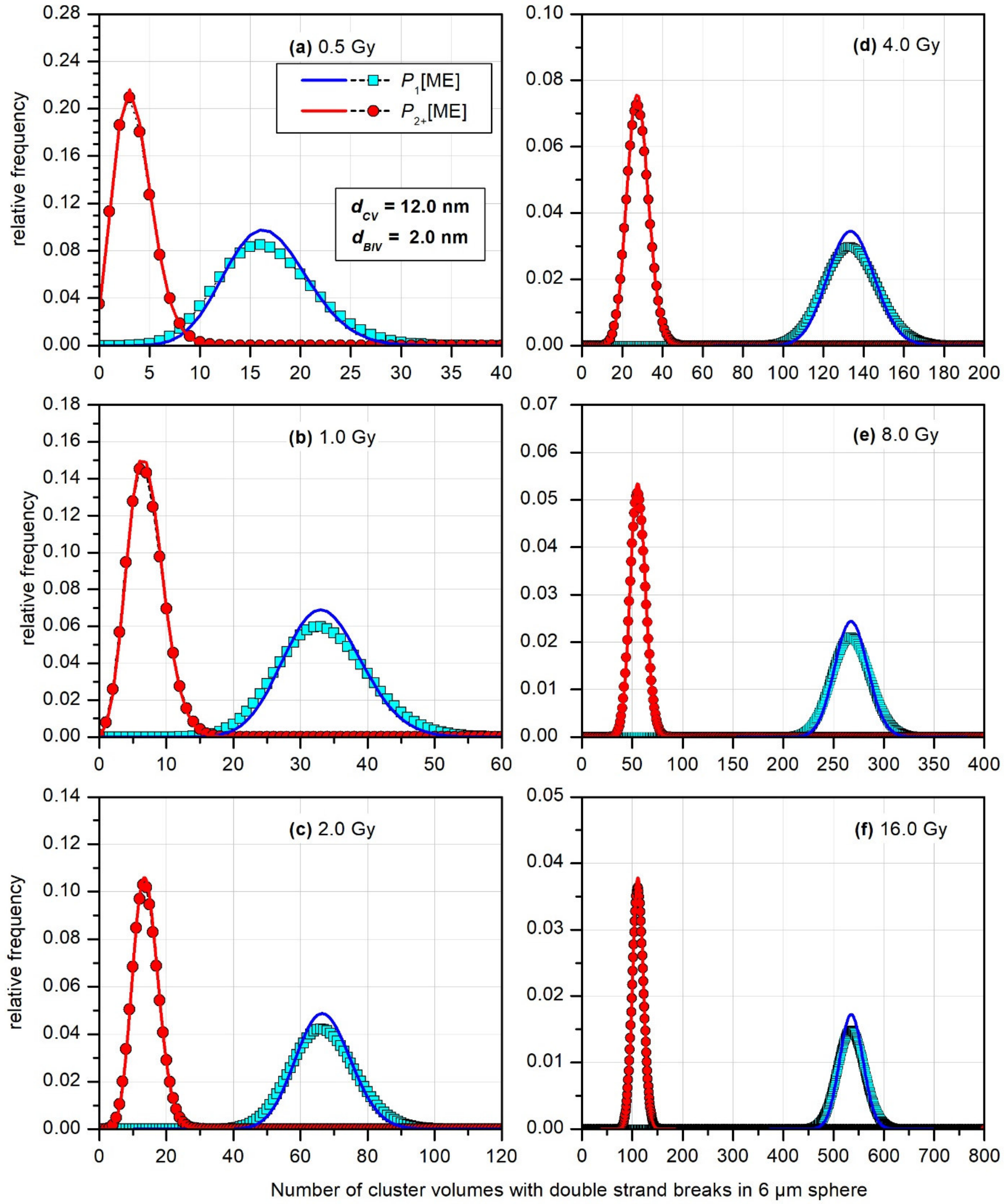

Fig. S12: Frequency distributions of the number of cluster volumes (CVs) inside a spherical region of interest (ROI) with radius R_{ROI} = 6 µm that receive a single ionization cluster (IC) (blue squares) or two or more ICs (red circles) for irradiation with a proton beam of 9.9 µm diameter and 50 MeV energy at different values of absorbed dose: (a) 0.5 Gy, (b) 1 Gy, (c) 2 Gy, (d) 4 Gy, (e) 8 Gy, and (f) 16 Gy. The blue and red solid lines are Poisson distributions of the same mean value as the corresponding data represented by symbols. The results correspond to ICs scored in spherical targets of 2 nm diameter, spherical CVs of 12 nm diameter as taken from (Schneider et al. 2019) and an assumed uniform probability of 0.1% that a CV is filled with DNA. The track data used for evaluation have been taken from the work of Alexander et al. (2015).

References

Alexander F, Villagrasa C, Rabus H, Wilkens J (2015) Energy dependent track structure parametrisations for protons and carbon ions based on nanometric simulations. European Physical Journal D 69216

Braunroth T, Nettelbeck H, Ngcezu S A, Rabus H (2020) Three-dimensional nanodosimetric characterisation of proton track structure. Radiation Physics and Chemistry 176(0):109066

Rabus H, Ngcezu S, Braunroth T, Nettelbeck H (2020) “Broadscale” nanodosimetry: Nanodosimetric track structure quantities increase at distal edge of spread-out proton Bragg peaks. Radiation Physics and Chemistry 166(0):108515

Schneider U, Vasi F, Schmidli K, Besserer J (2019) Track Event Theory: A cell survival and RBE model consistent with nanodosimetry. Radiation Protection Dosimetry 18317-21

Schneider U, Vasi F, Schmidli K, Besserer J (2020) A model of radiation action based on nanodosimetry and the application to ultra-soft X-rays. Radiat Environ Bioph 59(3):1-12
