# Supplementary material for: Investigation into the foundations of the track-event theory of cell survival and the radiation action model based on nanodosimetry
Source: Radiat Environ Biophys. 2021 Aug 24;60(4):559–78. doi: 10.1007/s00411-021-00936-4 (PMC8551112; doi:10.1007/s00411-021-00936-4)
Supplement: Supplementary file 2 — Supplementary file2 (PDF 356 KB) [file 411_2021_936_MOESM2_ESM.pdf]

Supplemental material to “Investigation into the foundations of the track-event theory of cell survival and the radiation action model based on nanodosimetry” in Radiation and Environmental Biophysics

Sonwabile Arthur Ngcezu[1], Hans Rabus[2,*]

[1] *University of the Witwatersrand, Johannesburg, 2000, South Africa*
[2] *Physikalisch-Technische Bundesanstalt (PTB), 10587 Berlin, Germany*
* email: hans.rabus@ptb.de

Source codes of the programs used in this study

FORTRAN source code of program Radial_Weight

This program calculates the weighting factors used in Subsection "Probability of inducing an IC in a BIV by proton tracks" to get from the cumulative nanodosimetric ionization cluster frequencies F_k as a function of radial distance r from the primary particle trajectory the expected (mean) number of targets within a sphere for the cases that the primary particle trajectory intersects the sphere or that it is within cylinder shells whose inner and outer radii are subsequent integer multiples of the sphere radius.

----- FORTRAN source code of program Radial_Weight -----

```
      PROGRAM Radial_Weight
C!>>> Declarations >>>>>>>>>>>>>>>>>>>>>>>>>>>>>>>>>>>>>>>>>>>>>>>>>
      IMPLICIT NONE
C!    ----- Parameters: Version Date
      CHARACTER VDATE*11
      PARAMETER(VDATE='12-MAR-2021')
C
C!    PURPOSE: Calculates the weighting factors that are needed to get
C!    from the cumulative nanodosimetric ionization cluster frequencies
C!    F_k as a function of radial distance r from the primary particle
C!    trajectory the expected (mean) number of targets within a sphere
C!    for the cases that the primary particle trajectory intersects the
C!    the sphere or that it is within cylinder shells whose inner and
C!    outer radii are subsequent integer multiples of the sphere radius.
C
C!    ----- Parameters: General purpose constants
      INTEGER*4 LUN
      REAL*8 ONE, PI, ZERO
      PARAMETER(LUN=11, ONE=1.0, PI=3.1415926, ZERO=0.0)
C!    ----- Parameters for #####
      INTEGER*4 MCYLSH, MRSTEP, MXRVAL, MXYCOL
      PARAMETER(MCYLSH=201, MRSTEP=121, MXRVAL=3*MRSTEP+1,
     &          MXYCOL=2*MCYLSH)
C!    ----- Parameters for #####
      REAL*8 DCYLNM, HCYLNM
      PARAMETER(DCYLNM=2.3E0, HCYLNM=3.4E0)
C!    ----- Functions
      REAL*8 RANDY
      EXTERNAL RANDY
C!    ----- Input parameters for geometry
      INTEGER*4 NBATCH, NCYLSH, NRSTEP, NSAMPL
      REAL*8 D_LIV, R_LIV ! Diameter and radius of LIV in nm
      REAL*8 D_BIV ! Diameter and radius of BIV in nm
C!    ----- Local scalars
```

----- FORTRAN source code of program Radial_Weight -----

```
      CHARACTER FILENM*80, HEADER*80
      INTEGER*4 I, IX, IY, IZ, J, K, L, M, N, NRVAL
      REAL*8 DELTAR, POINTS, RSQR, FNORM, X, Y, YY, Z
C!    ----- Local arrays
C!    RSQMAX: Square of outer radius of cylindrical shells
C!    RSQMIN: Square of inner radius of cylindrical shells
C!    RVALUE: Radial coordinate of the inner cylinder shell
C!    SCORE:  Number of data points in intersection of sphere and
C!            cylindrical shells of adjacent multiples of sphere radius
C!    XYOUT:  Auxiliary array for tabulating output
      REAL*8 RSQMAX(MCYLSH), RSQMIN(MCYLSH),
     &       RVALUE(MXRVAL,MCYLSH), SCORE(MXRVAL,MCYLSH), XYOUT(MXYCOL)
C!    ------
      LOGICAL DEBUG
C!<<<<End declarations<<<<<<<<<<<<<<<<<<<<<<<<<<<<<<<<<<<<<<<<<<<<<<<<<

      PRINT*, 'Program Radial_Weight Version: '//VDATE

      IF(1.EQ.1) THEN ! DUMMY block for readability: Initialize 1111111
C       Initialize Random number see sectionds
        IX = 12345
        IY = 23498
        IZ = 76345
        POINTS=ZERO
        DO I=1,MXRVAL
          DO J=1,MCYLSH
            RVALUE(I,J)=ZERO
            SCORE(I,J)=ZERO
          END DO
        END DO
        D_BIV=2.0
      END IF          ! DUMMY block for readability: Initialize 1111111

      IF(2.EQ.2) THEN ! DUMMY block for readability: Read input 2222222

        PRINT*,'Diameter of BIV (D_BIV) in nm'
        READ(*,*) D_BIV
        PRINT*,'D_BIV =',D_BIV,'nm '

        PRINT*,'Diameter of LIV (D_LIV) in nm'
        READ(*,*) D_LIV
        PRINT*,'D_LIV =',D_LIV,'nm'

        PRINT*,'Number of radial shells (<=',MCYLSH,')'
        READ(*,*) NCYLSH
        IF(NCYLSH.GT.MCYLSH) NCYLSH = MCYLSH
        PRINT*,'NCYLSH = ',NCYLSH

        PRINT*,'Number of steps per radius of LIV (<=',MRSTEP,')'
        READ(*,*) NRSTEP
        IF(NRSTEP.GT.MRSTEP) NRSTEP = MRSTEP
        PRINT*,'NRSTEP = ',NRSTEP

        PRINT*,'Number of batches (after which a message is printed)'
        READ(*,*) NBATCH
        PRINT*,'NBATCH = ',NBATCH

        PRINT*,'Number of random samples per batch'
        READ(*,*) NSAMPL
        PRINT*,'NSAMPL = ',NSAMPL
```

----- FORTRAN source code of program Radial_Weight -----

```
      END IF          ! DUMMY block for readability: Read input 2222222

      IF(3.EQ.3) THEN ! DUMMY block for readability: Init arrays 333333
        R_LIV=D_LIV/2.
        NRVAL=NRSTEP*3+1

        DELTAR=ONE/FLOAT(NRSTEP)
        DO J=1,NRVAL
          RVALUE(J,1)=FLOAT(J-1)*DELTAR
        END DO !J=1,NRVAL
        DO I=2,NCYLSH
          DO J=1,NRVAL
            RVALUE(J,I)=FLOAT(I-2)+FLOAT(J-1)*DELTAR
          END DO !J=1,NRVAL
        END DO !I=2,NCYLSH

        DO I=1,NCYLSH
            X=FLOAT(I-1)
            RSQMIN(I)=X*X
            RSQMAX(I)=RSQMIN(I)+2*X+ONE
        END DO !I=1,NCYLSH

      END IF          ! DUMMY block for readability: Init arrays 333333

      IF(4.EQ.4) THEN ! DUMMY block for readability: Main loop 44444444

      DO N=1,NBATCH ! Main Loop
      DO I=1,NSAMPL ! Main Loop
C!      Algorithm: Random sample data point in one octant and check
C!                 whether point is in sphere. Then increase # tries by
C!                 one and check for L-th possible radial distance from
C!                 primary trajectory within the K-th annulus whether
C!                 the point shifted by the radial distance along x axis
C!                 falls within the K-th annulus.
C!                 To reduce number of calls to random number generator
C!                 the value -x is also considered.
        Y=RANDY(IX, IY, IZ)
        YY=Y*Y
        X=RANDY(IX, IY, IZ)
        IF(X*X+YY.LE.ONE) THEN ! Inside sphere cross section
          Z=SQRT(ONE-X*X-YY)
          DO J=1,2 ! exploit symmetric points
            POINTS=POINTS+ONE
            DO K=1,NCYLSH
              DO L=1,NRVAL
                RSQR=(X-RVALUE(L,K))*(X-RVALUE(L,K))+YY
                IF(RSQR.GE.RSQMIN(K).AND.RSQR.LT.RSQMAX(K)) THEN
                  SCORE(L,K)=SCORE(L,K)+Z
                END IF
              END DO ! L=1,NRVAL
            END DO ! K=1,NCYLSH
C!          Flip signs
            X=-X
          END DO ! J=1,2
        END IF !(X*X+Y*Y+.LE.ONE)
C!
      END DO !  I=1,NSAMPL ! Main Loop
      PRINT*, NSAMPL*N,'/',NSAMPL*NBATCH
      END DO !  N=1,NBATCH ! Main Loop

      END IF          ! DUMMY block for readability: Main loop 44444444
```

----- FORTRAN source code of program Radial_Weight -----

```
      PRINT*, 'Estimate for Pi:',2.*POINTS/NSAMPL/NBATCH
      PRINT*, 'Ratio with Pi:',2.*POINTS/NSAMPL/NBATCH/PI

      IF(7.EQ.7) THEN ! DUMMY block for readability: Normalize 77777
C!      Notes:
C!      a) Normalization to number of points within sphere cross-section
C!         for y>0 corresponds to division by area of 1/2 of unit
C!         circle, i.e., pi/2.
C!      b) Multiplying by pi the effectively produces factor 2 needed
C!         to compensate that only y>0 was scored.
C!      c) Lead factor 2 as only half the chord length was scored above
        FNORM=2.*PI/POINTS ! 10-MAR-2021
        DO K=1,NCYLSH
          DO L=1,NRVAL
            RVALUE(L,K)=RVALUE(L,K)*R_LIV
            SCORE(L,K)=SCORE(L,K)*FNORM
          END DO ! L=1,NRVAL
        END DO ! K=1,NCYLSH
      END IF          ! DUMMY block for readability: Normalize 77777

      IF(9.EQ.9) THEN ! DUMMY block for readability: Write output 99999

        OPEN(LUN,FILE='RW_VolumeFraction.dat',STATUS='UNKNOWN')
        WRITE(LUN,*) '"Output from program Radial_Weight Version '
     &               //VDATE//'"'
        WRITE(LUN,*) '"* Weighting factors of annuli for unit sphere *"'
        WRITE(LUN,*) 'D_LIV/nm= ',D_LIV,' D_BIV/nm= ',D_BIV,
     &               ' NSAMPL= ',NSAMPL*NBATCH
        WRITE(LUN,'(1000a26)') ('      X            Y      ',J=1,NCYLSH)
        FNORM=3./4./PI
        DO I=1,NRVAL
          DO J=1,NCYLSH
            XYOUT(2*J-1)=RVALUE(I,J)
            XYOUT(2*J)= SCORE(I,J)*FNORM
          END DO
          WRITE(LUN,'(1000(2f13.6))') (XYOUT(J),J=1,2*NCYLSH)
        END DO
        CLOSE(LUN)

        OPEN(LUN,FILE='RW_BIV_TARGETS.dat',STATUS='UNKNOWN')
        WRITE(LUN,*) '"Output from program Radial_Weight Version '
     &               //VDATE//'"'
        WRITE(LUN,*) '"* Annulus weight for LIV&BIV of Schneider2019 *"'
        WRITE(LUN,*) 'D_LIV/nm= ',D_LIV,' D_BIV/nm= ',D_BIV,
     &               ' NSAMPL= ',NSAMPL*NBATCH
        WRITE(LUN,'(1000a26)') ('      X            Y      ',J=1,NCYLSH)
C!      The factor in the following code line is the product of
C!      a) the scaling factor for the sphere's volume (calculation above
C!         was for unit sphere) R_LIV**3
C!      b) the volume density of targets (1 per BIV volume)
        FNORM=6./PI*(R_LIV/D_BIV)**3
        DO I=1,NRVAL
          DO J=1,NCYLSH
            XYOUT(2*J-1)=RVALUE(I,J)
            XYOUT(2*J)= SCORE(I,J)*FNORM
          END DO
          WRITE(LUN,'(1000(2f13.6))') (XYOUT(J),J=1,2*NCYLSH)
        END DO
        CLOSE(LUN)
```

----- FORTRAN source code of program Radial_Weight -----

```
         OPEN(LUN,FILE='RW_SAN_TARGETS.dat',STATUS='UNKNOWN')
         WRITE(LUN,*) '"Output from program Radial_Weight Version '
     &                //VDATE//'"'
         WRITE(LUN,*) '"* Weighting factors of annuli for rescaled LIVs '
     &                //'& BIVs corresponding to Sonwabile''s data  *"'
         WRITE(LUN,'(4(a,f8.2),a,i10)') 'D_LIV/nm= ',D_LIV,' D_BIV/nm= ',
     &                D_BIV, ' D_cyl/nm= ',DCYLNM,' H_cyl/nm= ',HCYLNM,
     &                ' NSAMPL= ',NSAMPL*NBATCH
C!       Modifications as of 12-MAR-2021:
C!       1. Calculate the ratio of a) diameter of a sphere of same volume
C!          as the cylinder and b) diameter of the BIV
         FNORM=EXP(LOG(1.5*DCYLNM*DCYLNM*HCYLNM)/3.)/D_BIV
C!       2. Rescale LIV and BIV and radial distances
         D_LIV=FNORM*D_LIV
         R_LIV=FNORM*R_LIV
         D_BIV=FNORM*D_BIV
         DO I=1,NRVAL
           DO J=1,NCYLSH
             RVALUE(I,J)=FNORM*RVALUE(I,J)
           END DO
         END DO
C
         WRITE(LUN,'(2(2(a,f8.2),a))') ' D_cyl/nm= ',DCYLNM,
     &                ' H_cyl/nm= ',HCYLNM, ' --> rescaled values: ',
     &                ' D_LIV/nm= ',D_LIV,' D_BIV/nm= ',D_BIV
C!      End modifications 12-MAR-2021
         WRITE(LUN,'(1000a26)') ('      X            Y      ',J=1,NCYLSH)
C!       The factor in the following code line is the product of
C!       a) the scaling factor for the sphere's volume (calculation above
C!          was for unit sphere) R_LIV**3
C!       b) the volume density of targets (1 per cylinder volume)
         FNORM=R_LIV**3/(PI/4.*DCYLNM*DCYLNM*HCYLNM)
         DO I=1,NRVAL
           DO J=1,NCYLSH
             XYOUT(2*J-1)=RVALUE(I,J)
             XYOUT(2*J)= SCORE(I,J)*FNORM
           END DO
           WRITE(LUN,'(1000(2f13.6))') (XYOUT(J),J=1,2*NCYLSH)
         END DO
         CLOSE(LUN)

       END IF          ! DUMMY block for readability: Write output 99999


C
       END ! PROGRAM Radial_Weight
C!______________________________________________________________________


       REAL*8 FUNCTION RANDY(IX, IY, IZ)
C      Random Number Generator from
C      Wichmann, B.A. and I.D. Hill, Algorithm AS 183: An Efficient
C      and Portable Pseudo-Random Number Generator,
C      Applied Statistics, 31, 188-190, 1982.
       INTEGER*4 MX, MY, MZ, NX, NY, NZ
       REAL*4 AX, AY, AZ, ONE
       PARAMETER (MX=171, MY=172, MZ=170, NX=30269, NY=30307, NZ=30323,
     &           AX=30269., AY=30307., AZ=30323., ONE=1.0)
       IX = MOD(MX * IX, NX)
       IY = MOD(MY * IY, NY)
       IZ = MOD(MZ * IZ, NZ)
```

----- FORTRAN source code of program Radial_Weight -----

```
      RANDY = AMOD(FLOAT(IX)/AX + FLOAT(IY)/AY + FLOAT(IZ)/AZ, 1.)
      RETURN
      END ! FUNCTION RANDY
```

Excel VBA source code of routine convol

This routine is used in an Excel Workbook in which worksheet “Trackdata” includes the results from Braunroth et al. (2020) for the radial dependence of parameter F_2, which are multiplied by $2\pi r$ in wotksheet “Tracks”. Worksheet “RR_SAN” contains the output file for the weighting functions for the different annuli calculated with the code listed in Subsection “FORTRAN source code of program Radial_Weight”. The routine calculates the integral on the right-hand side of Eq. (31) in the paper and writes the results into worksheet “Convol”. These results are further processed in additional worksheets to calculate the mean number of ionization clusters (ICs) for a single event or a given value of dose, from which the the probabilities for cluster volumes with a single IC or more than one IC are calculated from Poisson statistics.

----- Excel VBA source code of routine convol -----

```
Sub convol()

Dim Weight As Range, Track As Range, Ziel As Range
Dim EPrim As Integer, Ringe As Integer, Zeilen As Integer, I As Integer,
J As Integer, K As Integer, L As Integer
Dim Count As Double
'
  I = 1
  Set Weight = Worksheets("RR_SAN").Cells(6, 2)
  dx = Weight.Cells(2, 1).Value

  Set Track = Worksheets("Tracks").Cells(3, 1)
  Set Ziel = Worksheets("Convol").Cells(1, 1)
  EPrim = Application.WorksheetFunction.Count(Track.EntireRow) / 2
  Ringe = Application.WorksheetFunction.Count(Weight.EntireRow) / 2
  Zeilen = Application.WorksheetFunction.Count(Weight.EntireColumn)
  Application.ScreenUpdating = False
  Application.Calculation = xlCalculationManual

  For I = 1 To EPrim
    Application.StatusBar = I & "/" & EPrim
    Ziel.Cells(1, I + 1).Value = Track.Offset(-2, 2 * I - 1).Value
    For J = 1 To Ringe
      Ziel.Cells(J + 1, 1).Value = J - 1
      Count = Weight.Cells(1, 2 * J).Value * Track.Cells(1, 2 * I).Value
      For K = 1 To Zeilen
        X = Weight.Cells(K, 2 * J - 1).Value
        L = 2
        While Track.Cells(L, 2 * I - 1).Value < X
          L = L + 1
        Wend
        XL = Track.Cells(L - 1, 2 * I - 1).Value
        XH = Track.Cells(L, 2 * I - 1).Value
        YL = Track.Cells(L - 1, 2 * I).Value
        YH = Track.Cells(L, 2 * I).Value
        Y = (X - XL) * (YH - YL) / (XH - XL) + YL
        Count = Count + Y * Weight.Cells(K, 2 * J).Value
      Next K
      Ziel.Cells(J + 1, I + 1).Value = Count * dx

    Next J
  Next I

  Application.ScreenUpdating = True
  Application.Calculation = xlCalculationAutomatic
```

----- Excel VBA source code of routine convol -----
<pre> Application.StatusBar = False<br><br>End Sub</pre>

FORTRAN source code of program IC_3D

This program reads particle tracks from an Ascii file listing the energy transfer points from ionizations in particle tracks and determines ionization clusters and saves the cluster positions (and their complexity) to an output file named IC_*'input_filename'*. It expects an input file in the format (number of event, *z*-position, radial position, azimuth, energy deposited, *x*-position, *y*-position) or (number of event, particle type, process, *x*-position, *y*-position, *z*-position).

Uses subroutines CLUSTR and BLINIT (included from file BLINIT.f, see section "FORTRAN subroutine BLINIT") and expects input of the name of a file listing the debugging options (example see section "Sample debug options file for use with programs IC_3D and ROI_3D").

Notes:

- **The program must be executed in the directory where the data files are located**.
- The program expects input of the name of a file listing the debugging options (example see section "Sample debug options file for use with programs IC_3D and ROI_3D").
- Inputs of parameters and input file names are prompted for unless they are entered via a text file. (Manual input is initiated by entering 0 with the first prompt.) A sample input file is listed in the table below.

----- Sample input file for IC_3D -----

```
210605.16   ! Command file version number ...
IC_3D       ! ... for this program
DEBUG.opt   ! Debug options file name
2           ! DBIV in nm
0.          ! ZROI(1) Start of region of interest
10000.      ! ZROI(2) End of region of interest
TZ%%%XY     ! CODE for input line structure
4           ! NHEADL Number of Header lines
5           ! NFILES
SPRE_p100MeVA_mai2015.dat
SPRE_p50MeVA_mai2015.dat
SPRE_p10MeVA_mai2015.dat
SPRE_p25MeVA_mai2015.dat
SPRE_p3MeVA_mai2015.dat
```

Meaning of the input lines:

(1) Version date and time of the input file structure in format YYMMDD.HHMM
(2) Name of the program
(3) Name of the file listing the debug options (see section "Sample debug options file for use with programs IC_3D and ROI_3D")
(4) Diameter of the target spheres for scoring ionization clusters (basic interaction volume of (Schneider et al. 2019, 2020))
(5) *z*-coordinate of the start of the region of interest
(6) *z*-coordinate of the end of the region of interest
(7) Encoding of the input file structure. Each letter stand for the meaning of an entry in an input line. T means the number of the primary particle track, X,Y, and Z the x, y, and z coordinates of the transfer point, E the energy deposit (optional). Example: The string 'TZ%%EXY ' indicates that there are seven entries in each line, of which the first is the track number, the second the z coordinate, the fifth the energy, and the sixth and seventh the x and y coordinates.
(8) number of header lines in the input files
(9) number of input files to process
(10) further lines: names of the input files

----- FORTRAN source code of program IC_3D -----

```
      PROGRAM IC_3D
C!>>> Declarations >>>>>>>>>>>>>>>>>>>>>>>>>>>>>>>>>>>>>>>>>>>>>>>>>>
      IMPLICIT NONE
```

----- FORTRAN source code of program IC_3D -----

```
C!    ----- Parameters: Version Date and number
      CHARACTER VDATE*11
      REAL*8 VINPUT  ! Version number for command files YYMMDD.HHMM
C!                   (date and time  file structure was last changed)
C!    #################################################################
      PARAMETER(VDATE='05-JUN-2021',VINPUT=210605.16d0) ! ############
C!    #################################################################
C!    05-JUN-2021 HR:
C!      - Added timestap to output files
C!      - Reworked identification of input file structure
C!      - Added version check for input files
C!    03-JUN-2021 HR:
C!      - Optimized output
C!    02-JUN-2021 HR:
C!      - Fixed soft bug with output of header line
C!      - Fixed problem with formatted output
C!    28-MAY-2021 HR:
C!      - Fixed bug with length of SRDATE variable
C!    23-MAY-2021 HR:
C!      - Modified call to subroutines to get their version date
C!    23-MAY-2021 HR:
C!      - Modified call to subroutines to get their version date
C!    20-MAY-2021 HR:
C!      - Adapted to potential site sizes >= 10 nm
C!    28-MAR-2021 HR:
C!      - Cleaned up unused variables
C!    20-MAR-2021 HR:
C!      - Created this clone of ROI_3D.f for ionization CLUSTR output
C!    ----- Parameters: General purpose constants
      INTEGER*4 LUN, LUNT
      REAL*8 ONE, ZERO
      PARAMETER(LUN=11, LUNT=12, ONE=1.0, ZERO=0.0)
C!    ----- Parameters for lattice orientation
      INTEGER*4 MAZMTH, MDIV, MTHETA, MDIR
      PARAMETER(MAZMTH=1, MDIV=0, MTHETA=2**MDIV,
     &           MDIR=MAZMTH*(MTHETA*(MTHETA+1))/2)
C!    ----- Parameters for track and radial distance histograms
      INTEGER*4 MIONIZ
      PARAMETER(MIONIZ=100000)
C!    ----- Input parameters for geometry
      REAL*8 DLATC  ! Cell lattice constant
      REAL*8 DSITE  ! Diameter of spherical target
C!    ----- Functions
      CHARACTER TSTAMP*24
C!    ----- Local scalars
      CHARACTER FILENM*80, FILOUT*84, HEADER*80, PREFIX*9
      CHARACTER*11 VDATES(2), SRDATE
      INTEGER*4 I, IFILE, IT, ITA, J, K, NAZMTH, NDIV, NFILES,
     &          NFORMT, NHEADL, NTRACS
      INTEGER*4 NIONIZ
      LOGICAL ASKINP
      REAL*8 DUMMY, PIBY4, VCHECK
      REAL*8 ZROI(2)
C!    ----- Local arrays
      REAL*8 RLINE(8)
C!    ----- Global variables
      INTEGER*4 NDIR
      REAL*8 ADJNTB(3,3,MDIR)
      COMMON /LATTICE/ ADJNTB, NDIR
C!    ------
      REAL*8 XYZ(MIONIZ,3)
```

----- FORTRAN source code of program IC_3D -----

```
      COMMON /TRACKS/ XYZ
C!    ------
      LOGICAL DEBUG(4), SRFLAG
      COMMON /VERBOSE/ DEBUG, SRFLAG
C!    ------
      INTEGER*4 ICSIZE(MIONIZ),NSITES
      COMMON /CLSTRS/ ICSIZE, NSITES
C!    ------
C!     CODE: Character string encoding the meaning of the entries in a
C!           line of the input file as follows:
C!           'T'     - number of the primary particle track
C!           'X','Y','Z' - x, y, and z coordinates of the transfer point
C!           'E'     - energy deposit (if applicable)
C!           'I'     - ionization cluster size (if applicable)
C!           '%'     - additional data that are not used
C!           Example: The string 'TZ%%EXY ' indicates that there are 7
C!                    entries in each line, of which the first is the
C!                    track number, the second the z coordinate, the
C!                    fifth the energy, and the sixth and seventh the x
C!                    and y coordinates
C!     IDT: Array of the columns indices of (1-3) x,y,z coordinates
C!                   (4) energy deposit (if present), (5) track number,
C!                   (6) number of ionizations in cluster (if present)
C!                   (7-8) are there for future use
      CHARACTER CODE*8
      INTEGER*2 IDT(8),NDT
      COMMON /RFORMT/IDT,NDT,CODE
C!<<<<End declarations<<<<<<<<<<<<<<<<<<<<<<<<<<<<<<<<<<<<<<<<<<<<<<<<<

      PRINT*, 'Program IC_3D Version: '//VDATE
      SRFLAG=.TRUE. ! Invoke message on Version date from Subroutine
CLUSTR

      IF(1.EQ.1) THEN ! DUMMY block for readability: Read input 1111111
        DEBUG(1)=.FALSE. ! Main program
        DEBUG(2)=.FALSE. ! CLUSTR main sections
        DEBUG(3)=.FALSE. ! CLUSTR IDIR loop
        DEBUG(4)=.FALSE. ! CLUSTR IPOS loop details

        PIBY4=ATAN(ONE)
        PREFIX='IC_0.0nm_'

C!      Check command file version
        PRINT*, 'Enter 0 for manual input or version (YYMMDD.HHMM) '//
     &          'of command file structure '
        READ(*,*) FILENM
        READ(FILENM(1:11),*) VCHECK
        ASKINP=(VCHECK.EQ.ZERO)
        IF(.NOT.ASKINP) THEN
          IF(VCHECK.LT.VINPUT) THEN
            PRINT*, 'Command file structure ',VCHECK,' older than '//
     &              'current version ',VINPUT,'=> STOP.'
            STOP
          END IF
          READ(*,*) FILENM
          IF(FILENM(1:5).NE.'IC_3D') THEN
            PRINT*, 'Command file appears not to be for IC_3D but '//
     &               'for '//FILENM//'=> STOP.'
            STOP
          END IF
        END IF
```

----- FORTRAN source code of program IC_3D -----

```
C!      Read debug options
        IF(ASKINP) PRINT*, 'Enter debug options file name or - for none'
        READ(*,*) FILENM
        OPEN(LUN,FILE=FILENM,STATUS='OLD',ERR=10)
        DO I=1,4
          READ(LUN,*) DEBUG(I)
        END DO ! I=1,4
        CLOSE(LUN)
  10    CONTINUE

C!      Read geometry parameters
        IF(ASKINP) PRINT*, 'Enter site diameter in nm'
        READ(*,*) DSITE
        DLATC=DSITE*SQRT(2.)*EXP(LOG(PIBY4/3)/3.)
                IF(DSITE.LT.10.0d0) THEN
          WRITE(PREFIX(4:6),'(f3.1)') DSITE
        ELSE
          IF(DSITE.LT.100.0d0) THEN
            WRITE(PREFIX(4:6),'(f3.0)') DSITE
          ELSE
            WRITE(PREFIX(4:6),'(i3)') INT(DSITE)
          END IF
        END IF

        IF(ASKINP) PRINT*, 'z position of begin of region of interest'
        READ(*,*) ZROI(1)
        IF(ASKINP) PRINT*, 'z position of end of region of interest'
        READ(*,*) ZROI(2)

        IF(ASKINP) PRINT*, 'Enter 8 character code for data file '//
     &       'structure where ''T'' indicates the track ID,'//
     &       ' ''X'',''Y'' and ''Z'' the respective coordinates, '//
     &       '''E'' the energy deposit (if present) and ''&'' any '//
     &       'other data'
        READ(*,*) CODE
        CALL RFINIT()

        IF(ASKINP) PRINT*, 'Number of header lines'
        READ(*,*) NHEADL

        IF(ASKINP) PRINT*, 'Number of files to process'
        READ(*,*) NFILES
      END IF          ! DUMMY block for readability: Read input 1111111

      IF(2.EQ.2) THEN ! DUMMY block for readability: Initialize 2222222
        NAZMTH=MAZMTH
        NDIV=MDIV
        IF(DEBUG(1)) PRINT*, 'Before CALL BLINIT'
        CALL BLINIT(NAZMTH,NDIV,DLATC,SRDATE) ! Init reziprocal lattice
        VDATES(1)=SRDATE
        IF(DEBUG(1)) PRINT*, 'After CALL BLINIT'
      END IF          ! DUMMY block for readability: Initialize 2222222

      DO IFILE=1,NFILES
        PRINT*, 'Enter file name ',IFILE
        READ(*,*) FILENM

        IF(4.EQ.4) THEN ! DUMMY block for readability: Main Loop  444444
          IF(DEBUG(1)) PRINT*, 'Begin of Block 4'
C!        Init counters
```

----- FORTRAN source code of program IC_3D -----

```
      NIONIZ=1
      NTRACS=1

      IF (NFORMT.EQ.1) THEN
        FILOUT=PREFIX//FILENM(6:80)
      ELSE
        FILOUT=PREFIX//FILENM
      END IF

      OPEN(LUN,FILE=FILENM,STATUS='OLD')
      DO I=1,NHEADL
        READ(LUN,'(a)') HEADER
      END DO
      READ(LUN,*) (RLINE(I),I=1,NDT)
      ITA=NINT(RLINE(IDT(5)))
      DO I=1,3
        XYZ(NIONIZ,I)=RLINE(IDT(I))
      END DO

      NIONIZ=NIONIZ+1

100   CONTINUE ! >>>>>>>>>>>>>>>>>>>>>>>>>>>>>>>>>>>>>>>>>>>>>>>>>>>>
      READ(LUN,*,END=200,ERR=200) (RLINE(I),I=1,NDT)
      IT=NINT(RLINE(IDT(5)))
      DO I=1,3
        XYZ(NIONIZ,I)=RLINE(IDT(I))
      END DO

      IF(IT.EQ.ITA) THEN
        NIONIZ=NIONIZ+1
      ELSE ! Entry from a new track was read
        NIONIZ=NIONIZ-1 ! Decrease to number of ionizations in track
        IF(DEBUG(1)) PRINT*, 'Before CALL CLUSTR'
        IF(MOD(NTRACS,10).EQ.0) PRINT*, NTRACS
        CALL CLUSTR(NIONIZ,ZROI,SRDATE)
        VDATES(2)=SRDATE
        IF(NTRACS.EQ.1) THEN
          OPEN(LUNT,FILE=FILOUT,STATUS='UNKNOWN')
          WRITE(LUNT,'(a)') 'Output from IC_3D.f - Version '//
     &          VDATE//' BLINIT:'//VDATES(1)//' CLUSTR:'//VDATES(2)
     &                //' on '//TSTAMP()
          WRITE(LUNT,'(a,f8.3,a)') 'Ionization cluster positions'//
     &         ' in ion tracks for DSITE=',DSITE,' nm '
          WRITE(LUNT,'(a)') 'Processed input data file: '//FILENM
          WRITE(LUNT,'(a)') 'Track x/nm y/nm z/nm ICS'
        END IF
        DO K=1,NSITES-1
          DO I=K+1,NSITES
            IF(XYZ(K,3).GT.XYZ(I,3)) THEN
              DO J=1, 3
                DUMMY=XYZ(K,J)
                XYZ(K,J)=XYZ(I,J)
                XYZ(I,J)=DUMMY
              END DO ! J=1, 3
            END IF
          END DO ! I=K+1,NSITES
        END DO !  K=1,NSITES-1
        DO K=1,NSITES
          IF(ICSIZE(K).GT.1) THEN
            CALL WRLINE(LUNT,IT,XYZ(K,1),XYZ(K,2),XYZ(K,3),
     &                      ICSIZE(K))
```

----- FORTRAN source code of program IC_3D -----

```
              END IF
            END DO ! K=1,NSITES

            IF(DEBUG(1)) PRINT*, 'After CALL CLUSTR'
            DO J=1,3
              XYZ(1,J)=XYZ(NIONIZ+1,J) ! First ionization in new track
            END DO
            ITA=IT ! Remember new track number
            NTRACS=NTRACS+1
            NIONIZ=2 ! There was already one ionization
          END IF
          GOTO 100 ! <<<<<<<<<<<<<<<<<<<<<<<<<<<<<<<<<<<<<<<<<<<<<<<<<
  200     CONTINUE
            CALL CLUSTR(NIONIZ,ZROI,SRDATE)
            DO K=1,NSITES-1
              DO I=K+1,NSITES
                IF(XYZ(K,3).GT.XYZ(I,3)) THEN
                  DO J=1, 3
                    DUMMY=XYZ(K,J)
                    XYZ(K,J)=XYZ(I,J)
                    XYZ(I,J)=DUMMY
                  END DO ! J=1, 3
                END IF
              END DO ! I=K+1,NSITES
            END DO !  K=1,NSITES-1
            DO K=1,NSITES
              IF(ICSIZE(K).GT.1) THEN
                CALL WRLINE(LUNT,IT,XYZ(K,1),XYZ(K,2),XYZ(K,3),
     &                           ICSIZE(K))
              END IF
            END DO ! K=1,NSITES
            CLOSE(LUNT)
          CLOSE(LUN)
        END IF          ! DUMMY block for readability: Main Loop  4444444

      END DO ! IFILE=1,NFILES

      END PROGRAM ! IC_3D
C!______________________________________________________________________

      INCLUDE 'BLINIT.f'       ! <<<<<<<<<<<<<<<<<<<<<<<<<<<<<<<<<<<<<<
      INCLUDE 'RFINIT.f'
      INCLUDE 'TSTAMP.f'


C!**********************************************************************
      SUBROUTINE CLUSTR(NIONIZ,ZROI,SRDATE)
C!    *****************************************************************
C!    Determine targets with ionization clusters
C!    *****************************************************************
C!>>> Declarations >>>>>>>>>>>>>>>>>>>>>>>>>>>>>>>>>>>>>>>>>>>>>>>>>>>>
      IMPLICIT NONE
C!    ----- Parameters: Version Date
      CHARACTER VDATE*11
      PARAMETER(VDATE='23-MAY-2021')
C!    23-MAY-2021 HR: Added outout of version date to calling program
C!    20-MAY-2021 HR: Fixed severe bug with sorting the hit targets
C!    25-MAR-2021 HR: Removed unneeded variables
C!    20-MAR-2021 HR: Created from ROI_3D_Subs.f
C!    ----- Parameters: General purpose constants
      REAL*8 ONE, ZERO
```

----- FORTRAN source code of program IC_3D -----

```
      PARAMETER(ONE=1.0, ZERO=0.0)
C!    ----- Parameters for lattice orientation
      INTEGER*4 MAZMTH, MDIV, MTHETA, MDIR
      PARAMETER(MAZMTH=1, MDIV=0, MTHETA=2**MDIV,
     &           MDIR=MAZMTH*(MTHETA*(MTHETA+1))/2)
C!    ----- Parameters for track and radial distance histograms
      INTEGER*4 KMAX, MIONIZ, MXRPOS, MXYPOS, MXTARG
      PARAMETER(KMAX=4, MIONIZ=100000, MXRPOS=41, MXTARG=1001,
     &          MXYPOS=4*MXRPOS*(MXRPOS-1)+1)
C!    ----- Local scalars
      INTEGER*4 I, IDIR, IR, J, K
      INTEGER*4 IHOLD, NIONIS
      REAL*8 SAVXYZ, SPROD
C!    ----- Local arrays
      INTEGER*4 ITARGT(MIONIZ,3)
      INTEGER*4 ICSITE(KMAX)
C!           ICSITE is the histogram of absolute frequencies of
C!                  targets with 1, 2, ... >=KMAX ionizations
C!                  occuring for a particular orientation of the
C!                  lattice w.r.t. to the track
C!    ----- Global variables
      INTEGER*4 NDIR
      REAL*8 ADJNTB(3,3,MDIR)
      COMMON /LATTICE/ ADJNTB, NDIR
C!    ------
      REAL*8 XYZ(MIONIZ,3)
      COMMON /TRACKS/ XYZ
C!    ------
      REAL*8 RADIST(MXRPOS),FREQRD(MXTARG,MXRPOS,KMAX)
      COMMON /HISTOG/ RADIST, FREQRD
C!           RADIST is the vector of radial distances
C!           FREQRD initially holds the sum, the sum of squares and the
C!                  sum of variances per track over all tracks.
C!                  In the main program this is converted in the end to
C!                  mean and variance over all analyzed tracks plus the
C!                  the average intra-track variance do to orientation
C!    ------
      LOGICAL DEBUG(4), SRFLAG
      COMMON /VERBOSE/ DEBUG, SRFLAG
C!    ------ Added 14-MAR-2021
      INTEGER*4 ICSIZE(MIONIZ),NSITES
      COMMON /CLSTRS/ ICSIZE, NSITES
C!    ----- Scalars and arrays passed from the calling module
      INTEGER*4 NIONIZ
      REAL*8  ZROI(2)
C!    ----- Scalars and arrays passed to the calling module
      CHARACTER*11 SRDATE
C!<<<<End declarations<<<<<<<<<<<<<<<<<<<<<<<<<<<<<<<<<<<<<<<<<<<<<<<

      SRDATE=VDATE

      IF(SRFLAG) THEN
        PRINT*, 'Subroutine CLUSTR Version: ',VDATE
        SRFLAG=.FALSE.
      END IF
      IF(DEBUG(2)) PRINT*, 'CLUSTR Input',NIONIZ

      DO K=1,KMAX ! Empty local histograms
        ICSITE(K)=0
      END DO ! I=1,KMAX ! Empty local histograms
```

----- FORTRAN source code of program IC_3D -----

```
      IF(DEBUG(2)) PRINT*, 'CLUSTR vor Main Loop NDIR=',NDIR
      IF(DEBUG(2)) PRINT*, 'CLUSTR vor Main Loop NIONIZ=',NIONIZ
      DO IDIR=1,NDIR ! Loop over all orientations
        IF(IDIR.GT.NDIR) GOTO 100
        IF(DEBUG(3)) PRINT*, 'CLUSTR Begin Loop IDIR',IDIR
C!      # Find target volume for all  ionizations in track
        NIONIS=0
        DO I=1,NIONIZ
          IF(XYZ(I,3).GE.ZROI(1).AND.XYZ(I,3).LE.ZROI(2)) THEN
            NIONIS=NIONIS+1
            DO J=1,3
              SPROD= ADJNTB(1,J,IDIR)*XYZ(I,1)
     &              +ADJNTB(2,J,IDIR)*XYZ(I,2)
     &              +ADJNTB(3,J,IDIR)*(XYZ(I,3))
              ITARGT(NIONIS,J)=NINT(SPROD)
            END DO ! J=1,3
          END IF
        END DO ! I=1,NIONIZ

        IF(DEBUG(3)) PRINT*, 'CLUSTR vor sort volume indices',NIONIS
        IF(IDIR.GT.NDIR) STOP
C!      # Sort target volume indices ascending
        DO I=1,NIONIS
          DO J=I+1,NIONIS
            IF(    (ITARGT(I,1).GT.ITARGT(J,1))
     &         .OR.(ITARGT(I,1).EQ.ITARGT(J,1).AND.
     &              ITARGT(I,2).GT.ITARGT(J,2))
     &         .OR.(ITARGT(I,1).EQ.ITARGT(J,1).AND.
     &              ITARGT(I,2).EQ.ITARGT(J,2).AND.
     &              ITARGT(I,3).LE.ITARGT(J,3))) THEN
              DO IR=1,3
                IHOLD=ITARGT(I,IR)
                ITARGT(I,IR)=ITARGT(J,IR)
                ITARGT(J,IR)=IHOLD
                SAVXYZ=XYZ(I,IR)     ! 14-MAR-2021
                XYZ(I,IR)=XYZ(J,IR)  ! 14-MAR-2021
                XYZ(J,IR)=SAVXYZ     ! 14-MAR-2021
              END DO ! IR=1,3
            END IF
          END DO ! J=1,NIONIS
        END DO ! I=1,NIONIS

        IF(DEBUG(3)) PRINT*, 'CLUSTR vor find unique volumes',NIONIS
C!      # Find unique target volumes and score ionizations
        NSITES=1
        ICSIZE(NSITES)=1
        DO I=2,NIONIS
          IF(    ITARGT(I,1).NE.ITARGT(I-1,1)
     &       .OR.ITARGT(I,2).NE.ITARGT(I-1,2)
     &       .OR.ITARGT(I,3).NE.ITARGT(I-1,3)) THEN
            DO J=1,3 ! 14-MAR-2021
              XYZ(NSITES,J)=XYZ(NSITES,J)/REAL(ICSIZE(NSITES))
            END DO
            NSITES=NSITES+1
            ICSIZE(NSITES)=1
            DO J=1,3
              ITARGT(NSITES,J)=ITARGT(I,J)
              XYZ(NSITES,J)=XYZ(I,J)
            END DO ! J=1,3
          ELSE
            ICSIZE(NSITES)=ICSIZE(NSITES)+1
```

----- FORTRAN source code of program IC_3D -----

```
            DO J=1,3 ! 14-MAR-2021
              XYZ(NSITES,J)=XYZ(NSITES,J)+XYZ(I,J)
            END DO
          END IF
        END DO ! I=2,NIONIS

      END DO ! IDIR=1,NDIR ! Loop over all orientations
 100  CONTINUE

      IF(DEBUG(2)) PRINT*, 'CLUSTR vor EXIT'

      END SUBROUTINE CLUSTR
C!______________________________________________________________________

      SUBROUTINE WRLINE(LUN,IT,X,Y,Z,ICS)
      INTEGER*4 ICS,IT,LUN,NCH
      REAL*8 X,Y,Z
      CHARACTER FORMTS*80
      COMMON /FRMT/FORMTS,NCH
      FORMTS='('
      NCH=1
      CALL WRINT(IT)
      CALL WRFLT(X,3)
      CALL WRFLT(Y,3)
      CALL WRFLT(Z,3)
      CALL WRINT(ICS)
      WRITE(FORMTS(NCH:NCH),'(a1)') ')'
      WRITE(LUN,FORMTS) IT,X,Y,Z,ICS
      END SUBROUTINE WRLINE

C!______________________________________________________________________
      SUBROUTINE WRINT(I)
      INTEGER*4 I, LINTEG, NCH, NST
      CHARACTER FORMTS*80, WFORMT*10
      COMMON /FRMT/FORMTS,NCH
      IF(I.EQ.0) THEN
        LINTEG=2
      ELSE
        IF(I.GT.0) THEN
          LINTEG=2+INT(LOG10(REAL(I)))
        ELSE
          LINTEG=3+INT(LOG10(ABS(REAL(I))))
        END IF
      END IF
      NST=NCH+1
      NCH=NCH+3
      WFORMT='(a1,i1,a1)'
      IF(LINTEG.GE.10) THEN
        NCH=NCH+1
        WFORMT='(a1,i2,a1)'
      END IF
      WRITE(FORMTS(NST:NCH),WFORMT) 'i',LINTEG,','
      END

C!______________________________________________________________________
      SUBROUTINE WRFLT(DF,IDIG)
      INTEGER*4 IDIG,NCH,NST
      REAL*8 DF
      CHARACTER FORMTS*80, WFORMT*16
      COMMON /FRMT/FORMTS,NCH
      IF(DF.EQ.0.0) THEN
```

----- FORTRAN source code of program IC_3D -----

```
          LFLOAT=3
        ELSE
          LFLOAT=4+INT(LOG10(ABS(DF)))
          IF(LFLOAT.LT.4) LFLOAT=4
          IF(DF.GT.0) LFLOAT=LFLOAT-1
        END IF
        LFLOAT=LFLOAT+IDIG
        NST=NCH+1
        NCH=NCH+5
        WFORMT='(a1,i1,a1,i1)'
        IF(LFLOAT.GE.10) THEN
          WRITE(WFORMT(6:6),'(i1)') 2
          NCH=NCH+1
        END IF
        IF(IDIG.GT.10) THEN
          WRITE(WFORMT(12:12),'(i1)') 2
          NCH=NCH+1
        END IF
        WRITE(FORMTS(NST:NCH),WFORMT) 'f',LFLOAT,'.',IDIG,','
        END
```

FORTRAN source code of program ROI_3D

This program reads track data (output files of IC_3D or original simulation data) and calculates and outputs the following results:

- Frequency distributions of the number of Wigner-Seitz cells in a (large) spherical region that receive ionization clusters for track at different impact parameters and "true" (infinite radial integral) and conditional (track intersects the spherical target) single event distributions (output file name 3D_*'input_filename'*)
- Bivariate distributions of Wigner Seitz cells containing single or multiple ionization clusters. (Output file name 3B_*'input_filename'*).
- Ratio of bivariate frequency distribution of Wigner Seitz cells containing single or multiple ionization clusters to product of marginal frequencies. (Output file name 3C_*'input_filename'*)

Uses subroutines TARG3D and BLINIT (included from file BLINIT.f, see section "FORTRAN subroutine BLINIT"). Calls subroutine TARG3D after each track has been read and then scores ICs in Wigner Seitz cells.

Notes:

- **The program must be executed in the directory where the data files are located**.
- The program expects input of the name of a file listing the debugging options (example see section "Sample debug options file for use with programs IC_3D and ROI_3D").
- Inputs of parameters and input file names are prompted for unless they are entered via a text file. (Manual input is initiated by entering 0 with the first prompt.) A sample input file is listed in the table below.

----- Sample input file for ROI_3D -----

```
210605.16    ! Command file version number ...
ROI_3D       ! ... for this program
DEBUG.opt    ! Debug options file name
12           ! DLIV in nm
6000.        ! DROI in nm
9900.        ! DBEAM in nm
0    1       ! NTARG(1), NTARG(2) (
5            ! KMAX (if < 2, then maximum allowed is used)
1            ! NZROI Number of regions of interest
5000. 2000.  ! ZROIC(1) and DZROI
TXYZI        ! CODE for input line structure (T=track #, I=ICS)
4            ! NHEADL Number of Header lines
1            ! NFILES
IC_2.0nm_p50MeVA_mai2015.dat
```

Meaning of the input lines:

(1) Version date and time of the input file structure in format YYMMDD.HHMM

----- Sample input file for ROI_3D -----
(2) Name of the program<br>(3) Name of the file listing the debug options (see section "Sample debug options file for use with programs IC_3D and ROI_3D")<br>(4) Diameter of the target sphere within which the clusters are scored (lethal interaction volume of (Schneider et al. 2019) or cluster volume of (Schneider et al. 2020))<br>(5) Diameter of the spherical region of interest within which the target spheres with clusters are scored (e.g. a cell nucleus or a compartment of it).<br>(6) Diameter of the primary beam (circular cross section)<br>(7) Maximum elements of the histograms of frequencies of targets with one cluster or with two and more . If NTARG(1) < 1, then the maximum allowed value is used. If NTARG(2) = 1 then the maximum allowed value is used, if NTARG(2)=0 then correlations are not calculated.<br>(8) Number of clusters for which individual frequencies are scored. (The last element holds the cumulative frequencies of cluster numbers KMAX or higher).<br>(9) Number of regions of interest of size DROI to be scored along the direction of the primary particle track.<br>(10) ZROIC(1) is the *z*-coordinate of the center of the first ROI; DZROI is the increment in ZROI position.<br>(11) Encoding of the input file structure. Each letter stands for the meaning of an entry in an input line. T means the number of the primary particle track, X,Y, and Z the x, y, and z coordinates of the transfer point, E the energy deposit (optional). Example: The string 'TZ%%EXY ' indicates that there are seven entries in each line, of which the first is the track number, the second the z coordinate, the fifth the energy, and the sixth and seventh the x and y coordinates.<br>(12) number of header lines in the input files<br>(13) Number of input files to process<br>(14) further lines: names of the input files

-----
FORTRAN source code of program ROI_3D -----

```
      PROGRAM ROI_3D
C!>>> Declarations >>>>>>>>>>>>>>>>>>>>>>>>>>>>>>>>>>>>>>>>>>>>>>>>>>>
      IMPLICIT NONE
C!    ----- Parameters: Version Date and number
      CHARACTER VDATE*11
      REAL*8 VINPUT  ! Version number for command files YYMMDD.HHMM
C!                    (date and time  file structure was last changed)
C!    ################################################################
      PARAMETER(VDATE='05-JUN-2021',VINPUT=210605.16d0) ! ############
C!    ################################################################
C!    05-JUN-2021 HR:
C!      - Added timestap to output files
C!      - Reworked identification of input file structure
C!    23-MAY-2021 HR:
C!      - Modified call to subroutines to get their version date
C!    17-APR-2021 HR:
C!      - Increased MXTRG2 owing to data set with larger values
C!    13-APR-2021 HR:
C!      - Fixed soft bug: Results were overwritten due to same filename
C!      - Added reduction of output to non-zero values
C!    02-APR-2021 HR:
C!      - Added output of correlation matrix
C!      - Detected and fixed bug in section with convolution
C!    28-MAR-2021 HR:
C!      - Changed normalization of traversing tracks
C!    20-MAR-2021 HR:
C!      - Added input of options file name
C!      - Added discrimination between site size and lattice constant
C!    14-MAR-2021 HR:
C!      - Fixed bug with treatment of last track
C!      - Added common block CLSTRS for info on cluster positions
C!    16-OCT-2020 HR:
C!      - Added readin of geometry parameters via options file
```

FORTRAN source code of program ROI_3D -----

```
C!      - Added readin of multiple file names to process with options
C!      - Added processing of multiple ROIs in track data set
C!    15-MAR-2020 HR:
C!      - Added VDATE and modified COMMON BLOCK VERBOSE
C!      - Added variable NXYPOS to fix bug in TARG3D
C!    ----- Parameters: General purpose constants
      INTEGER*4 LUN
      REAL*8 EPS, ONE, ZERO
      PARAMETER(LUN=11, EPS=1.0e-8, ONE=1.0, ZERO=0.0)
C!    ----- Parameters for lattice orientation
      INTEGER*4 MAZMTH, MDIV, MTHETA, MDIR
      PARAMETER(MAZMTH=1, MDIV=0, MTHETA=2**MDIV,
     &           MDIR=MAZMTH*(MTHETA*(MTHETA+1))/2)
C!    ----- Parameters for track and radial distance histograms
      INTEGER*4 KMAX, MIONIZ, MXRPOS, MXTARG, MXTRG2, MXYPOS, MXZROI
      PARAMETER(KMAX=9, MIONIZ=100000, MXRPOS=101, MXTARG=1025,
     &          MXTRG2=513, MXYPOS=4*MXRPOS*(MXRPOS-1)+1, MXZROI=100)
C!    ----- Input parameters for geometry
      REAL*8 DBEAM  ! Diameter of beam in nm
      REAL*8 DLATC  ! Cell lattice constant
      REAL*8 DROI   ! Diameter of region of interest (cell nucleus size)
      REAL*8 DSITE  ! Diameter of spherical target
      REAL*8 DZROI  ! Increment in position of region of interest
C!    ----- Functions
      CHARACTER TSTAMP*24
C!    ----- Local scalars
      CHARACTER*11 VDATES(2), SRDATE
      CHARACTER FILENM*80, FILOUT*85, HEADER*80, PARAM*2, PREFIX*10
      INTEGER*4 I, IFILE, IT, ITA, J, K, KMX, L, NAZMTH, NDIV, NFILES,
     &          NHEADL, NZROI, NPHI, NTRACS
      INTEGER*4 NIONIZ, NRPOS, NXYPOS
      LOGICAL ASKINP
      REAL*8 COSPHI, DELTAR, DUMMY, FNORM, PIBY4, RROI,
     &       SINPHI, SPOSIN, VCHECK
C!    ----- Local arrays
      INTEGER*4 NTARG(2), NTARGK(KMAX)
      REAL*8 CORRSE(MXTARG,MXTRG2),CONVOL(MXTARG),FREQBV(MXTARG,MXTRG2)
      REAL*8 FREQSE(MXTARG,KMAX),FREQTE(MXTARG,KMAX),ZROIC(MXZROI)
      REAL*8 RLINE(8)
C!    ----- Global variables
      INTEGER*4 NDIR
      REAL*8 ADJNTB(3,3,MDIR)
      COMMON /LATTICE/ ADJNTB, NDIR
C!    ------
      INTEGER*4 IRAD(MXYPOS)
      INTEGER*4 NRROI2 ! Integer of Square of ROI radius
      REAL*8 XROI(MXYPOS), YROI(MXYPOS), ZROI(3)
      COMMON /ROICTR/  XROI, YROI, ZROI, IRAD, NRROI2
C!    ------
      REAL*8 XYZ(MIONIZ,3)
      COMMON /TRACKS/ XYZ
C!    ------
      REAL*8 CORR12(MXTARG,MXTRG2,MXRPOS)
      REAL*8 RADIST(MXRPOS),FREQRD(MXTARG,MXRPOS,KMAX)
C!*      COMMON /HISTOG/ RADIST, FREQRD
      COMMON /HISTOG/ RADIST, FREQRD, CORR12
C!          RADIST is the vector of radial distances
C!          FREQRD initially holds the sum, the sum of squares and the
C!                 sum of variances per track over all tracks.
C!                 In the main program this is converted in the end to
```

FORTRAN source code of program ROI_3D -----

```
C!                    mean and variance over all analyzed tracks plus the
C!                    the average intra-track variance do to orientation
C!    ------
      LOGICAL DEBUG(4), SRFLAG
      COMMON /VERBOSE/ DEBUG, SRFLAG
C!    ------
      INTEGER*4 ICSIZE(MIONIZ),NSITES
      COMMON /CLSTRS/ ICSIZE, NSITES
C!    ------
C!     CODE: Character string encoding the meaning of the entries in a
C!           line of the input file as follows:
C!           'T'     - number of the primary particle track
C!           'X','Y','Z' - x, y, and z coordinates of the transfer point
C!           'E'     - energy deposit (if applicable)
C!           'I'     - ionization cluster size (if applicable)
C!           '%'     - additional data that are not used
C!           Example: The string 'TZ%%EXY ' indicates that there are 7
C!                    entries in each line, of which the first is the
C!                    track number, the second the z coordinate, the
C!                    fifth the energy, and the sixth and seventh the x
C!                    and y coordinates
C!     IDT: Array of the columns indices of (1-3) x,y,z coordinates
C!                   (4) energy deposit (if present), (5) track number,
C!                   (6) number of ionizations in cluster (if present)
C!                   (7-8) are there for future use
      CHARACTER CODE*8
      INTEGER*2 IDT(8),NDT
      COMMON /RFORMT/IDT,NDT,CODE
C!<<<<End declarations<<<<<<<<<<<<<<<<<<<<<<<<<<<<<<<<<<<<<<<<<<<<<<<<<

      PRINT*, 'Program ROI_3D Version: '//VDATE
      SRFLAG=.TRUE. ! Invoke message on Version date from Subroutine
TARG3D

      IF(1.EQ.1) THEN ! DUMMY block for readability: Read input 1111111
        DEBUG(1)=.FALSE. ! Main program
        DEBUG(2)=.FALSE. ! TARG3D main sections
        DEBUG(3)=.FALSE. ! TARG3D IDIR loop
        DEBUG(4)=.FALSE. ! TARG3D IPOS loop details

        PIBY4=ATAN(ONE)
        PREFIX='3D__0.0nm_'
        DO K=1, KMAX
          NTARGK(K)=0
        END DO

C!      Check command file version
        PRINT*, 'Enter 0 for manual input or version (YYMMDD.HHMM) '//
     &          'of command file structure '
        READ(*,*) VCHECK
        ASKINP=(VCHECK.EQ.ZERO)
        IF(.NOT.ASKINP) THEN
          IF(VCHECK.LT.VINPUT) THEN
            PRINT*, 'Command file structure ',VCHECK,' older than '//
     &              'current version ',VINPUT,'=> STOP.'
            STOP
          END IF
          READ(*,*) FILENM
          IF(FILENM(1:6).NE.'ROI_3D') THEN
            PRINT*, 'Command file appear not to be for ROI_3D but '//
```

FORTRAN source code of program ROI_3D -----

```
     &              'for '//FILENM//'=> STOP.'
            STOP
          END IF
        END IF

C!      Read debug options
        IF(ASKINP) PRINT*, 'Enter debug options file name or - for none'
        READ(*,*) FILENM
        OPEN(LUN,FILE=FILENM,STATUS='OLD',ERR=10)
        DO I=1,4
          READ(LUN,*) DEBUG(I)
        END DO ! I=1,4
        CLOSE(LUN)
  10    CONTINUE

C!      Read geometry parameters
        IF(ASKINP) PRINT*, 'Enter site diameter in nm'
        READ(*,*) DSITE
        DLATC=DSITE*SQRT(2.)*EXP(LOG(PIBY4/3.)/3.)
        IF(DSITE.LT.10.0) THEN
          WRITE(PREFIX(5:7),'(f3.1)') DSITE
        ELSE
          WRITE(PREFIX(4:7),'(f4.1)') DSITE
        END IF

        IF(ASKINP) PRINT*, 'Enter ROI diameter in nm'
        READ(*,*) DROI
        RROI=DROI/2.
        NRROI2=INT(RROI*RROI/DLATC/DLATC) ! Note: This is correct as
DLATC is the unit of length
        DELTAR=DROI/40.

        IF(ASKINP) PRINT*, 'Enter beam diameter in nm <= ',5.*DROI
        READ(*,*) DBEAM
        NRPOS=1+NINT(DBEAM/2./DELTAR)
        IF(NRPOS.GT.MXRPOS) THEN
          NRPOS=MXRPOS
          PRINT*, 'Beam diameter ',DBEAM,' nm is too large. <<<<<<<<<<<'
          DBEAM=2.*DELTAR*REAL(MXRPOS-1)
          PRINT*, '>>> maximum possible value ',DBEAM,' is used.'
        END IF

        IF(ASKINP) PRINT*, 'Enter maximum # of targets in histogram'
        READ(*,*) NTARG(1), NTARG(2)
        IF(NTARG(1).GT.MXTARG.OR.NTARG(1).LT.1) NTARG(1)=MXTARG
        IF(NTARG(2).GT.MXTRG2.OR.NTARG(2).EQ.1) NTARG(2)=MXTRG2

        IF(ASKINP) PRINT*, 'Enter maximum ionization cluster '//
     &                     'complexity (KMAX)'
        READ(*,*) KMX
        IF(KMX.GT.KMAX.OR.KMX.LT.2) KMX=KMAX

        IF(ASKINP) PRINT*, 'Enter # of regions of interest along track'
        READ(*,*) NZROI
        IF (NZROI.EQ.1) THEN
          IF(ASKINP) PRINT*, 'Enter z position of region of interest'
          READ(*,*) ZROIC(1)
          DZROI=ZERO
        ELSE
          IF(ASKINP) PRINT*, 'Enter position of first region of '//
```

FORTRAN source code of program ROI_3D -----

```
     &            'interest  (ROI) and increment in ROI position'
          READ(*,*) ZROIC(1), DZROI
          DO I=2,NZROI
            ZROIC(I)=ZROIC(I-1)+DZROI
          END DO
        END IF

        IF(ASKINP) PRINT*, 'Enter 8 character code for data file '//
     &       'structure where ''T'' indicates the track ID,'//
     &       ' ''X'',''Y'' and ''Z'' the respective coordinates, '//
     &       '''E'' the energy deposit (if present) and ''&'' any '//
     &       'other data'
        READ(*,*) CODE
        CALL RFINIT()

        IF(ASKINP) PRINT*, 'Number of header lines'
        READ(*,*) NHEADL

        IF(ASKINP) PRINT*, 'Number of files to process'
        READ(*,*) NFILES

      END IF          ! DUMMY block for readability: Read input 1111111

      IF(2.EQ.2) THEN ! DUMMY block for readability: Initialize 2222222
*         IF(DEBUG(1)) PRINT*, 'Hier v'
        NAZMTH=MAZMTH
        NDIV=MDIV
        IF(DEBUG(1)) PRINT*, 'Hier vor CALL BLINIT'
        CALL BLINIT(NAZMTH,NDIV,DLATC,SRDATE) ! Init reziprocal lattice
        VDATES(1)=SRDATE
        IF(DEBUG(1)) PRINT*, 'Hier nach CALL BLINIT', NRPOS, NTARG

        DO I=1,NRPOS
C!        Define radial offsets of track w.r.t. ROI center
          RADIST(I)=REAL(I-1)*DELTAR
C!        x&y positions of track w.r.t. ROI center (piecake method)
          IF(I.EQ.1) THEN
            NXYPOS=1
            NPHI=0
            IRAD(NXYPOS)=1
            XROI(NXYPOS)=ZERO
            YROI(NXYPOS)=ZERO
          ELSE
            NPHI=NPHI+8
            NXYPOS=NXYPOS+1
            IRAD(NXYPOS)=I
            XROI(NXYPOS)=RADIST(I)
            YROI(NXYPOS)=ZERO
            COSPHI=COS(PIBY4/REAL(I-1))
            SINPHI=SIN(PIBY4/REAL(I-1))
            DO J=2, NPHI
              NXYPOS=NXYPOS+1
              IRAD(NXYPOS)=I
              XROI(NXYPOS)=XROI(NXYPOS-1)*COSPHI-YROI(NXYPOS-1)*SINPHI
              YROI(NXYPOS)=XROI(NXYPOS-1)*SINPHI+YROI(NXYPOS-1)*COSPHI
            END DO
          END IF
        END DO ! DO I=1,NRPOS
      END IF          ! DUMMY block for readability: Initialize 2222222
```

FORTRAN source code of program ROI_3D -----

```
      DO IFILE=1,NFILES
        IF(ASKINP) PRINT*, 'Enter file name ',IFILE
        READ(*,*) FILENM
        IF(.NOT.ASKINP) PRINT*, 'Processing file ',FILENM

        IF(4.EQ.4) THEN ! DUMMY block for readability: Main Loop  444444
          IF(DEBUG(1)) PRINT*, 'Hier beginnt Block 4'
          DO J=1,NRPOS
C!          Init counters
            DO I=1,NTARG(1)
              DO K=1,KMAX
                FREQRD(I,J,K)=ZERO
              END DO
            END DO
C!          Begin 02-APR-2021 >>>>>>>>>>>>>>>>>
            DO I=1,NTARG(1)
              DO L=1,NTARG(2)
                CORR12(I,L,J)=ZERO
              END DO
            END DO
C!          END 02-APR-2021 <<<<<<<<<<<<<<<<<<
          END DO ! DO J=1,NRPOS
          NIONIZ=1
          NTRACS=1

          OPEN(LUN,FILE=FILENM,STATUS='OLD')
          DO I=1,NHEADL
            READ(LUN,*) HEADER
          END DO
   90     READ(LUN,*) (RLINE(I),I=1,NDT)
          IF(IDT(6).GT.0) THEN
            IF(NINT(RLINE(IDT(6))).LT.2) GOTO 90 ! No ionization cluster
          END IF
          ITA=NINT(RLINE(IDT(5)))
          DO I=1,3
            XYZ(NIONIZ,I)=RLINE(IDT(I))
          END DO

          NIONIZ=NIONIZ+1
          IF(DEBUG(1)) PRINT*, 'Hier 4'
  100     CONTINUE ! >>>>>>>>>>>>>>>>>>>>>>>>>>>>>>>>>>>>>>>>>>>>>>>>>>
          READ(LUN,*,END=200,ERR=200) (RLINE(I),I=1,NDT)
          IF(IDT(6).GT.0) THEN
            IF(NINT(RLINE(IDT(6))).LT.2) GOTO 100 ! No ionization cluster
          END IF
          IT=NINT(RLINE(IDT(5)))
          DO I=1,3
            XYZ(NIONIZ,I)=RLINE(IDT(I))
          END DO

          IF(IT.EQ.ITA) THEN
            NIONIZ=NIONIZ+1
          ELSE ! Entry from a new track was read
            NIONIZ=NIONIZ-1 ! Decrease to number of ionizations in track
            IF(DEBUG(1)) PRINT*, 'Hier vor CALL TARG3D'
            IF(MOD(NTRACS,10).EQ.0) PRINT*, NTRACS
            DO J=1,NZROI
              ZROI(2)=ZROIC(J)
              ZROI(1)=ZROI(2)-DROI/2.-DLATC/2.
              ZROI(3)=ZROI(2)+DROI/2.+DLATC/2.
```

FORTRAN source code of program ROI_3D -----

```
            CALL TARG3D(NIONIZ,NRPOS,NTARG,NXYPOS,SRDATE)
          END DO ! J=1,NZROI

          IF(DEBUG(1)) PRINT*, 'Hier nach CALL TARG3D'
          DO J=1,3
            XYZ(1,J)=XYZ(NIONIZ+1,J) ! First ionization in new track
          END DO
          ITA=IT ! Remember new track number
          NTRACS=NTRACS+1
          NIONIZ=2 ! There was already one ionization
        END IF
        GOTO 100 ! <<<<<<<<<<<<<<<<<<<<<<<<<<<<<<<<<<<<<<<<<<<<<<<<<<
  200   CONTINUE
          DO J=1,NZROI
            ZROI(2)=ZROIC(J)
            ZROI(1)=ZROI(2)-DROI/2.-DLATC/2.
            ZROI(3)=ZROI(2)+DROI/2.+DLATC/2.
            CALL TARG3D(NIONIZ,NRPOS,NTARG,NXYPOS,SRDATE)
          END DO ! J=1,NZROI
          VDATES(2)=SRDATE
        CLOSE(LUN)
      END IF          ! DUMMY block for readability: Main Loop  4444444

      IF(6.EQ.6) THEN ! DUMMY block for readability: Normalize  666666
C!      #Normalization
        FNORM=ONE/REAL(NTRACS)
        FNORM=ONE/REAL(NTRACS)/REAL(NZROI)
*        SUMCNT=ZERO
        DO J=1,NRPOS
          DO I=1,NTARG(1)
            DO K=1,KMAX
              FREQRD(I,J,K)=FNORM*FREQRD(I,J,K)
            END DO ! K=1,KMAX
C!          Start 02-APR-2021 new >>>>>>>>>>>>>>>>>>>>
            DO L=1,NTARG(2)
              CORR12(I,L,J)=FNORM*CORR12(I,L,J)
            END DO ! NTARG(2)
C!          End 02-APR-2021 new <<<<<<<<<<<<<<<<<<<<<<<<<
          END DO ! I=1,NTARG(1)
        END DO ! J=1,NRPOS
        DO K=KMX-1,2,-1
          DO J=1,NRPOS
            DO I=1,NTARG(1)
              CONVOL(I)=ZERO
            END DO ! I=1,NTARG(1)
            DO I=1,NTARG(1)
              DO L=0, I-1
                CONVOL(I)=CONVOL(I)+FREQRD(I-L,J,K)*FREQRD(L+1,J,K+1)
              END DO ! L=0, I-1
            END DO ! I=1,NTARG(1)
            DO I=1,NTARG(1)
              FREQRD(I,J,K)=CONVOL(I)
            END DO ! I=1,NTARG(1)
          END DO ! J=1,NRPOS
        END DO ! K=KMX,1,-1
      END IF          ! DUMMY block for readability: Normalize  666666

      IF(8.EQ.8) THEN ! DUMMY block for readability: Prepare Output
8888888
C!      Calculate single-event distributions
```

FORTRAN source code of program ROI_3D -----

```
          DO K=1,KMX
            NTARGK(K)=1
            DO I=1,NTARG(1)
              FREQSE(I,K)=FREQRD(I,1,K)
              SPOSIN=ONE
              DUMMY=ZERO
              DO J=2,NRPOS
                DUMMY=DUMMY+8.
                FREQSE(I,K)=FREQSE(I,K)+DUMMY*FREQRD(I,J,K)
                SPOSIN=SPOSIN+DUMMY
                IF (RADIST(J).LE.RROI) THEN
                  FREQTE(I,K)=FREQSE(I,K)/SPOSIN
                END IF
              END DO
              FREQSE(I,K)=FREQSE(I,K)/SPOSIN
              IF(FREQSE(I,K).GE.EPS.OR.FREQTE(I,K).GE.EPS) NTARGK(K)=I
            END DO
          END DO

          IF(NTARG(2).GT.0) THEN
            IF(NTARGK(2).GT.MXTRG2) THEN
              PRINT*, 'Major problem: 2nd array dimension MXTRG2=',
     &                MXTRG2,' < max. number of 2+ clusters NTARGK(2)=',
     &                NTARGK(2)
              STOP
            END IF
            DO I=1,NTARGK(1)
              DO L=1,NTARGK(2)
                FREQBV(I,L)=CORR12(I,L,1)
                SPOSIN=ONE
                DUMMY=ZERO
                DO J=2,NRPOS
                  DUMMY=DUMMY+8.
                  FREQBV(I,L)=FREQBV(I,L)+DUMMY*CORR12(I,L,J)
                  SPOSIN=SPOSIN+DUMMY
                END DO
                FREQBV(I,L)=FREQBV(I,L)/SPOSIN
                IF(FREQSE(I,1)*FREQSE(L,2).GT.ZERO) THEN
                  CORRSE(I,L)=FREQBV(I,L)/(FREQSE(I,1)*FREQSE(L,2))
                ELSE
                  CORRSE(I,L)=FREQBV(I,L)
                END IF
              END DO
            END DO
          END IF
        END IF          ! DUMMY block for readability: Prepare Output
8888888

       IF(9.EQ.9) THEN ! DUMMY block for readability: OUTPUT  9999999999
C!        #Write results to output file
          WRITE(PREFIX(2:2),'(a)') 'D'
          FILOUT=PREFIX//FILENM
          PRINT*, 'Write output to '//FILOUT
          OPEN(LUN,FILE=FILOUT,STATUS='UNKNOWN')
          WRITE(LUN,*) ' *** Output from PROGRAM ROI_3D Version '//
     &               VDATE//' BLINIT:'//VDATES(1)//' TARG3D:'//VDATES(2)
     &                    //' on '//TSTAMP()
          WRITE(LUN,*) ' Filename: '//FILOUT
          WRITE(LUN,'(a10,10i6)') ' NTARG(K)=',(NTARGK(I),I=1,KMAX)
          WRITE(LUN,'(6(a,f8.3))') ' DLATC=',DLATC,' nm    DROI= ',DROI,
```

FORTRAN source code of program ROI_3D -----

```
     &                            ' nm   DSITE=',DSITE,' nm   DBEAM= ',
     &                            DBEAM,' nm'
          PARAM='P '
          DO K=1,KMX
            IF(K.GT.1) PARAM='F '
            WRITE(PARAM(2:2),'(I1)') K
            WRITE(LUN,'(1X,2a8,103a15)') 'Para-', '#Sites', 'Average',
     &                       'Average',('Distance/nm',J=1,NRPOS)
            WRITE(LUN,'(1X,2a8,2a15,101f15.6)') 'meter', '/track',
     &                      'total','inside',(RADIST(J),J=1,NRPOS)
            DO I=1,NTARGK(K)
              WRITE(LUN,'(1X,a8,i8,103f15.8)') PARAM, I-1,
     &                                  FREQSE(I,K),FREQTE(I,K),
     &                                 (FREQRD(I,J,K),J=1,NRPOS)
            END DO
            WRITE(LUN,*) '____________________________________________'
            WRITE(LUN,*) '********************************************'
          END DO
          CLOSE(LUN)

          IF(NTARG(2).GT.0) THEN ! begin 02-APR-2021 >>>>>>>>>>>>
            WRITE(PREFIX(2:2),'(a)') 'B'
            FILOUT=PREFIX//FILENM
            PRINT*, 'Write output to '//FILOUT
            OPEN(LUN,FILE=FILOUT,STATUS='UNKNOWN')
            WRITE(LUN,*) '*** Output from PROGRAM ROI_3D Version '//
     &              VDATE//' BLINIT:'//VDATES(1)//' TARG3D:'//VDATES(2)
     &                   //' on '//TSTAMP()
            WRITE(LUN,*) 'Filename: '//FILOUT
            WRITE(LUN,'(6(a,f8.3))') ' DLATC=',DLATC,' nm    DROI= ',
     &            DROI,' nm   DSITE=',DSITE,' nm   DBEAM= ',DBEAM,' nm'
            WRITE(LUN,*) 'Correlations P1 and F2'
            WRITE(LUN,*) NTARGK(1),NTARGK(2)
            DO I=1,NTARGK(1)
              WRITE(LUN,'(1X,1000e15.8)') (FREQBV(I,L),L=1,NTARGK(2))
            END DO
            CLOSE(LUN)

            WRITE(PREFIX(2:2),'(a)') 'C'
            FILOUT=PREFIX//FILENM
            PRINT*, 'Write output to '//FILOUT
            OPEN(LUN,FILE=FILOUT,STATUS='UNKNOWN')
            WRITE(LUN,*) '*** Output from PROGRAM ROI_3D Version '//
     &              VDATE//' BLINIT:'//VDATES(1)//' TARG3D:'//VDATES(2)
     &                   //' on '//TSTAMP()
            WRITE(LUN,*) 'Filename: '//FILOUT
            WRITE(LUN,'(6(a,f8.3))') ' DLATC=',DLATC,' nm    DROI= ',
     &            DROI,' nm   DSITE=',DSITE,' nm   DBEAM= ',DBEAM,' nm'
            WRITE(LUN,*) 'Correlations P1 and F2'
            WRITE(LUN,*) NTARGK(1),NTARGK(2)
            DO I=1,NTARGK(1)
              WRITE(LUN,'(1X,1000e15.8)') (CORRSE(I,L),L=1,NTARGK(2))
            END DO
            CLOSE(LUN)
          END IF ! (NTARG(2).GT.0)
        END IF          ! DUMMY block for readability: OUTPUT  999999999

      END DO ! IFILE=1,NFILES

      END PROGRAM ! ROI_3D
```

FORTRAN source code of program ROI_3D -----

```
C!_______________________________________________________________________

      INCLUDE 'BLINIT.f'  ! <<<<<<<<<<<<<<<<<<<<<<<<<<<<<<<<<<<<<<
      INCLUDE 'RFINIT.f'
      INCLUDE 'TSTAMP.f'


C!**********************************************************************
      SUBROUTINE TARG3D(NIONIZ,NRPOS,NTARG,NXYPOS,SRDATE)
C!    ******************************************************************
C!    Determine targets with ionizations
C!    ******************************************************************
C!>>> Declarations >>>>>>>>>>>>>>>>>>>>>>>>>>>>>>>>>>>>>>>>>>>>>>>>>
      IMPLICIT NONE
C!    ----- Parameters: Version Date
      CHARACTER VDATE*11
      PARAMETER(VDATE='23-MAY-2021')
C!    23-MAY-2021 HR: Added outout of version date to calling program
C!    20-MAY-2020 HR: Fixed severe bug with sorting the hit targets
C!    02-APR-2021 HR: Included scoring of correlations
C!    28-MAR-2021 HR: Cleaned up unused variables
C!    15-MAR-2020 HR:
C!      - Added VDATE and modified COMMON BLOCK VERBOSE and added output
of VDATE on first call
C!      - Added input variable NXYPOS to fix bug in processing off-axis
positions
C!      - Fixed bug: Case that no ionizations occur at all was not
properly handled.
C!    14-MAR-2021 HR:
C!      - Added output of cluster positions for analysis of cluster of
clusters
C!    ----- Parameters: General purpose constants
      REAL*8 ONE, ZERO
      PARAMETER(ONE=1.0, ZERO=0.0)
C!    ----- Parameters for lattice orientation
      INTEGER*4 MAZMTH, MDIV, MTHETA, MDIR
      PARAMETER(MAZMTH=1, MDIV=0, MTHETA=2**MDIV,
     &           MDIR=MAZMTH*(MTHETA*(MTHETA+1))/2)
C!    ----- Parameters for track and radial distance histograms
      INTEGER*4 KMAX, MIONIZ, MXRPOS, MXTARG, MXTRG2, MXYPOS
      PARAMETER(KMAX=9, MIONIZ=100000, MXRPOS=101, MXTARG=1025,
     &          MXTRG2=513, MXYPOS=4*MXRPOS*(MXRPOS-1)+1)
C!    ----- Local scalars
      INTEGER*4 I, IDIR, IDIST, IPOS, IR, J, K
      INTEGER*4 IHOLD, NCOUNT, NIONIS
      REAL*8 WEIGHT, SAVXYZ, SPROD
C!    ----- Local arrays
      INTEGER*4 ICROI(3), ITARGT(MIONIZ,3)
      INTEGER*4 ICSITE(KMAX)
      REAL*8 CORREL(MXTARG,MXTRG2,MXRPOS)
      REAL*8 FTGICS(MXTARG,MXRPOS,KMAX)
C!          ICSITE is the histogram of absolute frequencies of
C!                 targets with 1, 2, ... >=KMAX ionizations
C!                 occuring for a particular orientation of the
C!                 lattice w.r.t. to the track
C!          FTGICS initially is the sum of the absolute frequencies
C!                 of targets holding a certain ICS for an orientation
C!                 in the particular track.
C!                 In the end it is normalized and trasferred to global
C!                 counters
```

FORTRAN source code of program ROI_3D -----

```
C!    ----- Global variables
      INTEGER*4 NDIR
      REAL*8 ADJNTB(3,3,MDIR)
      COMMON /LATTICE/ ADJNTB, NDIR
C!    ------
      INTEGER*4 IRAD(MXYPOS)
      INTEGER*4 NRROI2 ! Integer of Square of ROI radius
      REAL*8 XROI(MXYPOS), YROI(MXYPOS), ZROI(3)
      COMMON /ROICTR/  XROI, YROI, ZROI, IRAD, NRROI2
C!    ------
      REAL*8 XYZ(MIONIZ,3)
      COMMON /TRACKS/ XYZ
C!    ------
      REAL*8 CORR12(MXTARG,MXTRG2,MXRPOS)
      REAL*8 RADIST(MXRPOS),FREQRD(MXTARG,MXRPOS,KMAX)
C!*      COMMON /HISTOG/ RADIST, FREQRD
      COMMON /HISTOG/ RADIST, FREQRD, CORR12
C!           RADIST is the vector of radial distances
C!           FREQRD initially holds the sum, the sum of squares and the
C!                  sum of variances per track over all tracks.
C!                  In the main program this is converted in the end to
C!                  mean and variance over all analyzed tracks plus the
C!                  the average intra-track variance do to orientation
C!    ------
      LOGICAL DEBUG(4), SRFLAG
      COMMON /VERBOSE/ DEBUG, SRFLAG
C!    ------
      INTEGER*4 ICSIZE(MIONIZ),NSITES
      COMMON /CLSTRS/ ICSIZE, NSITES
C!    ----- Scalars and arrays passed from the calling module
      INTEGER*4 NIONIZ, NRPOS, NTARG(2), NXYPOS
C!    ----- Scalars and arrays passed to the calling module
      CHARACTER*11 SRDATE
C!<<<<End declarations<<<<<<<<<<<<<<<<<<<<<<<<<<<<<<<<<<<<<<<<<<<<<<

      SRDATE=VDATE

      IF(SRFLAG) THEN
        PRINT*, 'Subroutine TARG3D Version: ',VDATE
        SRFLAG=.FALSE.
      END IF
      IF(DEBUG(2)) PRINT*, 'TARG3D Input',NIONIZ,NRPOS,NTARG,NXYPOS

C!    Init local histograms
      DO K=1,KMAX
        ICSITE(K)=0
        DO J=1,NRPOS
          DO I=1,NTARG(1)
            FTGICS(I,J,K)=ZERO
          END DO ! I=1,NTARG(1)
        END DO ! J=1,NRPOS
      END DO ! I=1,KMAX

C!    Begin 02-APR-2021 >>>>>>>>>
      DO J=1,NRPOS
        DO I=1,NTARG(1)
          DO K=1,NTARG(2)
            CORREL(I,K,J)=ZERO
          END DO ! K=1,NTARG(2)
        END DO ! I=1,NTARG(1)
```

FORTRAN source code of program ROI_3D -----

```
      END DO ! J=1,NRPOS
C!    End 02-APR-2021 <<<<<<<<<<
C!    End init local histograms

      IF(DEBUG(2)) PRINT*, 'TARG3D vor Main Loop NDIR=',NDIR
      IF(DEBUG(2)) PRINT*, 'TARG3D vor Main Loop NIONIZ=',NIONIZ
      DO IDIR=1,NDIR ! Loop over all orientations
        IF(IDIR.GT.NDIR) GOTO 100
        IF(DEBUG(3)) PRINT*, 'TARG3D Begin Loop IDIR',IDIR
C!      # Find target volume for all  ionizations in track
        NIONIS=0
        DO I=1,NIONIZ
          IF(XYZ(I,3).GE.ZROI(1).AND.XYZ(I,3).LE.ZROI(3)) THEN
            NIONIS=NIONIS+1
            DO J=1,3
              SPROD= ADJNTB(1,J,IDIR)*XYZ(I,1)
     &                +ADJNTB(2,J,IDIR)*XYZ(I,2)
     &                +ADJNTB(3,J,IDIR)*(XYZ(I,3)-ZROI(2))
              ITARGT(NIONIS,J)=NINT(SPROD)
            END DO ! J=1,3
          END IF
        END DO ! I=1,NIONIZ

        IF(DEBUG(3)) PRINT*, 'TARG3D vor sort volume indices',NIONIS
        IF(IDIR.GT.NDIR) STOP
C!      # Sort target volume indices ascending
        DO I=1,NIONIS
          DO J=I+1,NIONIS
            IF(    (ITARGT(I,1).GT.ITARGT(J,1))
     &          .OR.(ITARGT(I,1).EQ.ITARGT(J,1).AND.
     &               ITARGT(I,2).GT.ITARGT(J,2))
     &          .OR.(ITARGT(I,1).EQ.ITARGT(J,1).AND.
     &               ITARGT(I,2).EQ.ITARGT(J,2).AND.
     &               ITARGT(I,3).LE.ITARGT(J,3))) THEN
              DO IR=1,3
                IHOLD=ITARGT(I,IR)
                ITARGT(I,IR)=ITARGT(J,IR)
                ITARGT(J,IR)=IHOLD
                SAVXYZ=XYZ(I,IR)
                XYZ(I,IR)=XYZ(J,IR)
                XYZ(J,IR)=SAVXYZ
              END DO ! IR=1,3
            END IF
          END DO ! J=1,NIONIS
        END DO ! I=1,NIONIS

        IF(DEBUG(3)) PRINT*, 'TARG3D vor find unique volumes',NIONIS
C!      # Find unique target volumes and score ionizations
        NSITES=1
        ICSIZE(NSITES)=1
        DO I=2,NIONIS
          IF(    ITARGT(I,1).NE.ITARGT(I-1,1)
     &       .OR.ITARGT(I,2).NE.ITARGT(I-1,2)
     &       .OR.ITARGT(I,3).NE.ITARGT(I-1,3)) THEN
            DO J=1,3 ! 14-MAR-2021
              XYZ(NSITES,J)=XYZ(NSITES,J)/REAL(ICSIZE(NSITES))
            END DO
            NSITES=NSITES+1
            ICSIZE(NSITES)=1
            DO J=1,3
```

FORTRAN source code of program ROI_3D -----

```
              ITARGT(NSITES,J)=ITARGT(I,J)
            END DO ! J=1,3
          ELSE
            ICSIZE(NSITES)=ICSIZE(NSITES)+1
            DO J=1,3
              XYZ(NSITES,J)=XYZ(NSITES,J)+XYZ(I,J)
            END DO
          END IF
        END DO ! I=2,NIONIS

        IF(DEBUG(3)) PRINT*, 'TARG3D Begin Loop IPOS',NXYPOS
        DO IPOS=1,NXYPOS ! Loop over all track positions ! 15-MAR-2020
C!        Calculate cell indices of ROI center
          DO J=1,3
            SPROD= ADJNTB(1,J,IDIR)*XROI(IPOS)
     &                 +ADJNTB(2,J,IDIR)*YROI(IPOS)
*     &                 +ADJNTB(3,J,IDIR)*ZROI(2)
            ICROI(J)=NINT(SPROD)
          END DO ! J=1,3

          IF(DEBUG(4)) PRINT*, 'TARG3D vor Score hit targets',ICROI,
     &                       IPOS, XROI(IPOS)
C!        # Score hit targets
          DO I=1,KMAX ! Zero local counter
            ICSITE(I)=0
          END DO ! I=1,KMAX ! Zero local counter

          DO I=1,NSITES ! Count hit targets
            IDIST=0
            DO J=1,3
              DO K=J,3
                IDIST=IDIST+(ITARGT(I,J)-ICROI(J))
     &                     *(ITARGT(I,K)-ICROI(K))
              END DO
            END DO
            NCOUNT=0
            IF(ABS(IDIST).LE.NRROI2) THEN ! count if inside ROI
              NCOUNT=ICSIZE(I) !  Ionization cluster size
              IF(NCOUNT.GT.KMAX) NCOUNT=KMAX
              ICSITE(NCOUNT)=ICSITE(NCOUNT)+1
*               IF(DEBUG(5)) PRINT*, 'Count hit targets',IDIST,NCOUNT,IPOS
            END IF

          END DO ! I=1,NSITES ! Count hit targets

          IF(DEBUG(3)) PRINT*, 'TARG3D vor add to sum arrays',NDIR,IPOS
C!      # Add this histogram to sum arrays
          IR=IRAD(IPOS)
          IF(DEBUG(3)) PRINT*, 'TARG3D vor add to sum arrays',IR,ICSITE
          DO K=1,KMAX ! Update global counters
            NCOUNT=ICSITE(K)+1
            IF(NCOUNT.GT.NTARG(1)) NCOUNT=NTARG(1)
            IF(NCOUNT.GT.0) FTGICS(NCOUNT,IR,K)=FTGICS(NCOUNT,IR,K)+ONE
          END DO
C!        Begin 02-APR-2021 >>>>>>>>>
          IF(NTARG(2).GT.0) THEN
            ICSITE(1)=ICSITE(1)+1
            IF(ICSITE(1).GT.NTARG(1)) ICSITE(1)=NTARG(1)
            NCOUNT=1
            DO K=2,KMAX
```

----- FORTRAN source code of program ROI_3D -----

```
                NCOUNT=NCOUNT+ICSITE(K)
              END DO
              IF(NCOUNT.GT.NTARG(2)) NCOUNT=NTARG(2)
              CORREL(ICSITE(1),NCOUNT,IR)=
     &                         CORREL(ICSITE(1),NCOUNT,IR)+ONE
            END IF
C!          End 02-APR-2021 <<<<<<<<<<

            IF(DEBUG(3)) PRINT*, 'TARG3D nach sum arrays',NDIR,IDIR
          END DO ! IPOS=1,NXYPOS ! Loop over all track positions
        END DO ! IDIR=1,NDIR ! Loop over all orientations
 100    CONTINUE


C!      # Update global counters
        DO I=1,NRPOS
          IF(I.EQ.1) THEN
            WEIGHT=ONE/REAL(NDIR)
          ELSE
            WEIGHT=ONE/REAL(8*I-8)/REAL(NDIR)
          END IF
          DO J=1,NTARG(1)
            DO K=1,KMAX
              FREQRD(J,I,K)=FREQRD(J,I,K)+WEIGHT*FTGICS(J,I,K)
            END DO  ! K=1,KMAX
C!          Begin 02-APR-2021 >>>>>>>>>
            IF(NTARG(2).GT.0) THEN
              DO K=1,NTARG(2)
                CORR12(J,K,I)=CORR12(J,K,I)+WEIGHT*CORREL(J,K,I)
              END DO ! K=1,NTARG(2)
            END IF ! (NTARG(2).GT.0)
C!          End 02-APR-2021 <<<<<<<<<<
          END DO  ! J=1,NTARG(1)
        END DO ! I=1,NRPOS

        IF(DEBUG(2)) PRINT*, 'TARG3D vor EXIT'

        END SUBROUTINE TARG3D
C!______________________________________________________________________
```

FORTRAN source code of program ME_ROI_3C

This program reads data from output files 3B_*.dat produced by ROI_3D and convolutes them with Binomial distributions of a given success probability such as to convert IC to DSB distributions. Outputs:

- MEA_*'input_filename'* multi- and single event frequency distributions after convolution with binomial compared to frequency distributions of ionization clusters
- MEB_*'input_filename'* bivariate multi-event frequency distribution of single and multiple ionization clusters
- MEC_ *'input_filename'* ratio of bivariate frequency distribution to product of marginal frequencies
- MED_*'input_filename'* multi- and single event frequency distributions after convolution with binomial only (smaller file size)
- SEB_*'input_filename'* bivariate single-event frequency distribution of single and multiple ionization clusters
- SEC_*'input_filename'* ratio of bivariate frequency distribution to product of marginal frequencies

Notes:

- **The program must be executed in the directory where the data files are located**.
- The program expects input of the name of a file listing the debugging options (example see section “Sample debug options file for use with programs IC_3D and ROI_3D”).
- Inputs of parameters and input file names are prompted for unless they are entered via a text file. (Manual input is initiated by entering 0 with the first prompt.) A sample input file is listed in the table below.

----- Sample input file for ME_ROI_3C -----

```
210605.16      ! Command file version number ...
ME_ROI_3C      ! ... for this program
1       ! Flag DEBUG for priniting messages
0.01    ! Relative target density
2.0     ! Absorbed dose in Gy
0    0   ! NMAXME(1), NMAXME(2) (if <= 1 then max allowed is used)
1      ! Number of input files to process
3B_12.0nm_IC_2.0nm_p3MeVA_mai2015.dat
3.
```

Meaning of the input lines:

(1) Version date and time of the input file structure in format YYMMDD.HHMM
(2) Name of the program
(3) Flag for priniting messages on progress (value = 1)
(4) Probability parameter of the Binomial distribution used to convert ionization cluster distributions to DSB cluster distributions.
(5) Absorbed dose in Gy
(6) Maximum dimensions of the matrix of bivariate frequencies of the number of targets with a single DSB and multiple DSBs.
(7) Number of input files to process
(8) further odd lines: names of the input files
(9) further even lines: proton energy in MeV

----- FORTRAN source code of program ME_ROI_3C -----

```
      PROGRAM ME_ROI_3C
C!>>> Declarations >>>>>>>>>>>>>>>>>>>>>>>>>>>>>>>>>>>>>>>>>>>>>>>>>>
      IMPLICIT NONE
C!    ----- Parameters: Version Date and number
      CHARACTER VDATE*11
      REAL*8 VINPUT  ! Version number for command files YYMMDD.HHMM
C!                    (date and time  file structure was last changed)
C!    ################################################################
      PARAMETER(VDATE='05-JUN-2021',VINPUT=210605.1600d0) ! ###########
C!    05-JUN-2021 HR:
C!      - Reorganized command input file.
C!      - Added timestamp in output file
C!    17-APR-2021 HR:
C!      - Cleaned up debug messages
C!    16-APR-2021 HR:
C!      - Adapted to changes done in ROI_3D (leaner output).
C!    13-APR-2021 HR:
C!      - Created
C!    ----- Parameters: General purpose constants
      INTEGER*4 LUN
      REAL*8 ONE, ZERO, PI, WMIN, WMIN2
      PARAMETER(LUN=11, ONE=1.0, ZERO=0.0, PI=3.1415926, WMIN=1d-10,
     &          WMIN2=1d-40)
C!    ----- Parameters for track and radial distance histograms
      INTEGER*4 MXTIC1, MXTIC2, MXTRG1, MXTRG2
      PARAMETER(MXTIC1=1025, MXTIC2=513, MXTRG1=129, MXTRG2=65)
C!    ----- Parameters for fluence calculation from power law
C!          regression to PSTAR data between 1 MeV and 100 MeV
      REAL*8 DPERFL  ! Conversion factor from mass stopping power in
                     ! MeV cm²/g to dose per fluence in Gy nm²
      REAL*8 STPWR1  ! Stopping Power at 1 MeV
      REAL*8 STPEXP  ! Exponent of power law for stopping power
      PARAMETER(DPERFL=1.6e4, STPEXP=-0.7899, STPWR1=276.973)
C!    ----- Functions
```

----- FORTRAN source code of program ME_ROI_3C -----

```
      CHARACTER TSTAMP*24
C!    ----- Local scalars
      CHARACTER FILENM*120, FILOUT*120, HEADER*120
      INTEGER*4 I, I1, I2, IFILE, IJ, J, K, L, NFILES
      LOGICAL ASKINP, DEBUG, HIFLNC
      REAL*8 DBEAM   ! Diameter of beam in nm
      REAL*8 DOSE    ! Absorbed dose in Gray
      REAL*8 DROI    ! Diameter of ROI in nm
      REAL*8 ENERGY  ! Proton energy in MeV
      REAL*8 EVENTS  ! number of events
      REAL*8 FLUENC  ! average number of tracks per ROI cross section
      REAL*8 PVALUE  ! Probability that cluster volume is target
      REAL*8 QVALUE  ! Probability that cluster volume is not a target
      REAL*8 STPWRE  ! Stopping Power at energy E
      REAL*8 DLFLNC, DLWGHT, VCHECK, WGHT
C!    ----- Local arrays
      INTEGER*4 NMAXIC(2), NMAXME(2), NMAXSE(2),
     &          NMCMAX(MXTIC1), NTARGT(8)
      REAL*8 ADEN(2), ANUM(2), APOT(2), PTOK(2), WEIGH(2)
      REAL*8 CORRIC(MXTIC1,MXTIC2)
      REAL*8 PMARG(MXTIC1,8)
      REAL*8 CORRSE(MXTRG1,MXTRG2),CVARSE(MXTRG1,MXTRG2)
      REAL*8 CORRME(MXTRG1,MXTRG2),CVARME(MXTRG1,MXTRG2)
      REAL*8 CTEMP1(MXTRG1,MXTRG2),CTEMP2(MXTRG1,MXTRG2)
      REAL*8 SUMS(8),TGMEAN(8),FNE(MXTRG1,2,2)
C!<<<<End declarations<<<<<<<<<<<<<<<<<<<<<<<<<<<<<<<<<<<<<<<<<<<<<<<<

      PRINT*, 'Program ME_ROI_3C Version: '//VDATE

      IF(1.EQ.1) THEN ! DUMMY block for readability: Read input 1111111
C!    1111111111111111111111111111111111111111111111111111111111111111
C!    Check command file version
      PRINT*, 'Enter 0 for manual input or version (YYMMDD.HHMM) '//
     &          'of command file structure '
      READ(*,*) VCHECK
      ASKINP=(VCHECK.EQ.ZERO)
      IF(.NOT.ASKINP) THEN
        IF(VCHECK.LT.VINPUT) THEN
          PRINT*, 'Command file structure ',VCHECK,' older than '//
     &              'current version ',VINPUT,'=> STOP.'
          STOP
        END IF
        READ(*,*) FILENM
        IF(FILENM(1:9).NE.'ME_ROI_3C') THEN
          PRINT*, 'Command file appear not to be for ROI_3D but '//
     &              'for '//FILENM//'=> STOP.'
          STOP
        END IF
      END IF

      IF(ASKINP) PRINT*, 'Run in debug mode? (1/0)'
      READ(*,*) I
      DEBUG=(I.EQ.1)
      ASKINP=ASKINP.OR.DEBUG

      PRINT*, 'Relative target density (0<PVALUE<1)'
      READ(*,*) PVALUE
      QVALUE=ONE-PVALUE
      IF(ASKINP) PRINT*, 'Absorbed dose in Gy'
      READ(*,*) DOSE
```

----- FORTRAN source code of program ME_ROI_3C -----

```
      IF(ASKINP) PRINT*, 'Size of multi-event bivariate distribution'
      READ(*,*) NMAXME(1), NMAXME(2)
      IF(NMAXME(1).GT.MXTRG1.OR.NMAXME(1).LE.1) NMAXME(1)=MXTRG1
      IF(NMAXME(2).GT.MXTRG2.OR.NMAXME(2).LE.1) NMAXME(2)=MXTRG2

      IF(ASKINP) PRINT*, 'Number of files to process'
      READ(*,*) NFILES
C!    1111111111111111111111111111111111111111111111111111111111111111
      END IF          ! DUMMY block for readability: Read input 1111111

      IF(2.EQ.2) THEN ! DUMMY block for readability: Initialize 2222222
C!    2222222222222222222222222222222222222222222222222222222222222222
C!    2222222222222222222222222222222222222222222222222222222222222222
      END IF          ! DUMMY block for readability: Initialize 2222222

      DO IFILE=1,NFILES
        IF(ASKINP) PRINT*, 'Enter file name ',IFILE
        READ(*,*) FILENM
        IF(ASKINP) PRINT*, 'Enter related proton energy '
        READ(*,*) ENERGY
        STPWRE=STPWR1*EXP(STPEXP*LOG(ENERGY))

        IF(3.EQ.3) THEN ! DUMMY block for readability: Get data  333333
C!      3333333333333333333333333333333333333333333333333333333333333333
        IF(DEBUG) PRINT*, 'Hier beginnt Block 3'

        OPEN(LUN,FILE=FILENM,STATUS='OLD')
        DO I=1,2
          READ(LUN,'(A80)') HEADER
        END DO

        READ(LUN,'(A80)') HEADER
        PRINT*, HEADER
        READ(HEADER(28:36),*) DROI
        READ(HEADER(69:77),*) DBEAM
        FLUENC=0.25*DBEAM*DBEAM*PI*DOSE/DPERFL/STPWRE
        PRINT*, DOSE,ENERGY,STPWRE,FLUENC,DROI,DBEAM

        READ(LUN,'(A)') FILOUT
        READ(LUN,*) NMAXIC(1), NMAXIC(2)
        IF(NMAXIC(1).GT.MXTIC1) THEN
          PRINT*, 'Problem number of lines in '//FILENM//' is ',
     &              NMAXIC(1),' > MXTIC1 =',MXTIC1
          STOP
        END IF
        IF(NMAXIC(2).GT.MXTIC2) THEN
          PRINT*, 'Problem number of columns in '//FILENM//' is ',
     &              NMAXIC(2),' > MXTIC2 =',MXTIC2
          STOP
        END IF

        DO J=1,NMAXIC(1)
          READ(LUN,*) (CORRIC(J,L),L=1,NMAXIC(2))
        END DO

        CLOSE(LUN)
C!      3333333333333333333333333333333333333333333333333333333333333333
        END IF          ! DUMMY block for readability: Get data 3333333

        IF(4.EQ.4) THEN ! DUMMY block for readability: zero arrays 4444
C!      4444444444444444444444444444444444444444444444444444444444444444
```

----- FORTRAN source code of program ME_ROI_3C -----

```
         IF(DEBUG) PRINT*, 'Hier beginnt Block 4 '
         DO I=1,NMAXIC(1)
           NMCMAX(I)=0
           DO J=1,NMAXIC(2)
             IF(CORRIC(I,J).GT.WMIN2) NMCMAX(I)=J
           END DO
         END DO

         DO I=1,MXTIC1
           DO K=1,8
             PMARG(I,K)=ZERO
           END DO
         END DO

         DO I=1,8
           NTARGT(I)=0
         END DO

         DO I=1,MXTRG1
           DO J=1,MXTRG2
             CVARSE(I,J)=ZERO
             CORRSE(I,J)=ZERO
             CVARME(I,J)=ZERO
             CORRME(I,J)=ZERO
             CTEMP1(I,J)=ZERO
             CTEMP2(I,J)=ZERO
           END DO
           DO J=1, 2
             DO K=1,2
               FNE(I,J,K)=ZERO
             END DO
           END DO

         END DO ! DO I=1,NMAXIC(1)
C!       4444444444444444444444444444444444444444444444444444444444444
         END IF          ! DUMMY block for readability: zero arrays 4444

         IF(5.EQ.5) THEN ! DUMMY block for readability: Convolve 5555555
C!       5555555555555555555555555555555555555555555555555555555555555
           IF(DEBUG) PRINT*, 'Hier beginnt Block 5 '
           APOT(1)=ZERO
           PTOK(1)=ONE
           I1=0
           DO I=1,NMAXIC(1)
             IF(I1.LT.NMAXME(1)) I1=I1+1
             APOT(2)=ZERO
             PTOK(2)=ONE
             I2=0
             DO J=1,NMAXIC(2)
               IF(I2.LT.NMAXME(2)) I2=I2+1
               ANUM(1)=APOT(1)
               ADEN(1)=ZERO
               WEIGH(1)=PTOK(1)
               DO K=I,NMAXIC(1)
                 IF(WEIGH(1).GT.WMIN2) THEN
                   ANUM(2)=APOT(2)
                   ADEN(2)=ZERO
                   WEIGH(2)=PTOK(2)*WEIGH(1)
                   DO L=J,NMCMAX(K)
                     IF(WEIGH(2).GT.WMIN2) THEN
                       CVARSE(I1,I2)=CVARSE(I1,I2)+WEIGH(2)*CORRIC(K,L)
```

----- FORTRAN source code of program ME_ROI_3C -----

```
                  ADEN(2)=ADEN(2)+ONE
                  ANUM(2)=ANUM(2)+ONE
                  WEIGH(2)=WEIGH(2)*QVALUE*ANUM(2)/ADEN(2)
                END IF
              END DO ! L=J,NMAXIC(2)
              ADEN(1)=ADEN(1)+ONE
              ANUM(1)=ANUM(1)+ONE
              WEIGH(1)=WEIGH(1)*QVALUE*ANUM(1)/ADEN(1)
            END IF
          END DO ! K=I,NMAXIC(1)
          APOT(2)=APOT(2)+ONE
          PTOK(2)=PTOK(2)*PVALUE
        END DO !J=1,NMAXIC(2)
        APOT(1)=APOT(1)+ONE
        PTOK(1)=PTOK(1)*PVALUE
      END DO !I=1,NMAXIC(1)
C!    5555555555555555555555555555555555555555555555555555555555555555
      END IF          ! DUMMY block for readability: Convolve 555555

      IF(6.EQ.6) THEN ! DUMMY block for readability: Margins 6666666
C!    666666666666666666666666666666666666666666666666666666666666666
      IF(DEBUG) PRINT*, 'Hier beginnt Block 6'
C!      marginal distributions of targets with one IC or multi ICs
      DO I=1,NMAXIC(1)
        DO J=1,NMAXIC(2)
          PMARG(I,5)=PMARG(I,5)+CORRIC(I,J)
          PMARG(J,6)=PMARG(J,6)+CORRIC(I,J)
        END DO !I=1,NMAXIC(1)
      END DO !J=1,NMAXIC(2)
      NTARGT(5)=NMAXIC(1)
      NTARGT(6)=NMAXIC(2)
C!      marginal distributions of targets with one DSB or multi DSBs
      DO I=1,NMAXME(1)
        DO J=1,NMAXME(2)
          PMARG(I,3)=PMARG(I,3)+CVARSE(I,J)
          PMARG(J,4)=PMARG(J,4)+CVARSE(I,J)
          IF(CVARSE(I,J).GT.WMIN2) THEN
            NMAXSE(1)=I
            NMAXSE(2)=J
          END IF
        END DO !I=1,NMAXSE(1)
      END DO !J=1,NMAXSE(2)
      NTARGT(3)=NMAXSE(1)
      NTARGT(4)=NMAXSE(1)
C!      "Relative correlation matrix" - ratio bivariate frequency to
C!      product of marginal frequencies
      DO I=1,NMAXSE(1)
        DO J=1,NMAXSE(2)
          IF(PMARG(I,3).GT.WMIN2.AND.PMARG(J,4).GT.WMIN2) THEN
            CORRSE(I,J)=CVARSE(I,J)/(PMARG(I,3)*PMARG(J,4))
          ELSE
            CORRSE(I,J)=CVARSE(I,J)
          END IF
        END DO !I=1,NMAXSE(1)
      END DO !J=1,NMAXSE(2)
C!    666666666666666666666666666666666666666666666666666666666666666
      END IF          ! DUMMY block for readability: Margins 6666666


      IF(7.EQ.7) THEN ! DUMMY block for readability: Main loop  7777777
C!    7777777777777777777777777777777777777777777777777777777777777777
```

----- FORTRAN source code of program ME_ROI_3C -----

```
      IF(DEBUG) PRINT*, 'Hier beginnt Block 7'
      NTARGT(1)=NMAXSE(1)
      NTARGT(2)=NMAXSE(2)

      PMARG(1,1)=EXP(-FLUENC) ! Kronecker's delta for J=1 (0 targets)
      PMARG(1,2)=EXP(-FLUENC) ! Kronecker's delta for J=1 (0 targets)
      CVARME(1,1)=EXP(-FLUENC)

      EVENTS=ONE
      HIFLNC=(FLUENC.GT.23.4)
      IF(HIFLNC) THEN
        DLFLNC=DLOG(FLUENC)
        DLWGHT=-FLUENC+DLFLNC
        WGHT=EXP(DLWGHT)
      ELSE
        WGHT=EXP(-FLUENC)*FLUENC
      END IF

      DO I=1,NTARGT(1)
        DO J=1,NTARGT(2)
          CTEMP2(I,J)=CVARSE(I,J)
          CVARME(I,J)=CVARME(I,J)+CTEMP2(I,J)*WGHT
        END DO
      END DO

      DO K=1,2
        TGMEAN(K)=ZERO
        DO I=1,NTARGT(K)
          FNE(I,2,K)=PMARG(I,K+2)
          PMARG(I,K)=PMARG(I,K)+FNE(I,2,K)*WGHT
          IF(DEBUG) TGMEAN(K)=TGMEAN(K)+FNE(I,2,K)*(I-1)
        END DO
      END DO

      IF(DEBUG) THEN
        IF(WGHT.GT.WMIN) THEN
          PRINT*, '#',EVENTS,WGHT,TGMEAN(1)/EVENTS,TGMEAN(2)/EVENTS
        ELSE
          PRINT*, '#',EVENTS,WGHT
        END IF
      END IF


10    CONTINUE
      EVENTS=EVENTS+ONE
      IF(HIFLNC) THEN
        DLWGHT=DLWGHT+DLFLNC-DLOG(EVENTS)
        WGHT=EXP(DLWGHT)
      ELSE
         WGHT=WGHT*FLUENC/EVENTS
      END IF

      DO K=1,2
        TGMEAN(K)=ZERO
        DO I=1,NTARGT(K)
          FNE(I,1,K)=FNE(I,2,K)
          FNE(I,2,K)=ZERO
        END DO
        DO I=1,NTARGT(K)
          DO J=1,NMAXSE(K)
            IJ=I+J-1
```

----- FORTRAN source code of program ME_ROI_3C -----

```
              IF (IJ.GT.NMAXME(K)) IJ=NMAXME(K)
              FNE(IJ,2,K)=FNE(IJ,2,K)+FNE(I,1,K)*PMARG(J,K+2)
            END DO
          END DO
          NTARGT(K)=NTARGT(K)+NMAXSE(K)
          IF(NTARGT(K).GT.NMAXME(K)) NTARGT(K)=NMAXME(K)
          DO I=1,NTARGT(K)
            PMARG(I,K)=PMARG(I,K)+FNE(I,2,K)*WGHT
            IF(DEBUG) TGMEAN(K)=TGMEAN(K)+FNE(I,2,K)*(I-1)
          END DO
        END DO

        DO I=1,NTARGT(1)
          DO J=1,NTARGT(2)
            CTEMP1(I,J)=CTEMP2(I,J)
            CTEMP2(I,J)=ZERO
          END DO
        END DO

        DO I=1,NTARGT(1)
          DO J=1,NTARGT(2)
            DO K=1,I
              DO L=1,J
                CTEMP2(I,J)=CTEMP2(I,J)+
     &                        CTEMP1(K,L)*CVARSE(I+1-K,J+1-L)
              END DO
            END DO
            CVARME(I,J)=CVARME(I,J)+CTEMP2(I,J)*WGHT
          END DO
        END DO

        IF(DEBUG) THEN
          IF(WGHT.GT.WMIN) THEN
            PRINT*, '#',EVENTS,WGHT,TGMEAN(1),TGMEAN(2)
          ELSE
            PRINT*, '#',EVENTS,WGHT
          END IF
        END IF

        IF(EVENTS.LT.FLUENC.OR.WGHT.GT.WMIN) GOTO 10
C!      7777777777777777777777777777777777777777777777777777777777777777
        END IF           ! DUMMY block for readability: Main Loop  777777

        IF(8.EQ.8) THEN ! DUMMY block for readability: Margins 8888888
C!      888888888888888888888888888888888888888888888888888888888888888
        IF(DEBUG) PRINT*, 'Hier beginnt Block 8'
        DO I=1,NMAXME(1)
          DO J=1,NMAXME(2)
            PMARG(I,7)=PMARG(I,7)+CVARME(I,J)
            PMARG(J,8)=PMARG(J,8)+CVARME(I,J)
            IF(CVARME(I,J).GT.WMIN2) THEN
              NTARGT(1)=I
              NTARGT(2)=J
            END IF
          END DO !J=1,NMAXME(2)
        END DO !I=1,NMAXME(1)

        DO I=1,NTARGT(1)
          DO J=1,NTARGT(2)
            IF(PMARG(I,7).GT.WMIN2.AND.PMARG(J,8).GT.WMIN2) THEN
              CORRME(I,J)=CVARME(I,J)/(PMARG(I,7)*PMARG(J,8))
```

----- FORTRAN source code of program ME_ROI_3C -----

```
            ELSE
              CORRME(I,J)=CVARME(I,J)
            END IF
          END DO !I=1,NTARGT(1)
        END DO !J=1,NTARGT(2)

        DO K=1,8
          TGMEAN(K)=ZERO
          SUMS(K)=ZERO
          IF(K.GT.6) NTARGT(K)=NTARGT(K-6)
          DO J=1,NTARGT(K)
            TGMEAN(K)=TGMEAN(K)+PMARG(J,K)*(J-1)
            SUMS(K)=SUMS(K)+PMARG(J,K)
          END DO
        END DO ! DO K=1,8
        PRINT*, 'Distributions averages (#hit targets)'
        WRITE(*,'(10f10.4)') (SUMS(J),J=1,8)
        WRITE(*,'(10f10.4)') (TGMEAN(J),J=1,8)
        WRITE(*,*) TGMEAN(3)/TGMEAN(7),TGMEAN(4)/TGMEAN(8)
C!      8888888888888888888888888888888888888888888888888888888888888
        END IF          ! DUMMY block for readability: Margins 8888888

        IF(9.EQ.9) THEN ! DUMMY block for readability: OUTPUT  9999999
C!      9999999999999999999999999999999999999999999999999999999999999
C!        #Write results to output file
          FILOUT='SEB_'//FILENM
          PRINT*, 'Write output to '//FILOUT
          OPEN(LUN,FILE=FILOUT,STATUS='UNKNOWN')
          WRITE(LUN,*) ' *** Output from PROGRAM ME_ROI_3C Version '
     &                 //VDATE//' on '//TSTAMP()
          WRITE(LUN,*) ' Filename: '//FILOUT
          WRITE(LUN,*) HEADER
          WRITE(LUN,*) ' Correlations P1 and F2 for PVALUE= ',PVALUE
          WRITE(LUN,*) NMAXSE(1), NMAXSE(2)
          DO I=1,NMAXSE(1)
            WRITE(LUN,'(1000D15.6)') (CVARSE(I,J),J=1,NMAXSE(2))
          END DO
          CLOSE(LUN)

          FILOUT='SEC_'//FILENM
          PRINT*, 'Write output to '//FILOUT
          OPEN(LUN,FILE=FILOUT,STATUS='UNKNOWN')
          WRITE(LUN,*) ' *** Output from PROGRAM SE_ROI_3C Version '
     &                 //VDATE//' on '//TSTAMP()
          WRITE(LUN,*) ' Filename: '//FILOUT
          WRITE(LUN,*) HEADER
          WRITE(LUN,*) ' Correlations P1 and F2 for PVALUE= ',PVALUE
          WRITE(LUN,*) NMAXSE(1), NMAXSE(2)
          DO I=1,NMAXSE(1)
            WRITE(LUN,'(1000D15.6)') (CORRSE(I,J),J=1,NMAXSE(2))
          END DO
          CLOSE(LUN)

          FILOUT='MEA_'//FILENM
          PRINT*, 'Write output to '//FILOUT
          OPEN(LUN,FILE=FILOUT,STATUS='UNKNOWN')
          WRITE(LUN,*) ' *** Output from PROGRAM ME_ROI_3C Version '
     &                 //VDATE//' on '//TSTAMP()
          WRITE(LUN,*) ' Filename: '//FILOUT
          WRITE(LUN,*) HEADER
          WRITE(LUN,*) ' Multi and single event distribution for '//
```

----- FORTRAN source code of program ME_ROI_3C -----

```
     &                 'PVALUE= ',PVALUE,'  DOSE= ',DOSE,' Gy'
          WRITE(LUN,*) ' ***  Compared with distributions for ICs ***'

          WRITE(LUN,'(A9,12a15)') '#CVs','P1','F2','P1','F2','P1','F2',
     &                            'P1','F2'

          WRITE(LUN,'(1X,a8,50a15)') 'Data:',('ME',K=1,2),
     &                ('SE',K=1,2),('IC',K=1,2),('ME',K=1,2)

          DO I=1,NMAXIC(1)
            WRITE(LUN,'(1X,i8,10F15.10)') I-1,(PMARG(I,J),J=1,8)
          END DO
          CLOSE(LUN)

          FILOUT='MEB_'//FILENM
          PRINT*, ' Write output to '//FILOUT
          OPEN(LUN,FILE=FILOUT,STATUS='UNKNOWN')
          WRITE(LUN,*) ' *** Output from PROGRAM ME_ROI_3C Version '
     &                 //VDATE//' on '//TSTAMP()
          WRITE(LUN,*) ' Filename: '//FILOUT
          WRITE(LUN,*) HEADER
          WRITE(LUN,*) ' Bivariate freq. P1 and F2 for PVALUE= ',PVALUE
          WRITE(LUN,*) NMAXME(1), NMAXME(2)
          DO I=1,NMAXME(1)
            WRITE(LUN,'(1000D15.6)') (CVARME(I,J),J=1,NMAXME(2))
          END DO
          CLOSE(LUN)

          FILOUT='MEC_'//FILENM
          PRINT*, 'Write output to '//FILOUT
          OPEN(LUN,FILE=FILOUT,STATUS='UNKNOWN')
          WRITE(LUN,*) ' *** Output from PROGRAM ME_ROI_3C Version '
     &                 //VDATE//' on '//TSTAMP()
          WRITE(LUN,*) ' Filename: '//FILOUT
          WRITE(LUN,*) HEADER
          WRITE(LUN,*) ' Correlations P1 and F2 for PVALUE= ',PVALUE
          WRITE(LUN,*) NMAXME(1), NMAXME(2)
          DO I=1,NMAXME(1)
            WRITE(LUN,'(1000D15.6)') (CORRME(I,J),J=1,NMAXME(2))
          END DO
          CLOSE(LUN)

          FILOUT='MED_'//FILENM
          PRINT*, 'Write output to '//FILOUT
          OPEN(LUN,FILE=FILOUT,STATUS='UNKNOWN')
          WRITE(LUN,*) ' *** Output from PROGRAM ME_ROI_3C Version '
     &                 //VDATE//' on '//TSTAMP()
          WRITE(LUN,*) ' Filename: '//FILOUT
          WRITE(LUN,*) HEADER
          WRITE(LUN,*) ' Multi and single event distribution for '//
     &                 'PVALUE= ',PVALUE,'  DOSE= ',DOSE,' Gy'

          WRITE(LUN,'(A9,12a15)') '#CVs','P1','F2','P1','F2'
          WRITE(LUN,'(1X,a8,50a15)') 'Data:',('ME',K=1,2),
     &                ('SE',K=1,2)

          DO I=1,NTARGT(1)
            WRITE(LUN,'(1X,i8,10F15.10)') I-1,(PMARG(I,J),J=1,4)
          END DO
          CLOSE(LUN)
C!      999999999999999999999999999999999999999999999999999999999999999
```

----- FORTRAN source code of program ME_ROI_3C -----

```
         END IF          ! DUMMY block for readability: OUTPUT     9999999
      END DO ! IFILE=1,NFILES

      END PROGRAM ! ME_ROI_3C
C!______________________________________________________________________

        INCLUDE 'TSTAMP.f'
```

FORTRAN source code of program ME_ROI_3D

This is the precursor of ME_ROI_3C where only the marginal distributions are considered and there is no convolution with Binomial distributions so that the results apply to IC distributions. Reads output files 3D_*.dat from ROI_3D, calculates multi event distributions for targets with ionization clusters and produces several output files for further use of the results, named by adding a prefix to the input files:

- ME_*'input_filename'* multi event frequency distributions of targets with one or more than one ionization clusters.
- SE_*'input_filename'* single event frequency distributions of targets with one or more than one ionization clusters.
- TE_*'input_filename'* conditional single event frequency distributions of targets with one or more than one ionization clusters for traversal of the ROI by the primary particle trajectory.
- All_*'input_filename'* all of the three above plus single tracks at certain distances plus tracks passing through annuli around region of interest cross section.

Notes:

- **The program must be executed in the directory where the data files are located**.
- The program expects input of the name of a file listing the debugging options (example see section "Sample debug options file for use with programs IC_3D and ROI_3D").
- Inputs of parameters and input file names are prompted for unless they are entered via a text file. (Manual input is initiated by entering 0 with the first prompt.) A sample input file is listed in the table below.

----- Sample input file for ME_ROI_3D -----

```
210605.17      ! Command file version number ...
ME_ROI_3D      ! ... for this program
1              ! Flag IDEBUG for priniting messages
2.0            ! Absorbed dose in Gy
1              ! Number of input files to process
3B_12.0nm_IC_2.0nm_p3MeVA_mai2015.dat
3.
```

Meaning of the input lines:

(1) Version date and time of the input file structure in format YYMMDD.HHMM
(2) Name of the program
(3) Flag for printing messages on progress (value = 1)
(4) Absorbed dose in Gy
(5) Maximum dimensions of the matrix of bivariate frequencies of the number of targets with a single DSB and multiple DSBs.
(6) Number of input files to process
(7) further odd lines: names of the input files
(8) further even lines: proton energy in MeV

----- FORTRAN source code of program ME_ROI_3D -----

```
      PROGRAM ME_ROI_3D
C!>>> Declarations >>>>>>>>>>>>>>>>>>>>>>>>>>>>>>>>>>>>>>>>>>>>>>>>>>
      IMPLICIT NONE
C!    ----- Parameters: Version Date and number
      CHARACTER VDATE*11
      REAL*8 VINPUT  ! Version number for command files YYMMDD.HHMM
C!                     (date and time  file structure was last changed)
```

----- FORTRAN source code of program ME_ROI_3D -----

```
C!    ################################################################
      PARAMETER(VDATE='05-JUN-2021',VINPUT=210605.1700d0) ! ###########
C!    05-JUN-2021 HR:
C!      - Reorganized initialization of counters.
C!      - Added timestamp in output file
C!    14-APR-2021 HR:
C!      - Adapted to changes done in ROI_3D (leaner output).
C!    31-MAR-2021 HR:
C!      - Fixed bug in initialization of convolution
C!    28-MAR-2021 HR:
C!      - Added one-in-all output for easier transfer to Origin etc.
C!    26-MAR-2021 HR:
C!      - Added option for calculating fluence from dose (based on
C!        new input info of proton energy)
C!    15-MAR-2020 HR:
C!      - Added VDATE and modified COMMON BLOCK VERBOSE
C!    ----- Parameters: General purpose constants
      INTEGER*4 LUN
      REAL*8 ONE, ZERO, PI, WMIN
      PARAMETER(LUN=11, ONE=1.0, ZERO=0.0, PI=3.1415926, WMIN=1d-12)
C!    ----- Parameters for track and radial distance histograms
      INTEGER*4 KMAX, MXRPOS, MXTARG
      PARAMETER(KMAX=9, MXRPOS=101, MXTARG=65537)
C!    ----- Parameters for fluence calculation from power law
C!          regression to PSTAR data between 1 MeV and 100 MeV
      REAL*8 DPERFL  ! Conversion factor from mass stopping power in
                     ! MeV cm²/g to dose per fluence in Gy nm²
      REAL*8 STPWR1  ! Stopping Power at 1 MeV
      REAL*8 STPEXP  ! Exponent of power law for stopping power
      PARAMETER(DPERFL=1.6e4, STPEXP=-0.7899, STPWR1=276.973)
C!    ----- Functions
      CHARACTER TSTAMP*24
C!    ----- Local scalars
      CHARACTER FILENM*120, FILOUT*120, HEADER*1200, PARAM*2,
     &          RINFO(5,2)*11
      INTEGER*4 I, IFILE, IJ, ITARG, J, K, KMX, L, NFILES, NRPOS
      INTEGER*4 NTARG
      LOGICAL ASKINP, DEBUG, HIFLNC
      REAL*8 DBEAM   ! Diameter of beam in nm
      REAL*8 DOSE    ! Absorbed dose in Gray
      REAL*8 DROI    ! Diameter of ROI in nm
      REAL*8 ENERGY  ! Proton energy in MeV
      REAL*8 EVENTS  ! number of events
      REAL*8 FLUENC  ! average number of tracks per ROI cross section
      REAL*8 STPWRE  ! Stopping Power at energy E
      REAL*8 DLFLNC, DLWGHT, SPOSIN, SUMPOS, VCHECK, WGHT, TGMEAN(KMAX)
C!    ----- Local arrays
      INTEGER*4 JMAX(2),NTARGK(KMAX),NTARGT(KMAX)
      REAL*8 FREQSE(MXTARG,KMAX),FREQTE(MXTARG,KMAX),
     &       FREQME(MXTARG,KMAX),FNE(MXTARG,2,KMAX),
     &       FREQST(MXRPOS), FREQTR(MXTARG,KMAX,5),
     &       FREQAR(MXTARG,KMAX,5), RELFLU(5)
C!<<<<End declarations<<<<<<<<<<<<<<<<<<<<<<<<<<<<<<<<<<<<<<<<<<<<<<<<

      PRINT*, 'Program ME_ROI_3D Version: '//VDATE

      IF(1.EQ.1) THEN ! DUMMY block for readability: Read input 1111111

C!      Check command file version
        PRINT*, 'Enter 0 for manual input or version (YYMMDD.HHMM) '//
     &          'of command file structure '
```

----- FORTRAN source code of program ME_ROI_3D -----

```
        READ(*,*) VCHECK
        ASKINP=(VCHECK.EQ.ZERO)
        IF(.NOT.ASKINP) THEN
          IF(VCHECK.LT.VINPUT) THEN
            PRINT*, 'Command file structure ',VCHECK,' older than '//
     &              'current version ',VINPUT,'=> STOP.'
            STOP
          END IF
          READ(*,*) FILENM
          IF(FILENM(1:9).NE.'ME_ROI_3D') THEN
            PRINT*, 'Command file appear not to be for ROI_3D but '//
     &              'for '//FILENM//'=> STOP.'
            STOP
          END IF
        END IF

        IF(ASKINP) PRINT*, 'Run in debug mode? (1/0)'
        READ(*,*) I
        DEBUG=(I.EQ.1)

        IF(ASKINP) PRINT*, 'Absorbed dose in Gy'
        READ(*,*) DOSE

        IF(ASKINP) PRINT*, 'Number of files to process'
        READ(*,*) NFILES

      END IF          ! DUMMY block for readability: Read input 1111111

      IF(2.EQ.2) THEN ! DUMMY block for readability: Initialize 2222222
        RINFO(1,1)='        R=0'
        RINFO(2,1)='R=0.75R_ROI'
        RINFO(3,1)='    R=R_ROI'
        RINFO(4,1)=' R=1.5R_ROI'
        RINFO(5,1)='   R=2R_ROI'
        RINFO(1,2)='    R<R_ROI'
        RINFO(2,2)='1<R/R_ROI<2'
        RINFO(3,2)='2<R/R_ROI<3'
        RINFO(4,2)='3<R/R_ROI<4'
        RINFO(5,2)='4<R/R_ROI<5'
        NTARG=MXTARG
      END IF          ! DUMMY block for readability: Initialize 2222222

      DO IFILE=1,NFILES
        IF(ASKINP) PRINT*, 'Enter file name ',IFILE
        READ(*,*) FILENM
        IF(.NOT.ASKINP) PRINT*, 'Processing file '//FILENM
        IF(ASKINP) PRINT*, 'Enter related proton energy '
        READ(*,*) ENERGY
        STPWRE=STPWR1*EXP(STPEXP*LOG(ENERGY))

        IF(3.EQ.3) THEN ! DUMMY block for readability: Init counters 3333
          DO K=1,KMAX
            DO I=1,MXTARG
              FREQSE(I,K)=ZERO
              FREQTE(I,K)=ZERO
              FREQME(I,K)=ZERO
              DO J=1,2
                FNE(I,J,K)=ZERO
              END DO
              DO J=1,5
                FREQTR(I,K,J)=ZERO
```

----- FORTRAN source code of program ME_ROI_3D -----

```
                FREQAR(I,K,J)=ZERO
              END DO
            END DO !I=1,MXTARG
            TGMEAN(K)=ZERO
          END DO ! DO K=1,KMAX
        END IF          ! DUMMY block for readability: Init counters 3333

        IF(4.EQ.4) THEN ! DUMMY block for readability: Get data  444444
          IF(DEBUG) PRINT*, 'Begin of Block 4'

          OPEN(LUN,FILE=FILENM,STATUS='OLD')
          DO I=1,2
            READ(LUN,'(A80)') HEADER
          END DO
          READ(LUN,'(10X,10i6)') (NTARGK(I),I=1,KMAX)
          DO I=1,KMAX
            IF(NTARGK(I).GT.0) KMX=I
          END DO

          READ(LUN,'(A80)') HEADER
          READ(HEADER(28:36),*) DROI
          READ(HEADER(69:77),*) DBEAM
          NRPOS=1+NINT(DBEAM/DROI*20.)
          IF(NRPOS.GE.41) THEN
            JMAX(1)=5
          ELSE
            JMAX(1)=4
            IF(NRPOS.LT.16) JMAX(1)=1
            IF(NRPOS.LT.21) JMAX(1)=2
            IF(NRPOS.LT.31) JMAX(1)=3
          END IF
          IF(NRPOS.EQ.101) THEN
            JMAX(2)=5
          ELSE
            JMAX(2)=4
            IF(NRPOS.LT.41) JMAX(2)=1
            IF(NRPOS.LT.61) JMAX(2)=2
            IF(NRPOS.LT.81) JMAX(2)=3
          END IF
C!          FLUENC=DROI*DROI*PI/4.*DOSE/DPERFL/STPWRE
C!        Single-event distribution uses 20*RROI as max. impact parameter
          FLUENC=0.25*DBEAM*DBEAM*PI*DOSE/DPERFL/STPWRE
          RELFLU(1)=DROI*DROI/(DBEAM*DBEAM)
          SPOSIN=ONE
          DO I=2,5
            SPOSIN=SPOSIN+2.
            RELFLU(I)=RELFLU(1)*SPOSIN
          END DO
          PRINT*, DOSE,ENERGY,STPWRE,FLUENC,DROI,DBEAM,NRPOS

          DO K=1,KMX
            DO J=1,2
              READ(LUN,*) PARAM
            END DO
            DO J=1,NTARGK(K)
              READ(LUN,*) PARAM,ITARG,FREQSE(J,K), FREQTE(J,K),
     &                    (FREQST(L),L=1,NRPOS)
              FREQTR(J,K,1)=FREQST(1)
              IF(NRPOS.GE.16) FREQTR(J,K,2)=FREQST(16)
              IF(NRPOS.GE.21) FREQTR(J,K,3)=FREQST(21)
              IF(NRPOS.GE.31) FREQTR(J,K,4)=FREQST(31)
```

----- FORTRAN source code of program ME_ROI_3D -----

```
              IF(NRPOS.GE.41) FREQTR(J,K,5)=FREQST(41)
              L=1
              SPOSIN=ZERO
              SUMPOS=ONE
              FREQAR(J,K,L)=FREQST(1)
              DO IJ=20*L-18,20*L+1
                SPOSIN=SPOSIN+8.
                FREQAR(J,K,L)=FREQAR(J,K,L)+SPOSIN*FREQST(IJ)
                SUMPOS=SUMPOS+SPOSIN
              END DO
              FREQAR(J,K,L)=FREQAR(J,K,L)*RELFLU(L)/SUMPOS
              DO L=2,JMAX(2)
                FREQAR(J,K,L)=ZERO
                SUMPOS=ZERO
                DO IJ=20*L-18,20*L+1
                  SPOSIN=SPOSIN+8.
                  FREQAR(J,K,L)=FREQAR(J,K,L)+SPOSIN*FREQST(IJ)
                  SUMPOS=SUMPOS+SPOSIN
                END DO
                FREQAR(J,K,L)=FREQAR(J,K,L)*RELFLU(L)/SUMPOS
              END DO
            END DO
            DO J=1,2
              READ(LUN,*) PARAM
            END DO
          END DO
          CLOSE(LUN)
        END IF          ! DUMMY block for readability: Get data  4444444

        IF(5.EQ.5) THEN ! DUMMY block for readability: Main loop  5555555
          IF(DEBUG) PRINT*, 'Begin of Block 5'

C!        Init counters
          DO K=1,KMX
            FREQME(1,K)=EXP(-FLUENC) ! Kronecker's delta for J=1 (0
targets)
          END DO

          EVENTS=ONE
          HIFLNC=(FLUENC.GT.23.4)
          IF(HIFLNC) THEN
            DLFLNC=DLOG(FLUENC)
            DLWGHT=-FLUENC+DLFLNC
            WGHT=EXP(DLWGHT)
          ELSE
             WGHT=EXP(-FLUENC)*FLUENC
          END IF

          DO K=1,KMX
            DO J=1,NTARGK(K)
              FNE(J,2,K)=FREQSE(J,K)
              IF(DEBUG) TGMEAN(K)=TGMEAN(K)+FNE(J,2,K)*(J-1)
              FREQME(J,K)=FREQME(J,K)+FNE(J,2,K)*WGHT
            END DO
            NTARGT(K)=NTARGK(K)
          END DO ! DO K=1,KMX

          IF(DEBUG) THEN
            IF(WGHT.GT.WMIN) THEN
              PRINT*, '#',EVENTS,WGHT,TGMEAN(1)/EVENTS,TGMEAN(2)/EVENTS
            ELSE
```

----- FORTRAN source code of program ME_ROI_3D -----

```
              PRINT*, '#',EVENTS,WGHT
            END IF
          END IF

   10     CONTINUE
          EVENTS=EVENTS+ONE
          IF(HIFLNC) THEN
            DLWGHT=DLWGHT+DLFLNC-DLOG(EVENTS)
            WGHT=EXP(DLWGHT)
          ELSE
             WGHT=WGHT*FLUENC/EVENTS
          END IF

          DO K=1,KMX
            TGMEAN(K)=ZERO
            DO J=1,NTARGT(K)
              FNE(J,1,K)=FNE(J,2,K)
              FNE(J,2,K)=ZERO
            END DO
            DO J=1,NTARGT(K)
              DO I=1,NTARGK(K)
                IJ=I+J-1
                IF (IJ.GT.MXTARG) IJ=MXTARG
                FNE(IJ,2,K)=FNE(IJ,2,K)+FNE(J,1,K)*FREQSE(I,K)
              END DO
            END DO
            NTARGT(K)=NTARGT(K)+NTARGK(K)
            IF(NTARGT(K).GT.MXTARG) NTARGT(K)=MXTARG
            DO J=1,NTARGT(K)
              FREQME(J,K)=FREQME(J,K)+FNE(J,2,K)*WGHT
              IF(DEBUG) TGMEAN(K)=TGMEAN(K)+FNE(J,2,K)*(J-1)
            END DO
          END DO ! DO K=1,KMX

          IF(DEBUG) THEN
            IF(WGHT.GT.WMIN) THEN
              PRINT*, '#',EVENTS,WGHT,TGMEAN(1),TGMEAN(2)
            ELSE
              PRINT*, '#',EVENTS,WGHT
            END IF
          END IF

          IF(EVENTS.LT.FLUENC.OR.WGHT.GT.WMIN) GOTO 10  ! >>>>>>>>>>
C!        End of inner loop

          DO K=1,KMX
            TGMEAN(K)=ZERO
            DO J=1,NTARGK(K)
              TGMEAN(K)=TGMEAN(K)+FREQSE(J,K)*(J-1)
            END DO
          END DO ! DO K=1,KMX
          PRINT*, 'SE distributions averages (#hit targets) K=1,',KMX
          PRINT*, (TGMEAN(K),K=1,KMX)
          PRINT*, (TGMEAN(K)/EVENTS,K=1,KMX)

          DO K=1,KMX
            TGMEAN(K)=ZERO
            DO J=1,NTARGK(K)
              TGMEAN(K)=TGMEAN(K)+FREQTE(J,K)*(J-1)
            END DO
          END DO ! DO K=1,KMX
```

----- FORTRAN source code of program ME_ROI_3D -----

```
          PRINT*, 'TE distributions averages (#hit targets) K=1,',KMX
          PRINT*, (TGMEAN(K),K=1,KMX)
          PRINT*, (TGMEAN(K)/EVENTS,K=1,KMX)

          DO K=1,KMX
            TGMEAN(K)=ZERO
            DO J=1,NTARGT(K)
              TGMEAN(K)=TGMEAN(K)+FREQME(J,K)*(J-1)
            END DO
          END DO ! DO K=1,KMX
          PRINT*, 'ME distributions averages (#hit targets) K=1,',KMX
          PRINT*, (TGMEAN(K),K=1,KMX)
          PRINT*, (TGMEAN(K)/EVENTS,K=1,KMX)

        END IF          ! DUMMY block for readability: Main Loop  555555

        IF(9.EQ.9) THEN ! DUMMY block for readability: OUTPUT  9999999
C!        #Write results to output file
          FILOUT='SE_'//FILENM
          PRINT*, 'Write output to '//FILOUT
          OPEN(LUN,FILE=FILOUT,STATUS='UNKNOWN')
          WRITE(LUN,*) '*** Output from PROGRAM ME_ROI_3D Version '
     &                //VDATE//' on '//TSTAMP()
          WRITE(LUN,*) 'Filename: '//FILOUT
          WRITE(LUN,*) HEADER
          WRITE(LUN,'(1X,2a8,9(a14,i1))') '#Sites','P1',('F',J,J=2,KMX)
          DO I=1,NTARGK(1)
            SUMPOS=ZERO
            DO K=1,KMX
              IF(SUMPOS.LT.FREQSE(I,K)) SUMPOS=FREQSE(I,K)
            END DO
            IF(SUMPOS.GT.WMIN) NTARG=I
          END DO
          DO I=1,NTARG
            WRITE(LUN,'(1X,i8,10f15.8)') I-1,(FREQSE(I,K),K=1,KMX)
          END DO
          CLOSE(LUN)

          FILOUT='TE_'//FILENM
          PRINT*, 'Write output to '//FILOUT
          OPEN(LUN,FILE=FILOUT,STATUS='UNKNOWN')
          WRITE(LUN,*) '*** Output from PROGRAM ME_ROI_3D Version '
     &                //VDATE//' on '//TSTAMP()
          WRITE(LUN,*) 'Filename: '//FILOUT
          WRITE(LUN,*) HEADER
          WRITE(LUN,'(1X,2a8,9(a14,i1))') '#Sites','P1',('F',J,J=2,KMX)
          DO I=1,NTARGK(1)
            SUMPOS=ZERO
            DO K=1,KMX
              IF(SUMPOS.LT.FREQTE(I,K)) SUMPOS=FREQTE(I,K)
            END DO
            IF(SUMPOS.GT.WMIN) NTARG=I
          END DO
          DO I=1,NTARG
            WRITE(LUN,'(1X,i8,10f15.6)') I-1,(FREQTE(I,K),K=1,KMX)
          END DO
          CLOSE(LUN)

          FILOUT='ME_'//FILENM
          PRINT*, 'Write output to '//FILOUT
          OPEN(LUN,FILE=FILOUT,STATUS='UNKNOWN')
```

----- FORTRAN source code of program ME_ROI_3D -----

```
      WRITE(LUN,*) '*** Output from PROGRAM ME_ROI_3D Version '
     &              //VDATE//' on '//TSTAMP()
      WRITE(LUN,*) 'Filename: '//FILOUT
      WRITE(LUN,*) HEADER
      WRITE(LUN,*) 'DOSE= ',DOSE,' Gy'
      WRITE(LUN,'(1X,2a8,9(a14,i1))') '#Sites','P1',('F',J,J=2,KMX)
      DO I=1,NTARGT(1)
        SUMPOS=ZERO
        DO K=1,KMX
          IF(SUMPOS.LT.FREQME(I,K)) SUMPOS=FREQME(I,K)
        END DO
        IF(SUMPOS.GT.WMIN) NTARG=I
      END DO
      DO I=1,NTARG
        WRITE(LUN,'(1X,i8,10f15.6)') I-1,(FREQME(I,K),K=1,KMX)
      END DO
      CLOSE(LUN)

      FILOUT='ALL_'//FILENM
      KMX=2
      PRINT*, 'Write output to '//FILOUT
      OPEN(LUN,FILE=FILOUT,STATUS='UNKNOWN')
      WRITE(LUN,*) '*** Output from PROGRAM ME_ROI_3D Version '
     &              //VDATE//' on '//TSTAMP()
      WRITE(LUN,*) 'Filename: '//FILOUT
      WRITE(LUN,*) HEADER
      WRITE(LUN,*) 'DOSE= ',DOSE,' Gy'

      WRITE(LUN,'(1X,a8,50(a14,i1))') '#Sites',
     &                 'P',1,('F',K,K=2,KMX),'P',1,('F',K,K=2,KMX),
     &                 'P',1,('F',K,K=2,KMX),'P',1,('F',K,K=2,KMX),
     &                 'P',1,('F',K,K=2,KMX),'P',1,('F',K,K=2,KMX),
     &                 'P',1,('F',K,K=2,KMX),'P',1,('F',K,K=2,KMX),
     &                 'P',1,('F',K,K=2,KMX),'P',1,('F',K,K=2,KMX),
     &                 'P',1,('F',K,K=2,KMX),'P',1,('F',K,K=2,KMX),
     &                 'P',1,('F',K,K=2,KMX)

      WRITE(LUN,'(1X,a8,50a15)') 'Data:',('ME',K=1,KMX),
     &              ('SE',K=1,KMX),('TE',K=1,KMX),
     &              ((RINFO(J,2),K=1,KMX),J=1,JMAX(2)),
     &              ((RINFO(J,1),K=1,KMX),J=1,JMAX(1))

      DO I=1,NTARGT(1)
        SUMPOS=ZERO
        DO K=1,KMX
          IF(SUMPOS.LT.FREQSE(I,K)) SUMPOS=FREQSE(I,K)
          IF(SUMPOS.LT.FREQTE(I,K)) SUMPOS=FREQTE(I,K)
          IF(SUMPOS.LT.FREQME(I,K)) SUMPOS=FREQME(I,K)
          DO J=1,JMAX(1)
            IF(SUMPOS.LT.FREQTR(I,K,J)) SUMPOS=FREQTR(I,K,J)
          END DO
          DO J=1,JMAX(2)
            IF(SUMPOS.LT.FREQAR(I,K,J)) SUMPOS=FREQAR(I,K,J)
          END DO
        END DO
        IF(SUMPOS.GT.WMIN) NTARG=I
      END DO
      DO I=1,NTARG
        WRITE(LUN,'(1X,i8,50e15.6)') I-1,(FREQME(I,K),K=1,KMX),
     &              (FREQSE(I,K),K=1,KMX),(FREQTE(I,K),K=1,KMX),
     &              ((FREQAR(I,K,J),K=1,KMX),J=1,JMAX(2))      ,
```

----- FORTRAN source code of program ME_ROI_3D -----

```
     &                 ((FREQTR(I,K,J),K=1,KMX),J=1,JMAX(1))
            END DO
            CLOSE(LUN)

          END IF          ! DUMMY block for readability: OUTPUT     9999999
        END DO ! IFILE=1,NFILES

        END PROGRAM ! ME_ROI_3D
C!_____________________________________________________________________

        INCLUDE 'TSTAMP.f'
```

FORTRAN source code of program ME_ROI_3P

This is a lean and much faster variant of ME_ROI_3C where only the marginal distributions are considered. The program uses the bivariate distributions output from ROI_3D (3B_*.dat) and convolutes them with Binomial distributions of a given success probability such as to convert IC to DSB distributions.

Output:

- MEP_*'input_filename'* multi- and single event frequency distributions after convolution with binomial compared to frequency distributions of ionization clusters.

Notes:

- **The program must be executed in the directory where the data files are located**.
- The program expects input of the name of a file listing the debugging options (example see section "Sample debug options file for use with programs IC_3D and ROI_3D").
- Inputs of parameters and input file names are prompted for unless they are entered via a text file. (Manual input is initiated by entering 0 with the first prompt.) A sample input file is listed in the table below.

----- Sample input file for ME_ROI_3P -----

```
210605.16      ! Command file version number ...
ME_ROI_3P      ! ... for this program
1   100        ! Flags IDEBUG, IMESSG
0.01           ! Relative target density
6              ! Number of dose values
0.5 1.0 2.0 4.0 8.0 16.0 ! Absorbed doses in Gy
3B_12.0nm_IC_2.0nm_p3MeVA_mai2015.dat
3.
```

Meaning of the input lines:

(1) Version date and time of the input file structure in format YYMMDD.HHMM
(2) Name of the program
(3) Flag for printing messages on progress (value = 1) and number of iterations between successive outputs
(4) Relative target density (success probability of the binomial)
(5) Number of dose values in the next line
(6) Absorbed doses in Gy
(7) further odd lines: names of the input files
(8) further even lines: proton energy in MeV

----- FORTRAN source code of program ME_ROI_3P -----

```
      PROGRAM ME_ROI_3P
C!>>> Declarations >>>>>>>>>>>>>>>>>>>>>>>>>>>>>>>>>>>>>>>>>>>>>>>>>>
      IMPLICIT NONE
C!    ----- Parameters: Version Date and number
      CHARACTER VDATE*11
      REAL*8 VINPUT  ! Version number for command files YYMMDD.HHMM
C!                     (date and time  file structure was last changed)
C!    ################################################################
      PARAMETER(VDATE='05-JUN-2021',VINPUT=210605.1600d0) ! ###########
C!    05-JUN-2021 HR:
```

----- FORTRAN source code of program ME_ROI_3P -----

```
C!      - Reorganized command input file.
C!      - Added timestamp in output file
C!    02-MAY-2021 HR:
C!      - Introduced PREFIX to output file name for inclusion of dose.
C!    15-APR-2021 HR:
C!      - Adapted to changes done in ROI_3D (leaner output).
C!    13-APR-2021 HR:
C!      - Created from ME_ROI_3D
C!    ----- Purpose
C!    Calculate multi-ebent distribution of targets with single and
C!    and multiple ionization clusters after filtering with Bernoulli
C!    process.
C!    ----- Parameters: General purpose constants
      INTEGER*4 LUN
      REAL*8 ONE, ZERO, PI, WMIN, WMIN2
      PARAMETER(LUN=11, ONE=1.0, ZERO=0.0, PI=3.1415926, WMIN=1d-10,
     &          WMIN2=1d-40)
C!    ----- Parameters for track and radial distance histograms
      INTEGER*4 MXTARG, MXTRG1, MXTRG2, MXNDOS, MXNCOL
      PARAMETER(MXTARG=65636, MXTRG1=1025, MXTRG2=513, MXNDOS=8,
     &          MXNCOL=2*MXNDOS+4)
C!    ----- Parameters for fluence calculation from power law
C!          regression to PSTAR data between 1 MeV and 100 MeV
      REAL*8 DPERFL  ! Conversion factor from mass stopping power in
                     ! MeV cm²/g to dose per fluence in Gy nm²
      REAL*8 STPWR1  ! Stopping Power at 1 MeV
      REAL*8 STPEXP  ! Exponent of power law for stopping power
      PARAMETER(DPERFL=1.6e4, STPEXP=-0.7899, STPWR1=276.973)
C!    ----- Functions
      CHARACTER TSTAMP*24
C!    ----- Local scalars
      CHARACTER FILENM*120, FILOUT*120, HEADER*120, PREFIX*4
      INTEGER*4 I, IDOSE, IFILE, IJ, IMESSG(2), J, K, L, NCOLS, NDOSES,
     &          NFILES
      LOGICAL ASKINP, DEBUG(2), HIFLNC
      REAL*8 DBEAM   ! Diameter of beam in nm
      REAL*8 DROI    ! Diameter of ROI in nm
      REAL*8 ENERGY  ! Proton energy in MeV
      REAL*8 EVENTS  ! number of events
      REAL*8 FLUENC  ! average number of tracks per ROI cross section
      REAL*8 PVALUE  ! Probability that cluster volume is target
      REAL*8 QVALUE  ! Probability that cluster volume is not a target
      REAL*8 STPWRE  ! Stopping Power at energy E
      REAL*8 DLFLNC, DLWGHT, VCHECK, WGHT
C!    ----- Local arrays
      CHARACTER CDOSES(MXNDOS)*7
      INTEGER*4 ICOL(2), ICOLIC(2), ICOLSE(2)
      INTEGER*4 NMAX(2), NMAXME(2), NMAXSE(2), NMCMAX(MXTRG1), NTARGT(8)
      REAL*8 ADEN(2), ANUM(2), APOT(2), PTOK(2), WEIGH(2)
      REAL*8 DOSE(MXNDOS)     ! Absorbed dose in Gray
      REAL*8 BVARIC(MXTRG1,MXTRG2)
      REAL*8 PMARG(MXTARG,MXNCOL)
      REAL*8 CORRSE(MXTRG1,MXTRG2),BVARSE(MXTRG1,MXTRG2)
      REAL*8 SUMS(MXNCOL),TGMEAN(MXNCOL),FNE(MXTARG,2,2)
C!<<<<End declarations<<<<<<<<<<<<<<<<<<<<<<<<<<<<<<<<<<<<<<<<<<<<<<<<

      PRINT*, 'Program ME_ROI_3P Version: '//VDATE

      IF(1.EQ.1) THEN ! DUMMY block for readability: Read input 1111111
C!      Check command file version
        PRINT*, 'Enter 0 for manual input or version (YYMMDD.HHMM) '//
```

----- FORTRAN source code of program ME_ROI_3P -----

```
     &          'of command file structure '
        READ(*,*) VCHECK
        ASKINP=(VCHECK.EQ.ZERO)
        IF(.NOT.ASKINP) THEN
          IF(VCHECK.LT.VINPUT) THEN
            PRINT*, 'Command file structure ',VCHECK,' older than '//
     &              'current version ',VINPUT,'=> STOP.'
            STOP
          END IF
          READ(*,*) FILENM
          IF(FILENM(1:9).NE.'ME_ROI_3P') THEN
            PRINT*, 'Command file appear not to be for ROI_3D but '//
     &              'for '//FILENM//'=> STOP.'
            STOP
          END IF
        END IF

        IF(ASKINP) THEN
          PRINT*, 'Run in debug mode? (1/0) '
          READ(*,*) IMESSG(1)
          PRINT*, 'Interval between printing intermediate results '//
     &            '(0=suppress this output) '
          READ(*,*) IMESSG(2)
        ELSE
          READ(*,*) (IMESSG(I),I=1,2)
        END IF
        DEBUG(1)=(IMESSG(1).EQ.1)
        DEBUG(2)=(IMESSG(2).GE.1)
        ASKINP=ASKINP.OR.DEBUG(1)

        PRINT*, 'Relative target density (0<PVALUE<1)'
        READ(*,*) PVALUE
        QVALUE=ONE-PVALUE
        IF(ASKINP) PRINT*, 'Number of dose values (<=',MXNDOS,')'
        READ(*,*) NDOSES
        IF(NDOSES.GT.MXNDOS) NDOSES=MXNDOS
        IF(ASKINP) PRINT*, 'Absorbed doses in Gy'
        READ(*,*) (DOSE(I),I=1,NDOSES)

        IF(ASKINP) PRINT*, 'Number of files to process'
        READ(*,*) NFILES
      END IF          ! DUMMY block for readability: Read input 1111111

      IF(2.EQ.2) THEN ! DUMMY block for readability: Initialize 2222222
        DO I=1,2
          ICOLSE(I)=2*NDOSES+I
          ICOLIC(I)=ICOLSE(I)+2
        END DO
        NMAXME(1)=MXTARG
        NMAXME(2)=MXTARG
      END IF          ! DUMMY block for readability: Initialize 2222222

      DO IFILE=1,NFILES
        IF(ASKINP) PRINT*, 'Enter file name ',IFILE
        READ(*,*) FILENM
        IF(ASKINP) PRINT*, 'Enter related proton energy '
        READ(*,*) ENERGY
        STPWRE=STPWR1*EXP(STPEXP*LOG(ENERGY))

        IF(3.EQ.3) THEN ! DUMMY block for readability: Init counters 3333
          DO K=1,2
```

----- FORTRAN source code of program ME_ROI_3P -----

```
          DO I=1,MXTARG
            DO J=1,2
              FNE(I,J,K)=ZERO
            END DO
          END DO !I=1,MXTARG
          TGMEAN(K)=ZERO
        END DO ! DO K=1,2
      END IF          ! DUMMY block for readability: Init counters 3333

      IF(4.EQ.4) THEN ! DUMMY block for readability: Get data  444444
        IF(DEBUG(1)) PRINT*, 'Begin of Block 4'

        OPEN(LUN,FILE=FILENM,STATUS='OLD')
        DO I=1,2
          READ(LUN,'(A80)') HEADER
        END DO

        READ(LUN,'(A80)') HEADER
        PRINT*, FILENM
        PRINT*, HEADER
        READ(HEADER(28:36),*) DROI
        READ(HEADER(69:77),*) DBEAM
        PRINT*, (DOSE(J),J=1,NDOSES),ENERGY,STPWRE,DROI,DBEAM

        READ(LUN,'(A)') FILOUT
        READ(LUN,*) NMAX(1), NMAX(2)

        DO J=1,NMAX(1)
          READ(LUN,*) (BVARIC(J,L),L=1,NMAX(2))
        END DO

        CLOSE(LUN)

        DO J=1,NMAX(1)
          NMCMAX(J)=0
        END DO

        DO J=1,NMAX(1)
          DO L=1,NMAX(2)
            BVARSE(J,L)=ZERO
            CORRSE(J,L)=ZERO
            IF(BVARIC(J,L).GT.WMIN2) THEN
              NMCMAX(J)=L
            END IF
          END DO
        END DO

      END IF          ! DUMMY block for readability: Get data 4444444

      IF(5.EQ.5) THEN ! DUMMY block for readability: Convolve 5555555
        IF(DEBUG(1)) PRINT*, 'Begin of Block 5 '
        APOT(1)=ZERO
        PTOK(1)=ONE
        DO I=1,NMAX(1)
          APOT(2)=ZERO
          PTOK(2)=ONE
          DO J=1,NMAX(2)
            ANUM(1)=APOT(1)
            ADEN(1)=ZERO
            WEIGH(1)=PTOK(1)
            DO K=I,NMAX(1)
```

----- FORTRAN source code of program ME_ROI_3P -----

```
              IF(WEIGH(1).GT.WMIN2) THEN
                ANUM(2)=APOT(2)
                ADEN(2)=ZERO
                WEIGH(2)=PTOK(2)*WEIGH(1)
                DO L=J,NMCMAX(K)
                  IF(WEIGH(2).GT.WMIN2) THEN
                    BVARSE(I,J)=BVARSE(I,J)+WEIGH(2)*BVARIC(K,L)
                    ADEN(2)=ADEN(2)+ONE
                    ANUM(2)=ANUM(2)+ONE
                    WEIGH(2)=WEIGH(2)*QVALUE*ANUM(2)/ADEN(2)
                  END IF
                END DO ! L=J,NMAX(2)
                ADEN(1)=ADEN(1)+ONE
                ANUM(1)=ANUM(1)+ONE
                WEIGH(1)=WEIGH(1)*QVALUE*ANUM(1)/ADEN(1)
              END IF
            END DO ! K=I,NMAX(1)
            APOT(2)=APOT(2)+ONE
            PTOK(2)=PTOK(2)*PVALUE
          END DO !J=1,NMAX(2)
          APOT(1)=APOT(1)+ONE
          PTOK(1)=PTOK(1)*PVALUE
        END DO !I=1,NMAX(1)
      END IF          ! DUMMY block for readability: Convolve 555555

      IF(5.EQ.5) THEN ! DUMMY block for readability: Margins 6666666
        IF(DEBUG(1)) PRINT*, 'Begin of Block 6'
        DO I=1,MXTARG
          DO K=1,MXNCOL
            PMARG(I,K)=ZERO
          END DO
        END DO

        DO I=1,NMAX(1)
          DO J=1,NMAX(2)
            PMARG(I,ICOLIC(1))=PMARG(I,ICOLIC(1))+BVARIC(I,J)
            PMARG(J,ICOLIC(2))=PMARG(J,ICOLIC(2))+BVARIC(I,J)
            IF(BVARSE(I,J).GT.WMIN2) THEN
              NMAXSE(1)=I
              NMAXSE(2)=J
            END IF
          END DO !I=1,NMAX(1)
        END DO !J=1,NMAX(2)

        DO I=1,NMAXSE(1)
          DO J=1,NMAXSE(2)
            PMARG(I,ICOLSE(1))=PMARG(I,ICOLSE(1))+BVARSE(I,J)
            PMARG(J,ICOLSE(2))=PMARG(J,ICOLSE(2))+BVARSE(I,J)
          END DO !I=1,NMAXSE(1)
        END DO !J=1,NMAXSE(2)

        DO I=1,NMAXSE(1)
          DO J=1,NMAXSE(2)
            IF(PMARG(I,ICOLSE(1)).GT.WMIN2.AND.
     &         PMARG(J,ICOLSE(2)).GT.WMIN2) THEN
              CORRSE(I,J)=BVARSE(I,J)
     &                    /(PMARG(I,ICOLSE(1))*PMARG(J,ICOLSE(2)))
            ELSE
              CORRSE(I,J)=BVARSE(I,J)
            END IF
          END DO !I=1,NMAXSE(1)
```

----- FORTRAN source code of program ME_ROI_3P -----

```
          END DO !J=1,NMAXSE(2)

        END IF          ! DUMMY block for readability: Margins 6666666

        IF(7.EQ.7) THEN ! DUMMY block for readability: Main loop  777777
          DO IDOSE=1,NDOSES
            IF(DEBUG(1)) PRINT*, 'Begin of Block 6, dose =',DOSE(IDOSE)

C!        Init counters

            FLUENC=0.25*DBEAM*DBEAM*PI*DOSE(IDOSE)/DPERFL/STPWRE
            DO K=1,2
              NTARGT(K)=NMAXSE(K)
              ICOL(K)=2*(IDOSE-1)+K
              PMARG(1,ICOL(K))=EXP(-FLUENC) ! Kronecker's delta for J=1
(0 targets)
            END DO

            EVENTS=ONE
            HIFLNC=(FLUENC.GT.23.4)
            IF(HIFLNC) THEN
              DLFLNC=DLOG(FLUENC)
              DLWGHT=-FLUENC+DLFLNC
              WGHT=EXP(DLWGHT)
            ELSE
              WGHT=EXP(-FLUENC)*FLUENC
            END IF

             DO K=1,2
              TGMEAN(ICOL(K))=ZERO
              DO I=1,NTARGT(K)
                FNE(I,2,K)=PMARG(I,ICOLSE(K))
                PMARG(I,ICOL(K))=PMARG(I,ICOL(K))+FNE(I,2,K)*WGHT
                IF(DEBUG(2)) TGMEAN(ICOL(K))=TGMEAN(ICOL(K))
     &                                         +FNE(J,2,K)*(J-1)
              END DO
            END DO

            IF(DEBUG(1)) THEN
              IF(WGHT.GT.WMIN) THEN
                PRINT*, '#',EVENTS,WGHT,TGMEAN(ICOL(1))/EVENTS,
     &                  TGMEAN(ICOL(2))/EVENTS
              ELSE
                PRINT*, '#',EVENTS,WGHT
              END IF
            END IF

   10       CONTINUE
            EVENTS=EVENTS+ONE
            IF(HIFLNC) THEN
              DLWGHT=DLWGHT+DLFLNC-DLOG(EVENTS)
              WGHT=EXP(DLWGHT)
            ELSE
               WGHT=WGHT*FLUENC/EVENTS
            END IF

            DO K=1,2
              DO I=1,NTARGT(K)
                FNE(I,1,K)=FNE(I,2,K)
                FNE(I,2,K)=ZERO
              END DO
```

----- FORTRAN source code of program ME_ROI_3P -----

```
            DO I=1,NTARGT(K)
              DO J=1,NMAXSE(K)
                IJ=I+J-1
                IF (IJ.GT.NMAXME(K)) IJ=NMAXME(K)
                FNE(IJ,2,K)=FNE(IJ,2,K)+FNE(I,1,K)*PMARG(J,ICOLSE(K))
              END DO
            END DO
            NTARGT(K)=NTARGT(K)+NMAXSE(K)
            IF(NTARGT(K).GT.NMAXME(K)) NTARGT(K)=NMAXME(K)
            DO I=1,NTARGT(K)
              PMARG(I,ICOL(K))=PMARG(I,ICOL(K))+FNE(I,2,K)*WGHT
            END DO
          END DO

          IF(DEBUG(2).AND.MOD(NINT(EVENTS),IMESSG(2)).EQ.0) THEN
            IF(WGHT.GT.WMIN) THEN
              DO K=1,2
                TGMEAN(ICOL(K))=ZERO
                DO J=1,NTARGT(K)
                  TGMEAN(ICOL(K))=TGMEAN(ICOL(K))+FNE(J,2,K)*(J-1)
                END DO
              END DO
              PRINT*, '#',EVENTS,WGHT,TGMEAN(ICOL(1)),TGMEAN(ICOL(2))
            ELSE
              PRINT*, '#',EVENTS,WGHT
            END IF
          END IF

          IF(EVENTS.LT.FLUENC.OR.WGHT.GT.WMIN) GOTO 10
          PRINT*, DOSE

        END DO ! IDOSE=1,NDOSES
      END IF          ! DUMMY block for readability: Main Loop  777777

      IF(8.EQ.8) THEN ! DUMMY block for readability: Margins 8888888
        IF(DEBUG(1)) PRINT*, 'Begin of Block 8'

        NCOLS=2*NDOSES+4
        NMAX(1)=0
        DO K=1,NCOLS
          TGMEAN(K)=ZERO
          SUMS(K)=ZERO
          IF(K.GT.2) NTARGT(K)=NTARGT(K-2)
          DO J=1,NTARGT(K)
            TGMEAN(K)=TGMEAN(K)+PMARG(J,K)*(J-1)
            SUMS(K)=SUMS(K)+PMARG(J,K)
            IF(PMARG(J,K).GT.WMIN.AND.J.GT.NMAX(1)) NMAX(1)=J
          END DO
        END DO ! DO K=1,NCOLS
        PRINT*, 'Distributions averages (#hit targets)'
        WRITE(*,'(20f10.4)') (SUMS(J),J=1,NCOLS)
        WRITE(*,'(20f10.4)') (TGMEAN(J),J=1,NCOLS)
        WRITE(*,*) TGMEAN(ICOLSE(1))/TGMEAN(ICOLIC(1)),
     &             TGMEAN(ICOLSE(2))/TGMEAN(ICOLIC(2))
        DO K=1,NCOLS-4
          IF(MOD(K,2).EQ.1) THEN
            TGMEAN(K)=TGMEAN(K)/TGMEAN(ICOLSE(1))
          ELSE
            TGMEAN(K)=TGMEAN(K)/TGMEAN(ICOLSE(2))
          END IF
        END DO
```

----- FORTRAN source code of program ME_ROI_3P -----

```
          WRITE(*,'(20f10.4)') (TGMEAN(J),J=1,NCOLS-4)

        END IF          ! DUMMY block for readability: Margins 8888888

        IF(9.EQ.9) THEN ! DUMMY block for readability: OUTPUT  9999999
C!        #Write results to output file
          FILOUT='SEB_'//FILENM
          PRINT*, 'Write output to '//FILOUT
          OPEN(LUN,FILE=FILOUT,STATUS='UNKNOWN')
          WRITE(LUN,*) '*** Output from PROGRAM ME_ROI_3P Version '
     &             //VDATE//' on '//TSTAMP()
          WRITE(LUN,*) 'Filename: '//FILOUT
          WRITE(LUN,*) HEADER
          WRITE(LUN,*) 'Correlations P1 and F2 for PVALUE= ',PVALUE
          WRITE(LUN,*) NMAXSE(1), NMAXSE(2)
          DO I=1,NMAXSE(1)
            WRITE(LUN,'(1000F15.10)') (BVARSE(I,J),J=1,NMAXSE(2))
          END DO
          CLOSE(LUN)

          FILOUT='SEC_'//FILENM
          PRINT*, 'Write output to '//FILOUT
          OPEN(LUN,FILE=FILOUT,STATUS='UNKNOWN')
          WRITE(LUN,*) '*** Output from PROGRAM SE_ROI_3C Version '
     &             //VDATE//' on '//TSTAMP()
          WRITE(LUN,*) 'Filename: '//FILOUT
          WRITE(LUN,*) HEADER
          WRITE(LUN,*) 'Correlations P1 and F2 for PVALUE= ',PVALUE
          WRITE(LUN,*) NMAXSE(1), NMAXSE(2)
          DO I=1,NMAXSE(1)
            WRITE(LUN,'(1000F15.10)') (CORRSE(I,J),J=1,NMAXSE(2))
          END DO
          CLOSE(LUN)

          PREFIX='MEP_'
          FILOUT=PREFIX//FILENM
          PRINT*, 'Write output to '//FILOUT
          OPEN(LUN,FILE=FILOUT,STATUS='UNKNOWN')
          WRITE(LUN,*) '*** Output from PROGRAM ME_ROI_3P Version '
     &             //VDATE//' on '//TSTAMP()
          WRITE(LUN,*) 'Filename: '//FILOUT
          WRITE(LUN,*) HEADER
          WRITE(LUN,*) 'Single event distribution for PVALUE= ',PVALUE

          CDOSES(1)='      P1'
          CDOSES(2)='     P2+'
          WRITE(LUN,'(A9,50a15)') '#',((CDOSES(K),K=1,2),J=1,NDOSES),
     &                         'P1','P2+','P1','P2+'
          PRINT*, DOSE
          DO I=1,NDOSES
            WRITE(CDOSES(I),'(f5.1,a2)') DOSE(I),'Gy'
          END DO

          WRITE(LUN,'(1X,a8,50a15)') 'targets',
     &            ((CDOSES(J),K=1,2),J=1,NDOSES),
     &            ('SE',K=1,2),('IC',K=1,2)
          DO I=1,NMAX(1)
            WRITE(LUN,'(1X,i8,50F15.10)') I-1,(PMARG(I,J),J=1,NCOLS)
          END DO
          CLOSE(LUN)
```

----- FORTRAN source code of program ME_ROI_3P -----
<pre>   END IF   ! DUMMY block for readability: OUTPUT   9999999<br>END DO ! IFILE=1,NFILES<br><br>END PROGRAM ! ME_ROI_3P<br>C!____<br><br>INCLUDE 'TSTAMP.f'</pre>

FORTRAN subroutine BLINIT

This subroutine initializes the Bravais lattice used for scoring clusters and is called from the programs IC_3D and ROI_3D. The respective source codes have an INCLUDE statement that expects the code in the second row of the table below in an Ascii-File named BLINIT.f.

----- FORTRAN subroutine BLINIT -----

```
C!**********************************************************************
      SUBROUTINE BLINIT(NAZMTH,NDIV,DLATC,SRDATE)
C!    ***************************************************************
C!    Initialization of the base vectors of the reziprocal lattices
C!    ***************************************************************
C!    This routine calculates the elements of the array ADJNTB in the
C!    common block LATTICE, i.e. the set of base vectors of the
C!    reciprocal lattice of a cubic fcc Bravais lattice for different
C!    orientations of the primary particle's trajectory with respect to
C!    the Bravais lattice of target volumes.
C!    25.02.2020: The algorithm for deterministic sampling has been
C!    implemented such that for NDIV>0 the spherical triangle defined by
C!    the north pole and the azimuths +/- 2/3*pi on the equator is
C!    divided into four congruent triangles, then these smaller
C!    triangles are again divided into four congruent triangles etc.
C!    until finally we have 4^NDIV triangles. The centers of gravity of
C!    these triangles define directions of the primary particle's
C!    trajectory that are homogenously distributed over the section of
C!    the unit sphere that is sufficient to consider owing to the
C!    symmetry of the cubic fcc Bravais lattice of target volumes.
C!    In addition, also a rotation of the track in itself is considered
C!    to improve statistics.
C!    Declarations ---------------------------------------------------
      IMPLICIT NONE
C!    ----- Parameters: Version Date <<<<<<<<<<<<<<<<<<<<<<<<<<<<<<<<<<<
      CHARACTER VDATE*11
      PARAMETER(VDATE='23-MAY-2021') !<<<<<<<<<<<<<<<<<<<<<<<<<<<<<<<<<<
C!    23-MAY-2021 HR: Added outout of version date to calling program
C!    23-OCT-2020 HR: Fixed bug in initialization of reciprocal basis
C!    15-MAR-2020 HR: Added VDATE and modified COMMON BLOCK VERBOSE
C!    ----- Parameters: General purpose constants
      REAL*8 ONE, TWO, THREE, FOUR, ZERO
      PARAMETER(ONE=1.0, TWO=2.0, THREE=3.0, FOUR=4.0, ZERO=0.0)
C!    ----- Parameters for lattice orientation
      INTEGER*4 MAZMTH, MDIV, MTHETA, MDIR
      PARAMETER(MAZMTH=1, MDIV=0, MTHETA=2**MDIV,
     &           MDIR=MAZMTH*(MTHETA*(MTHETA+1))/2)
C!    ----- Local scalars
      REAL*8 PIBY2, PIBY3, ROOT2, ROOT3 ! Constants
      REAL*8 COSTHM, COSTHP, DCOSTH, DPHI2, DPHI1, PHI1, PHI2, THETA
      INTEGER*4 IAZ, IDIR, IR, IS, J, K, L, NTHETA
C!    ----- Local arrays
      REAL*8 ADJBAS(3,3), REULER(3,3)
C!    ----- Global variables
      INTEGER*4 NDIR
      REAL*8 ADJNTB(3,3,MDIR)
```

----- FORTRAN subroutine BLINIT -----

```
      COMMON /LATTICE/ ADJNTB, NDIR
C!    ----- Scalars and arrays passed from the calling module
      INTEGER*4 NAZMTH, NDIV
      REAL*8 DLATC
C!    ----- Scalars and arrays passed to the calling module
      CHARACTER*11 SRDATE
C!    ------
      LOGICAL DEBUG(4), SRFLAG
      COMMON /VERBOSE/ DEBUG, SRFLAG
C!<<<<End declarations<<<<<<<<<<<<<<<<<<<<<<<<<<<<<<<<<<<<<<<<<<<<<<<

      IF(1.EQ.1) THEN ! Dummy block: Checks for consistency
        PRINT*, 'SUBROUTINE BLINIT Version: '//VDATE
        IF(DEBUG(1)) PRINT*, 'BLINIT 1'
        IF(NAZMTH.GT.MAZMTH) THEN
          PRINT*, 'ERROR in BLINIT: NAZMTH > MAZMTH ',NAZMTH,MAZMTH
          STOP
        END IF
        IF(NDIV.GT.MDIV) THEN
          PRINT*, 'ERROR in BLINIT: NDIV > MDIV ',NDIV,MDIV
          STOP
        END IF
        SRDATE=VDATE
      END IF          ! Dummy block: Checks for consistency

      IF(2.EQ.2) THEN ! Dummy block for readability: INITIALIZATION
        IF(DEBUG(1)) PRINT*, 'BLINIT 2'
        ROOT2=SQRT(2.0)
        ROOT3=SQRT(3.0)
        ADJBAS(1,1)=ONE/DLATC/ROOT3
        ADJBAS(2,1)=-ONE/DLATC
        ADJBAS(3,1)=-ONE/DLATC/ROOT2/ROOT3
        ADJBAS(1,2)=ONE/DLATC/ROOT3
        ADJBAS(2,2)=ONE/DLATC
        ADJBAS(3,2)=-ONE/DLATC/ROOT2/ROOT3
        ADJBAS(1,3)=ZERO
        ADJBAS(2,3)=ZERO
        ADJBAS(3,3)=ONE/DLATC*ROOT3/ROOT2

        PIBY2=ATAN(ONE)*TWO
        PIBY3=PIBY2*TWO/THREE
      END IF          ! Dummy block for readability: INITIALIZATION

      PRINT*, ADJBAS
      IF(3.EQ.3) THEN ! Dummy block for readability: Get track direction
                      ! and adjust lattice orientation accordingly
        IF(DEBUG(1)) PRINT*, 'BLINIT 3'
        NTHETA=2**NDIV                  ! number of polar angles
        NDIR=NAZMTH*NTHETA*NTHETA       ! number of orientations
        DCOSTH=ONE/REAL(NTHETA)         ! step size in cos theta
        COSTHP=ONE-TWO/THREE*DCOSTH     ! cos theta plus (see section 
paper)
        COSTHM=ONE-FOUR/THREE*DCOSTH    ! cos theta minus (see section 
paper)
        DPHI1=FOUR*PIBY2/NAZMTH         ! step for rotation of track

        PHI1=ZERO
        IDIR=0
        DO IAZ=1,NAZMTH
          DO K=1,NTHETA ! Centers of upward pointing triangles
            DPHI2=TWO*PIBY3/REAL(K) ! step of trajectory azimuth
```

----- FORTRAN subroutine BLINIT -----

```
          PHI2=(ONE/REAL(K)-ONE)*PIBY3 ! first azimuth value
          THETA=ACOS(COSTHP)
          DO J=1,K
            IDIR=IDIR+1
        IF(DEBUG(1)) PRINT*, 'BLINIT vor CALL EULERM'
            CALL EULERM(REULER,THETA,PHI1,PHI2)
        IF(DEBUG(1)) PRINT*, 'BLINIT nach CALL EULERM',REULER
            DO IR=1,3
               DO IS=1,3
                 ADJNTB(IR,IS,IDIR)=ZERO
                 DO L=1,3
                   ADJNTB(IR,IS,IDIR)=ADJNTB(IR,IS,IDIR)
     &                                   +REULER(IR,L)*ADJBAS(L,IS)
                 END DO ! IS=1,3
               END DO ! IR=1,3
            END DO
            PHI2=PHI2+DPHI2  ! increment trajectory azimuth
          END DO ! J=1,K
          COSTHP=COSTHP+DCOSTH  ! increment cosine of polar angle
        END DO ! K=1,NTHETA

      IF(DEBUG(1)) PRINT*, 'BLINIT 3.2 IAZ', IAZ
        DO K=1,NTHETA-1 ! Centers of downward pointing triangles
          DPHI2=TWO*PIBY3/REAL(K) ! step of trajectory azimuth
          PHI2=(ONE/REAL(K)-ONE)*PIBY3 ! first azimuth value
          THETA=ACOS(COSTHM)
          DO J=1,K
            IDIR=IDIR+1
            CALL EULERM(REULER,THETA,PHI1,PHI2)
            DO IR=1,3
               DO IS=1,3
                 ADJNTB(IR,IS,IDIR)=ZERO
                 DO L=1,3
                   ADJNTB(IR,IS,IDIR)=ADJNTB(IR,IS,IDIR)
     &                                  +REULER(IR,L)*ADJBAS(L,IS)
                 END DO ! IS=1,3
               END DO ! IR=1,3
            END DO
            PHI2=PHI2+DPHI2  ! increment trajectory azimuth
          END DO ! J=1,K
          COSTHM=COSTHM+DCOSTH  ! increment cosine of polar angle
        END DO ! K=1,NTHETA
        PHI1=PHI1+DPHI1 ! increment azimuth for track rotation
      END DO ! IAZ=1,NAZMTH
    END IF          ! Dummy block: Adjust lattice orientation

    END SUBROUTINE BLINIT
C!____________________________________________________________________________

C!************************************************************************
      SUBROUTINE EULERM(REULER,THETA,PHI1,PHI2)
C!    **************************************************************
C!    Calculates combination of three inverse Euler rotation matrices
C!    **************************************************************
C!    Declarations ---------------------------------------------------
      IMPLICIT NONE
C!    ----- Scalars and arrays passed from the calling module
      REAL*8 PHI1,PHI2,THETA
      REAL*8 REULER(3,3)
C!    ----- Local scalars
      INTEGER*4 I,J
```

----- FORTRAN subroutine BLINIT -----

```
      REAL*8 RCOS, RSIN
C!    ----- Local arrays
      REAL*8 ROT1(3,3), ROT2(3,3), ROT3(3,3), RAUXIL(3,3)
C!<<<<End declarations<<<<<<<<<<<<<<<<<<<<<<<<<<<<<<<<<<<<<<<<<<<<<<<
      DO I=1,3
        DO J=1,3
          IF(I.EQ.J) THEN
            REULER(I,J)=1.0
            ROT1(I,J)=1.0
            ROT2(I,J)=1.0
            ROT3(I,J)=1.0
          ELSE
            REULER(I,J)=0.0
            ROT1(I,J)=0.0
            ROT2(I,J)=0.0
            ROT3(I,J)=0.0
          ENDIF
          RAUXIL(I,J)=0.0
        END DO
      END DO

      RCOS=COS(PHI2)
      RSIN=SIN(PHI2)
      ROT1(1,1)=RCOS
      ROT1(2,2)=RCOS
      ROT1(1,2)=RSIN
      ROT1(2,1)=-RSIN

      RCOS=COS(THETA)
      RSIN=SIN(THETA)
      ROT2(1,1)=RCOS
      ROT2(3,3)=RCOS
      ROT2(1,3)=RSIN
      ROT2(3,1)=-RSIN

      RCOS=COS(PHI1)
      RSIN=SIN(PHI1)
      ROT3(1,1)=RCOS
      ROT3(2,2)=RCOS
      ROT3(1,2)=RSIN
      ROT3(2,1)=-RSIN

      CALL MMULT(ROT2,ROT1,RAUXIL)
      CALL MMULT(ROT3,RAUXIL,REULER)
      END SUBROUTINE EULERM
C!______________________________________________________________________

C!**********************************************************************
      SUBROUTINE MMULT(A,B,C)
C!    *****************************************************************
C!    Matrix multiplication C=A*B
C!    *****************************************************************
      REAL*8 A(3,3), B(3,3), C(3,3)
      INTEGER*4 I, J, K
      DO I=1,3
        DO K=1,3
          C(I,K)=0.0
          DO J=1,3
            C(I,K)=C(I,K)+A(I,J)*B(J,K)
          END DO
        END DO
```

----- FORTRAN subroutine BLINIT -----

```
      END DO
      END SUBROUTINE MMULT
C!________________________________________________________________________

C!**********************************************************************
      SUBROUTINE MVMULT(A,B,C)
C!    *****************************************************************
C!    Multiplication of matrix A and vector B: C=A*B
C!    *****************************************************************
      REAL*8 A(3,3), B(3), C(3)
      INTEGER*4 I, J
      DO I=1,3
        C(I)=0.0
        DO J=1,3
          C(I)=C(I)+A(I,J)*B(J)
        END DO
      END DO
      END SUBROUTINE MVMULT
C!________________________________________________________________________
```

FORTRAN subroutine RFINIT

This subroutine interpretes the character string encoding the data structure of the lines of the track simulation output files read by IC_3D and ROI_3D.

----- FORTRAN subroutine RFINIT -----

```
      SUBROUTINE RFINIT()
C!    Sets the information for the columns of the input file
C!
C!    ------ Global variables
C!    ------
C!     CODE: Character string encoding the meaning of the entries in a
C!           line of the input file as follows:
C!           'T'     - number of the primary particle track
C!           'X','Y','Z' - x, y, and z coordinates of the transfer point
C!           'E'     - energy deposit (if applicable)
C!           'I'     - ionization cluster size (if applicable)
C!           '%'     - additional data that are not used
C!           Example: The string 'TZ%%EXY ' indicates that there are 7
C!                  entries in each line, of which the first is the
C!                  track number, the second the z coordinate, the
C!                  fifth the energy, and the sixth and seventh the x
C!                  and y coordinates
C!     IDT: Array of the columns indices of (1-3) x,y,z coordinates
C!                 (4) energy deposit (if present), (5) track number,
C!                 (6) number of ionizations in cluster (if present)
C!                 (7-8) are there for future use
      CHARACTER CODE*8
      INTEGER*2 IDT(8),NDT
      COMMON /RFORMT/IDT,NDT,CODE
C!    Local variables
      INTEGER*1 J

      DO J=1,8
       IDT(J)=0
      END DO

      DO J=1,8
```

```
----- FORTRAN subroutine RFINIT -----
        IF(CODE(J:J).EQ.'X'.AND.IDT(1).EQ.0) IDT(1)=J
        IF(CODE(J:J).EQ.'Y'.AND.IDT(2).EQ.0) IDT(2)=J
        IF(CODE(J:J).EQ.'Z'.AND.IDT(3).EQ.0) IDT(3)=J
        IF(CODE(J:J).EQ.'E'.AND.IDT(4).EQ.0) IDT(4)=J
        IF(CODE(J:J).EQ.'T'.AND.IDT(5).EQ.0) IDT(5)=J
        IF(CODE(J:J).EQ.'I'.AND.IDT(6).EQ.0) IDT(6)=J
        IF(CODE(J:J).NE.' ') NDT=J
      END DO

      IF(IDT(1).EQ.0) PRINT*, 'No data column for X'
      IF(IDT(2).EQ.0) PRINT*, 'No data column for Y'
      IF(IDT(3).EQ.0) PRINT*, 'No data column for Z'
      IF(IDT(5).EQ.0) PRINT*, 'No data column for track number'
      IF(IDT(1)*IDT(2)*IDT(3)*IDT(5).EQ.0) STOP

      END
```

FORTRAN function TSTAMP

This function gets the system date and time and returns a 24 character text string in the format YYYY-MM-DD HH:MM ±HH:MM, where the latter is the time difference to UTC.

```
----- FORTRAN subroutine TSTAMP -----
      CHARACTER*24 FUNCTION TSTAMP()
      CHARACTER DATE*8, TIME*10, ZONE*5, TIMEST*24
      INTEGER*4 IDT(8)
      CALL DATE_AND_TIME(DATE,TIME,ZONE,IDT)
C!              123456789 123456789 1234
      TIMEST = 'DD-MMM-YYYY HH:MM +HH:MM'
      TSTAMP=DATE(7:8)//'-'//DATE(5:6)//'-'//DATE(1:4)//' '//
     &       TIME(1:2)//':'//TIME(3:4)//' '//ZONE(1:3)//':'//ZONE(4:5)
      RETURN
      END
```

Sample debug options file for use with programs IC_3D and ROI_3D

This is simply an text files with four lines containing either .TRUE. or .FALSE.

```
----- Sample debug options file for use with programs IC_3D and ROI_3D -----
.FALSE.     ! DEBUG(1) --> Main program
.FALSE.     ! DEBUG(2) --> TARGTS main sections
.FALSE.     ! DEBUG(3) --> TARGTS IDIR loop
.FALSE.     ! DEBUG(4) --> TARGTS IPOS loop details
```

References

Braunroth T, Nettelbeck H, Ngcezu S A, Rabus H (2020) Three-dimensional nanodosimetric characterisation of proton track structure. Radiation Physics and Chemistry 176(0):109066

Schneider U, Vasi F, Schmidli K, Besserer J (2019) Track Event Theory: A cell survival and RBE model consistent with nanodosimetry. Radiation Protection Dosimetry 18317-21

Schneider U, Vasi F, Schmidli K, Besserer J (2020) A model of radiation action based on nanodosimetry and the application to ultra-soft X-rays. Radiat Environ Bioph 59(3):1-12
